# Two novel potential pathogens for soybean

**DOI:** 10.1371/journal.pone.0221416

**Published:** 2019-08-22

**Authors:** Andressa Cristina Zamboni Machado, Priscila Moreira Amaro, Santino Aleandro da Silva

**Affiliations:** Department of Plant Protection, Instituto Agronômico do Paraná, Londrina, Paraná, Brazil; National Institute of Technology Rourkela, INDIA

## Abstract

Nematode diseases have a worldwide importance for several economic agricultural crops, such as soybean. Frequently, new or secondary pathogens arise as emergent diseases due to the intensified use of agricultural lands, causing extensive yield losses. *Helicotylenchus dihystera* (Hd) and *Scutellonema brachyurus* (Sb) appear as potential pathogens for soybean in Brazil, since their spread and population densities have been increased on soybean growing areas. Aiming to evaluate the quantitative distribution of Hd and Sb in soybean fields in South Brazil, a survey was conducted during the growing seasons of 2014–2015 and 2015–2016 in which 1,088 soil samples and 1,043 root samples were analyzed. Besides, two greenhouse experiments were conducted to evaluate the pathogenicity of both nematodes to soybean plants, in comparison with *P*. *brachyurus* (Pb), a known pathogen of the crop. The survey demonstrated that Pb, Hd and Sb are widely distributed in the States of Paraná, Santa Catarina and Mato Grosso do Sul. Besides, we proved that Hd and Sb multiply and cause root lesions in soybean cv. Potência, since they were found inside roots, and can be considered as potential pathogens for soybean plants.

## Introduction

The expansion of problems caused by nematodes in Brazil, especially in the Cerrado region, probably is due to the intensified use of agricultural lands, with monoculture of susceptible crops, which increase soil nematode populations along the crops. The economy in this region is based largely on the viability of soybean, maize, and cotton [1].

Root-knot nematodes (RKNs) (*Meloidogyne* spp. Goeldi 1929) and the soybean cyst nematode (SCN), *Heterodera glycines* Ichinoe, represent the most economically important nematodes for soybean crop in Brazil and worldwide [2]. In the Cerrado region, after the impact caused by *H*. *glycines*, two important changes occurred, the use of alternative plants for crop rotation and the increase in the no-tillage system [3]. These changes directly affected *Pratylenchus brachyurus* (Godfrey 1929) Filipjev & Sch. Stekhoven 1974 populations, which currently is a concern as one of the main problems to soybean in Brazilian fields [4, 1].

On soybean, yield losses caused by *P*. *brachyurus* can reach 30 to 50%, with higher damages detected on sand soils (< 20% clay) [5]. Non-specific symptoms can be easily overlooked or mistaken with damages caused by other soil pathogens or attributed to other causes such as nutrient deficiency or drought [6].

Recently, other species, as *Helicotylenchus dihystera* (Cobb) Sher and *Scutellonema brachyurus* Andrassy, appear as potential pathogens for soybean in Brazil, since their spread and population densities have been increased on soybean growing areas [7, 8]. The intensified use of agricultural lands in Brazil could have a significant paper in the emerging of secondary pathogens in agricultural crops [1].

*Scutellonema brachyurus* has been observed especially in the States of Paraná, Mato Grosso do Sul and Maranhão, in soybean fields showing stunting plants associated with yield losses [7]. Soil nematode populations can reach up to 5,000 specimens per 100 cm^3^ of soil in samples collected in soybean fields. Apparently, injuries are associated with drought and the use of early cultivars [7].

*Helicotylenchus dihystera*, mainly associated with crop losses on sugarcane [9] and maize [10], has also been detected in soybean growing areas in Brazil, with increasing incidences and soil populations [1]. Surveys conducted in Acre, Mato Grosso, and Goiás States showed that *H*. *dihystera* was found in 85%, 92%, and 47% of the samples, respectively [11, 12]. Generally, its presence has been associated with stunted plants, but direct damages were not quantified, as well as the threshold levels.

The parasitism of *H*. *dihystera* in soybean plants was already reported by Orbin [13], that stained soybean roots infected with *H*. *dihystera* and observed adults, larvae and eggs within the root cortex associated with small brown lesions in the immediate vicinity of the nematodes. However, because of the lack of persistent burrows, the author suggested that *H*. *dihystera* was not a significant pathogen of soybean.

In order to evaluate the quantitative distribution of *H*. *dihystera* and *S*. *brachyurus* in soybean fields from the States of Paraná, Mato Grosso do Sul and Santa Catarina, a survey was conducted during the growing seasons of 2014–2015 and 2015–2016 in which 1,088 soil samples and 1,043 root samples were analyzed. Besides, two greenhouse experiments were conducted to evaluate the pathogenicity of both nematodes on soybean plants, in comparison with *P*. *brachyurus*, a known pathogen of the crop.

## Materials and methods

### Nematode survey

**Fig 1:**
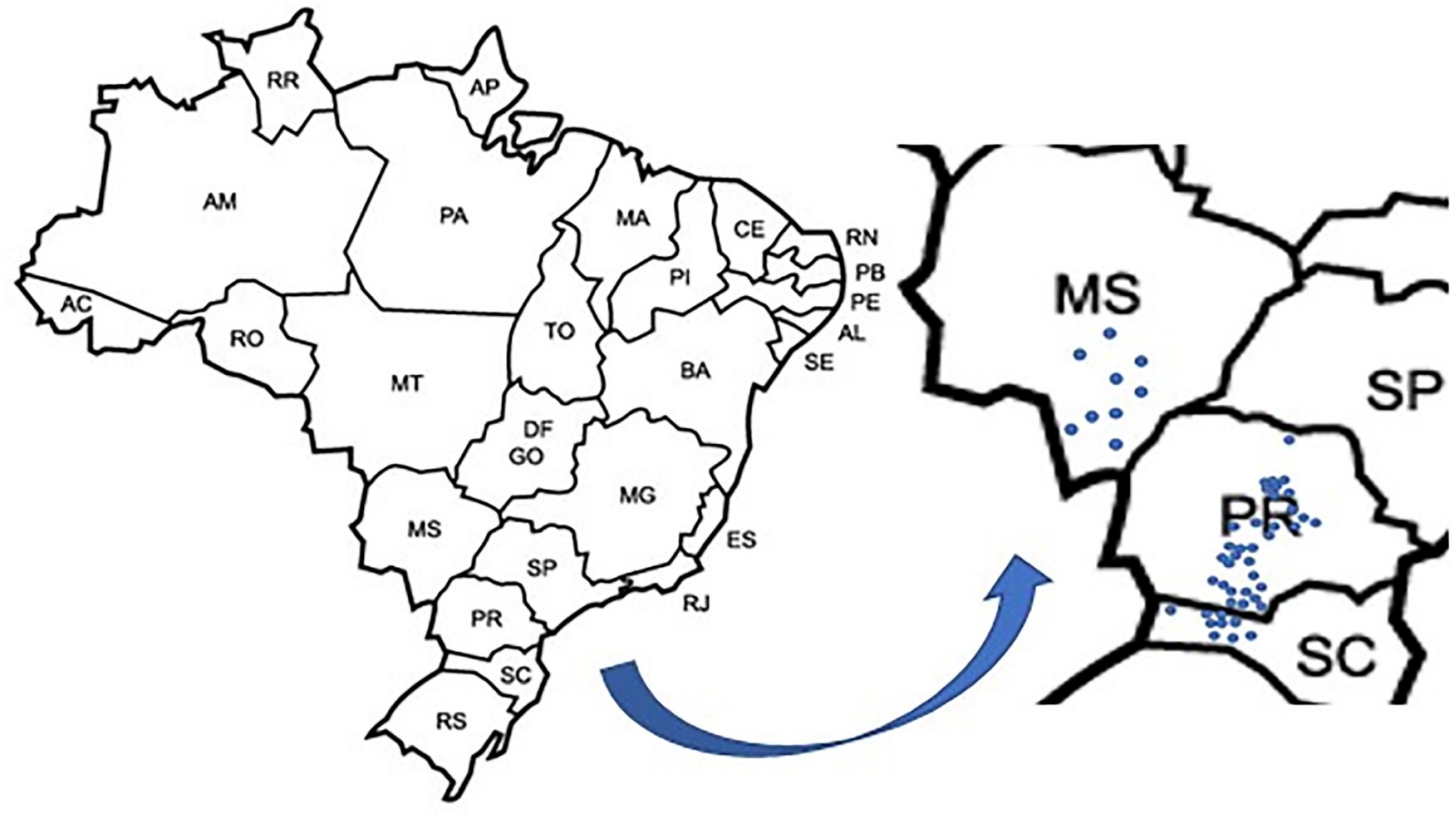
Locations of soybean fields surveyed to determine the occurrence and distribution of *Pratylenchus brachyurus*, *Helicotylenchus dihystera* and *Scutellonema brachyurus* in South Brazil.

Surveys were undertaken in commercial soybean fields in the States of Paraná (33 municipalities sampled), Mato Grosso do Sul (9 municipalities) and Santa Catarina (9 municipalities), in the Southern Brazil (Fig 1), between October 2014 to April 2016. For that purpose, soybean roots together with the rhizosphere (#1,043) and bulk soils (#1,088) were sampled. Samples were collected with a hack from the upper 20 cm of soil of four to five plants chosen arbitrarily in each field. All surveyed fields were representative of soybean growing areas in Southern Brazil; they were selected based on indicative parameters of their commercial importance and of previous problems with nematodes, according with the grower’s experience. Frequency of infestation and population density of plant-parasitic nematodes were determined. The frequency of infestation was calculated as the percentage of samples in which the nematode species was diagnosed.

The entire root system was washed free of soil, weighed and used to extract nematodes by the maceration procedure described in literature [14] without the use of sodium hypochlorite. Nematodes present in the soil samples were extracted from a 50 cm^3^ subsample using the Baermann funnel method [15]. Nematodes were counted and identified by morphological traits to genus. Selected live adult female specimens of each genera were mounted on microscope slides with water for species identification. *Pratylenchus brachyurus* (Pb), *Helicotylenchus dihystera* (Hd) and *Scutellonema brachyurus* (Sb) were identified through morphological and morphometrical approaches, based on measurements of 10 adult females from each population [6, 16, 17].

Characters measured were: body length (L), stylet length (St), height (Hstk) and width (Wstk) of stylet knobs, distance from posterior end to vulva (V), distance from vulva to anus (V-A), tail length (T), post-uterine branch length (PUB), dorsal esophageal gland orifice (DGO), maximum body diameter (BD), body diameter at anus (BDA) and vulva (BDV) to Pb; L, V, lip height (LH), lip diameter (LD), St, conus length (CL), DGO, pharynx (P), excretory pore (EP), BD, T, and BDA to Hd; and L, V, DGO, St, metenchium length (ML), telenchium length (TL), P, EP, pharyngeal overlap (PO), BD, BDA, medium bulb length (MBL) and diameter (MBD), LH, LD, lateral field width (LFW), T, scutellum length (SCL) and width (SCW), and spermatheca length (SPL) and width (SPW) to Sb.

Besides, species delimitation was undertaken using an integrated approach considering morphological and morphometric evaluation combined with molecular sequence analyses [18]. For molecular analyses, specimens were picked individually from the water suspension with the aid of a stereomicroscope and stored in saline solution (NaCl 1M). DNA from individual females of each population was extracted using the methodology described in literature [19]. ITS-1 and D2/D3 rDNA amplifications were performed using the universal primers 18S (5’- TTG ATT ACG TCC CTG CCC TTT -3’) and RN58SR (5’- ACG AGC CGA GTG ATC CAC CG -3’) to ITS-1 region, located respectively in the 18S and 5.8S rDNA [20], and D2A (TCGGAAGGAACCAGCTACTA) and D3B (ACAAGTACCGTGAGGGAAAGTTG) to D2/D3 region.

For PCR, 21 μl of the master mix Platinum PCR Supermix (Invitrogen) were added to a 0.2 ml microcentrifuge tube; after, 2 μl of DNA and 1 μl of each primer were added. Amplifications conditions for ITS-1 were as follows: 94 °C for 2 min 45 s followed by 40 cycles of 94 °C for 1 min, 57 °C for 45 s and 72 °C for 2 min, and a final extension of 72 °C for 10 min; for D2/D3: 94 °C for 5 min followed by 35 cycles of 94 °C for 30 s, 55 °C for 45 s and 72 °C for 2 min, and a final extension of 72 °C for 10 min. Purified DNA fragments were sequenced on a sequencer ABI 377 DNA Sequencer and the quality of sequences was checked using the software BioEdit. Detailed protocols were described by our colleagues [21] (Pb), [22] (Hd) and [17] (Sb). The obtained D2/D3 sequences of each population species used for greenhouse experiments were submitted to the GenBank database under accession numbers: EF693897 (Pb), MG365905 (Sb) and MG760573 (Hd).

### Greenhouse assays

Two greenhouse experiments were carried out at the Instituto Agronômico do Paraná, Londrina, Paraná State, Brazil (23°18’36”S, 51°09’46”W). The trials were conducted from 11 November 2016 to 13 January 2017 with the temperature ranging from 22 °C to 41 °C.

Seeds of soybean cultivar BMX Potência were sown in 500 cm^3^ plastic pots containing 400 cm^3^ of sterilized (160°C/5hours) soil. Seedlings were thinned to one per pot prior to nematode inoculation. *Pratylenchus brachyurus* population used as inoculum was collected in São Luís, Maranhão State, Brazil (02°31’48”S, 44°18’10”W), from soybean plants (*Glycine max*), identified through morphological [6] and molecular [23] techniques and subsequently cultured under greenhouse conditions in soybean cv. BMX Potência. *Helicotylenchus dihystera* and *Scutellonema brachyurus* populations were collected in Fênix, Paraná State, Brazil (23°54’57”S, 51°58’44”W), both from soybean plants, identified through morphological and molecular approaches [16, 17] and subsequently cultured under greenhouse conditions in soybean cv. BMX Potência.

After nematode extraction from soybean roots by the maceration procedure described in literature [14], without the use of sodium hypochlorite, a suspension containing 1,000 mixed life stages (initial population, IP) was pipetted into two small 2.0–4.0 cm-deep holes beside the root system of 5 days-old seedlings. Plants in pots were watered as needed and fertilized once with 3 g of Osmocote^®^ Plus (15% N, 9% P2O5, 12% K2O, 1% Mg, 2.3% S, 0.05% Cu, 0.45% Fe, 0.06% Mn, 0.02% Mo).

The final nematode population (FP) was evaluated 15, 30 and 70 days after inoculation (DAI). At 15 and 30 DAI, plants were removed from pots and root systems were washed carefully and stained with fuchsin acid [24]. The entire root system of each plant was examined under light microscope. Besides, pots were immersed in a bucket containing 4 L of water to separate roots from soil. This entire suspension was used to extract nematodes from the soil of each replicate by Baermann funnel method [15]. At 70 DAI, roots were washed with tap water, dried on absorbent paper, values of fresh weight determined and, subsequently, cut in 1 cm pieces; the entire root system of each replicate was processed for nematode extraction, as described earlier. Nematodes were also extracted from soil in this date [15]. FP was estimated by counting mixed life stages from parasitized roots and soil with the aim of a Peter´s slide. The reproductive factor (RF = FP (root+soil)/ IP) values were subsequently determined. Number of nematodes per gram of roots (Nema/g) was also calculated for each replicate. In all evaluations, fresh top weights were determined.

Experiments were arranged in completely randomized design, with treatments corresponding to each nematode species and 8 replicates. Data were submitted to variance analysis and, for data residuals normalization, BoxCox procedure indicated that data transformation by ln(y+0.01) was necessary. Treatments were compared by Fisher´s Least Significant Difference (LSD) Test (P ≤ 0.05), using the R 2.15.2 program [25], packages MASS [26] and Agricolae [27].

## Results

**Fig 2:**
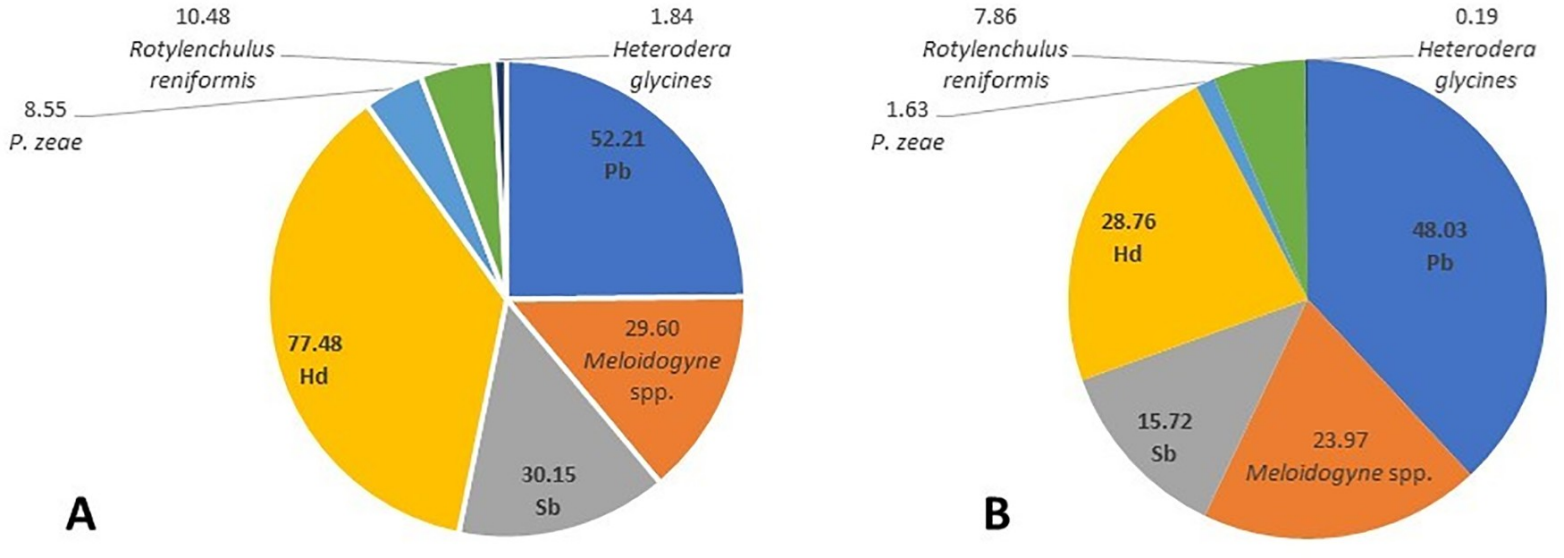
Frequency of occurrence of nematode species in the samples, highlighting the frequency of *Pratylenchus brachyurus*, *Helicotylenchus dihystera* and *Scutellonema brachyurus* in soil (A) and root (B) samples.

Pb was found in 501 root samples (48.03%) and in 568 soil samples (52.21%), Hd in 300 root (28.76%) and in 843 soil (77.48%) samples, and Sb in 164 root (15.72%) and in 328 soil (30.15%) samples (Fig 2). The population densities of Pb ranged from 1 to 1,852 Nema/g and from 1 to 1,060 nematodes per 50 cm^3^ of soil; for Hd, densities ranged from 4 to 168 Nema/g and from 1 to 4,284 nematodes per 50 cm^3^ of soil, whereas to Sb densities ranged from 3 to 379 Nema/g and from 1 to 11,712 nematodes per 50 cm^3^ of soil. Generally, growers in these localities described symptoms of stunting and chlorotic plants, associated with lower productivities, but no quantifications were done, since other nematode species occurred in the samples (Fig 2) and the isolation of damages caused by each one is impossible.

**Fig 3:**
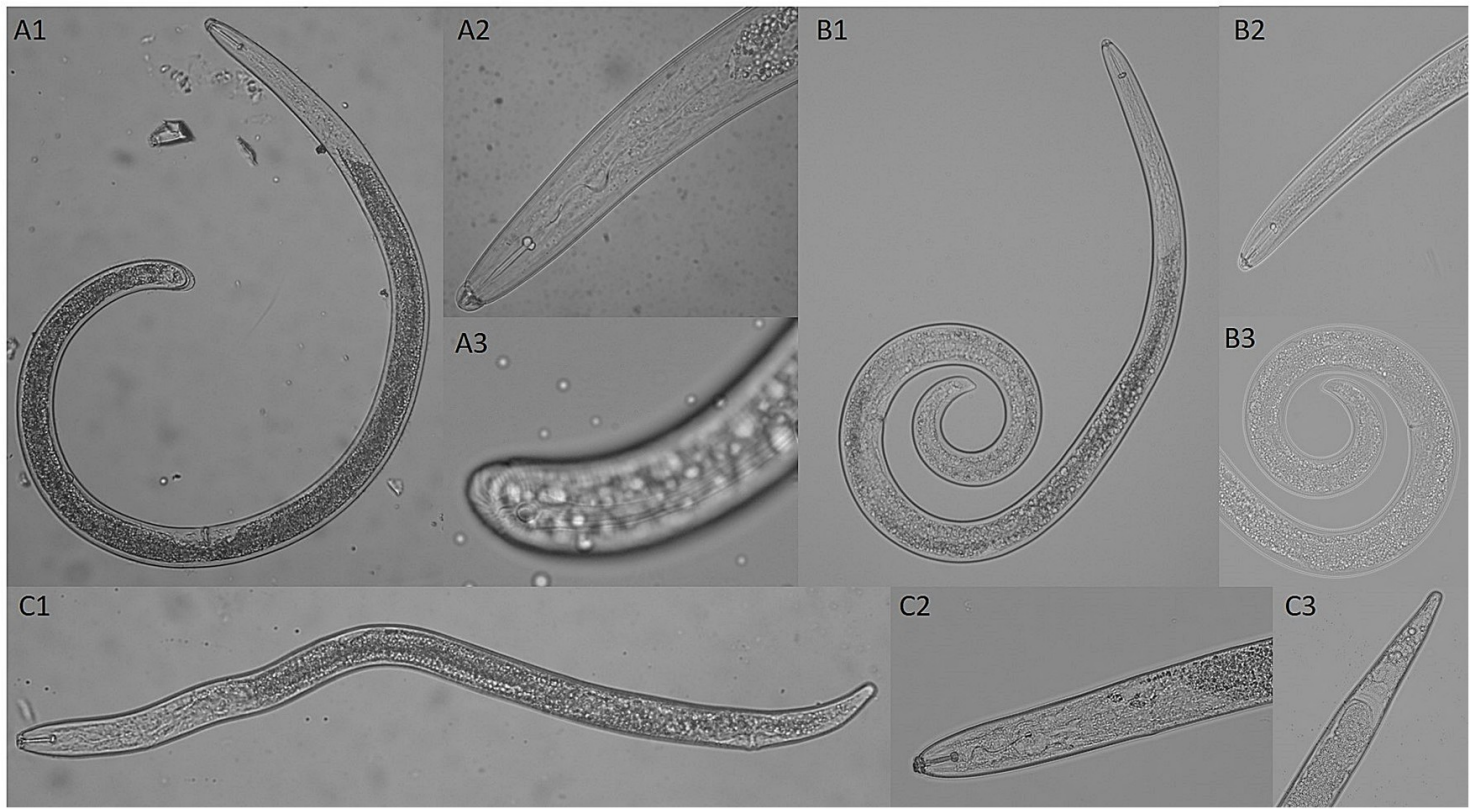
General morphology of females of *Pratylenchus brachyurus* (Pb), *Helicotylenchus dihystera* (Hd) and *Scutellonema brachyurus* (Sb) populations from Brazil. A1) Sb entire body; A2) Sb anterior portion of body with labial region in evidence; A3) Sb tail with circular scutellum in evidence; B1) Hd entire body; B2) Hd anterior portion of body with labial region in evidence; B3) Hd tail; C1) Pb entire body; C2) Pb anterior portion of body with labial region in evidence; C3) Pb tail.

Except for slightly differences in tail shape, morphology of specimens of Pb, Hd and Sb (Fig 3) compare very well with the previous descriptions of these species. In relation to morphometric taxonomic characters of females, for *P*. *brachyurus* values measured, in μm (mean ± standard deviation), were: L = 543 (± 19.50), BD = 24 (± 2.20), BDV = 21 (± 1.43), BDA = 15 (± 1.24), V% = 82 (± 4.55), St = 19 (± 0.81), Wstk = 4 (± 0.29), Hstk = 3 (± 0.44), DGO = 2.78 (± 0.10), V-A = 54 (± 4.37), T = 28 (± 1.52), and PUB = 16 (± 2.41). For *H*. *dihystera* females, values measured, in μm (mean ± standard deviation), were:: L = 601 (± 44.30), V = 209 (± 23.58), LH = 4 (± 0.32), LD = 7 (± 0.48), St = 26 (± 1.01), CL = 12 (± 0.86), DGO = 13 (± 1.01), P = 142 (± 8.30), EP = 108 (± 6.94), BD = 25 (± 1.83), T = 16 (± 1.63), and BDA = 16 (± 1.27). Finally, for *S*. *brachyurus* females, values measured, in μm (mean ± standard deviation), were:: L = 740 (± 30.06), V = 285 (± 18.67), DGO = 8 (± 0.62), St = 29 (± 1.23), ML = 14 (± 1.17), TL = 12 (± 1.11), P = 149 (± 8.03), EP = 116 (± 9.32), PO = 23 (± 2.39), BD = 29 (± 1.27), BDA = 19 (± 0.72), MBL = 12 (± 0.78), MBD = 10 (± 0.56), LH = 6 (± 0.26), LD = 9 (± 0.36), LFW = 4 (± 0.48), T = 4 (± 1.01), SCL = 5 (± 0.49), SCW = 4 (± 0.58), SPL = 11 (± 1.09), and SPW = 10 (± 0.92).

**Fig 4:**
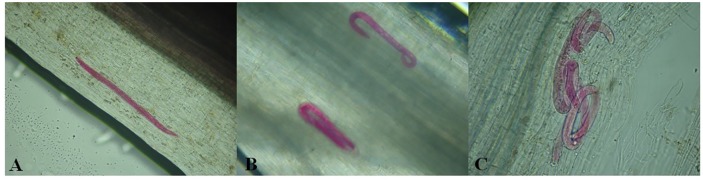
Nematode-infected soybean roots stained with fuchsin acid. A) *Pratylenchus brachyurus*; B) *Helicotylenchus dihystera*; C) *Scutellonema brachyurus*.

**Fig 5:**
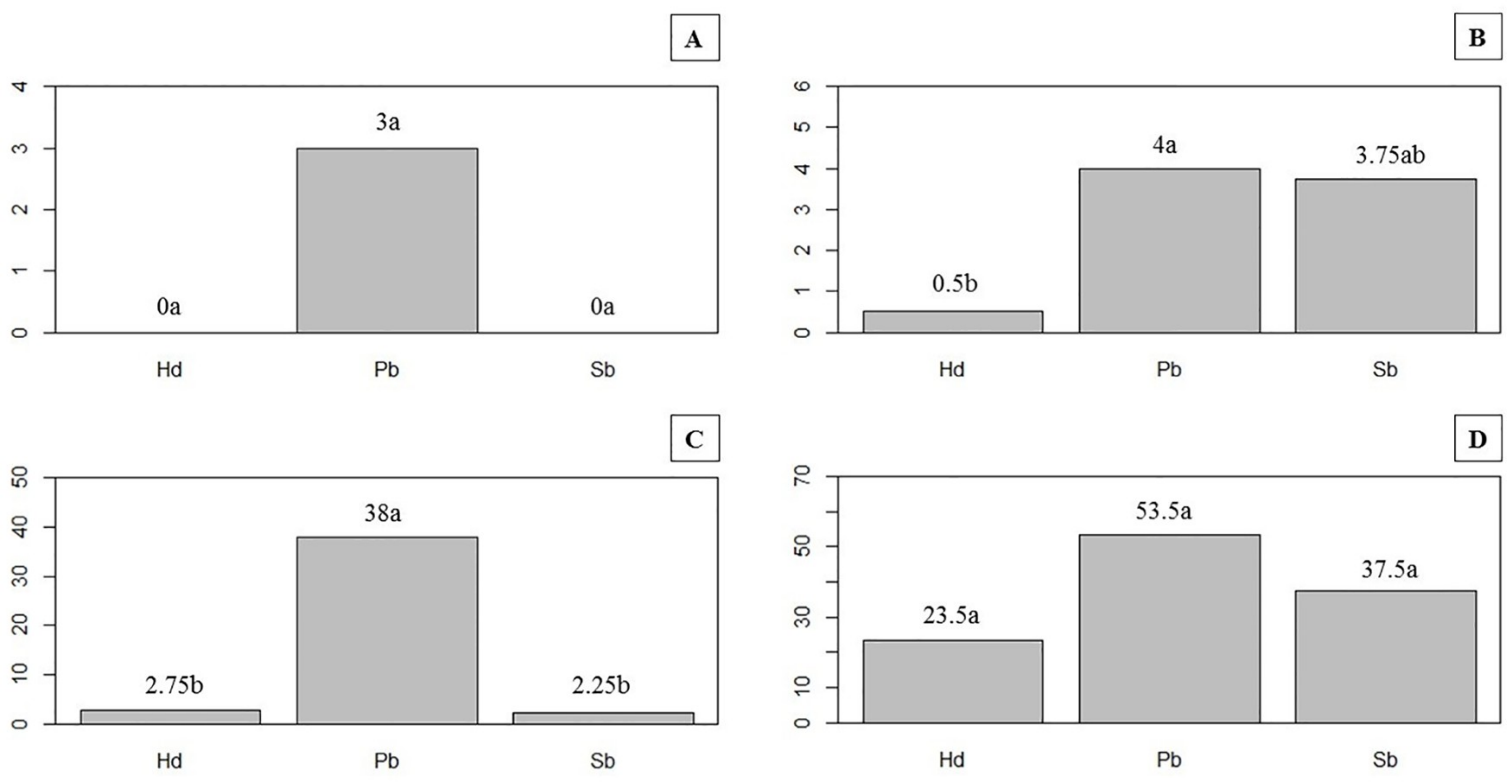
Number of nematodes inside roots of soybean cv. Potência. A and B) Experiments 1 and 2, respectively, 15 days after inoculation; C and D) Experiments 1 and 2, respectively, 30 days after inoculation. Hd = *Helicotylenchus dihystera*; Pb = *Pratylenchus brachyurus*; Sb = *Scutellonema brachyurus*.

At 15 and 30 DAI, the observation of roots stained with fuchsin acid showed that the three nematode species penetrated soybean roots cv. Potência (Figs 4 and 5). In experiment 1, number of penetrated nematodes was lower than those found in experiment 2 (Fig 5A and 5B). At 15 DAI (Fig 5A), it was observed a lower number of nematodes inside roots than at 30 DAI (Fig 5B), as expected.

In relation to the number of nematodes in soil (Fig 5C and 5D), we found a higher number of exemplars of *H*. *dihystera* and *S*. *brachyurus*, in relation to *P*. *brachyurus*, at 15 DAI in soil, in both experiments (Fig 5C). Inversely, at 30 DAI, the number of *P*. *brachyurus* in soil was higher than those from *H*. *dihystera* and *S*. *brachyurus* in experiments 1 and 2 (Fig 5D).

At 70 DAI, nematodes multiplied in soybean (Table 1), especially Hd and Sb, with RF values higher than 1.0 in both experiments. In experiment 1, Hd showed the highest RF value in soybean cv. Potência (RF = 2.33), followed by Sb (1.16); RF value for Pb was lower than 1.0 (0.63), although this cultivar is reported to be susceptible to this root-lesion nematode species. In relation to the number of nematodes per gram of roots, in experiment 1 we observed the lowest number to Hd (29), probably as a consequence of the lowest value of FRW in this treatment. In experiment 2, soybean plants inoculated with Hd showed the highest numbers of nema/g and RF of this species. The total number of Pb found in soil (Table 1) was low in both experiments, while Hd showed the highest values of SP. In relation to the total number of nematodes in roots (Table 1), Pb and Sb showed the highest values in both experiments and Hd, the lowest.

**Table 1:** Soybean development under inoculation with *Pratylenchus brachyurus* (Pb), *Helicotylenchus dihystera* (Hd) and *Scutellonema brachyurus* (Sb) and nematodes multiplication.

Measured variables	Check	Pb	Hd	Sb
**Exp. 1**				
FRW^a^	19.77 a	11.58 b	10.22 b	11.43 b
FTW^b^	40.75 a	27.18 bc	22.56 c	31.00 ab
Soil population	-	10 c	2,041 a	346 b
Root population	-	620 a	288 b	818 a
Total population^c^	-	630 b	2,329 a	1,164 a
RF^d^	-	0.63 b	2.33 a	1.16 a
Nema/g^e^	-	53 a	29 b	82 a
**Exp. 2**				
FRW^a^	30.58 a	29.39 a	35.07 a	33.21 a
FTW^b^	45.00 b	44.35 b	57.07 a	56.92 a
Soil population	-	22 c	2,849 a	576 b
Root population	-	1,743 a	168 c	463 b
Total population^c^	-	1,765 b	3,017 a	1,039 b
RF^d^	-	1.76 b	3.01 a	1.03 b
Nema/g^e^	-	63 ab	86 a	34 b

Each value represents the mean of 8 replicates. Means followed by the same letter in the column did not differ according with the LSD test at 5% of significance.

^a^Fresh root weight;

^b^Fresh top weight;

^c^Number of nematodes in soil + roots;

^d^Reproduction factor;

^e^Number of nematodes per gram of roots.

In relation to the development of soybean plants inoculated with Pb, Hd and Sb at 70 dai (Table 1), the FRW was significantly affected by nematodes in experiment 1, since FRW values were lower than that obtained in non-inoculated plants. In this experiment, FRW was more affected by Pb and Hd, with the lowest values, while plants infected with Sb showed intermediate value of FRW. In experiment 2, the effect of nematodes in the development of soybean roots was not observed, probably due to the better experimental conditions in this case.

For the variable FTW (Table 1), plants inoculated with Pb and Hd in experiment 1 showed significantly lower values than those inoculated with Sb or the check plants. In experiment 2, higher values of FTW were observed in plants inoculated with Hd and Sb, in relation to Pb and check plants. This could had occurred due to the better development of roots in experiment 2, which possibly allowed better development of the aerial parts of plants.

**Fig 6:**
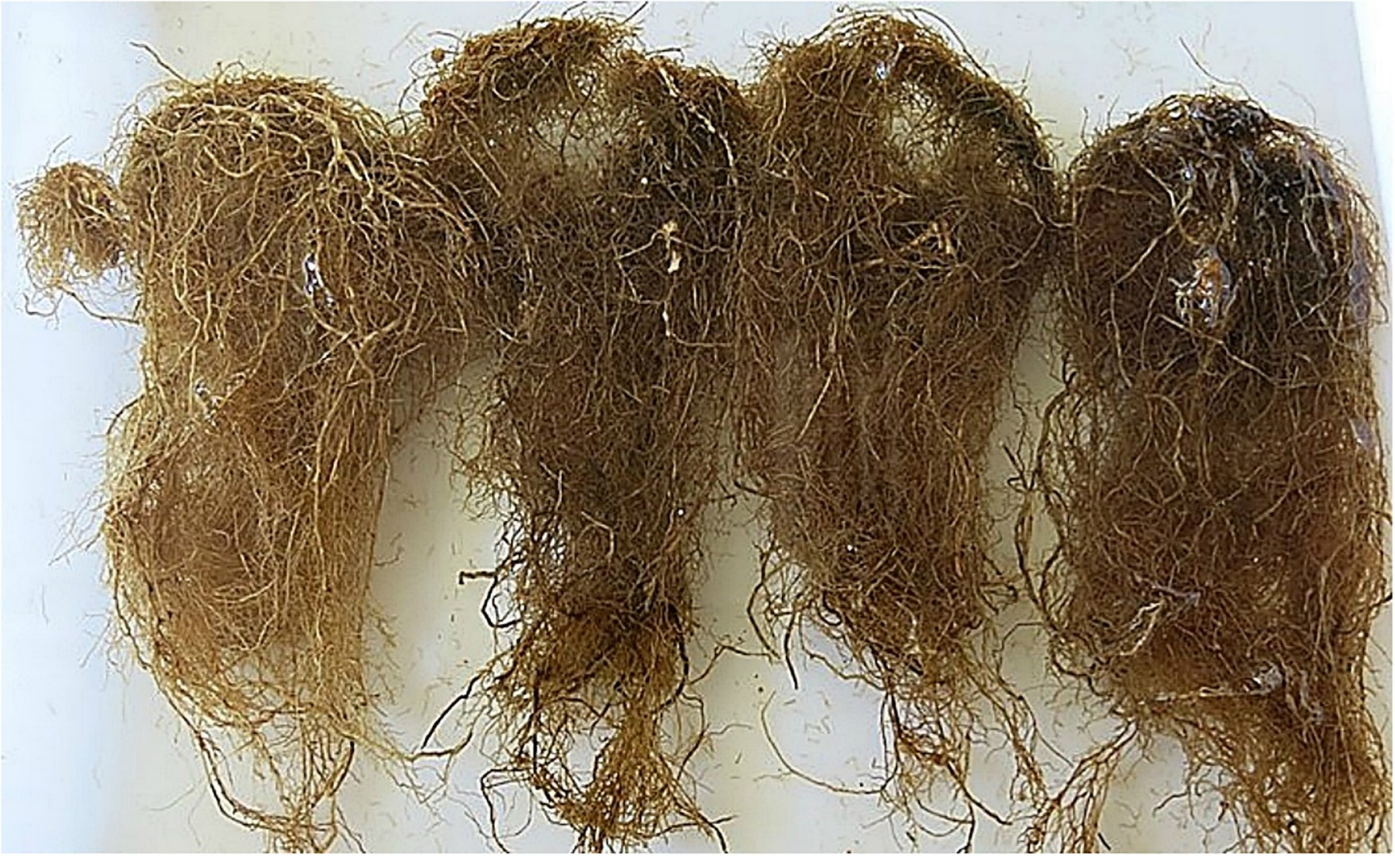
Root lesions and darkening of soybean roots observed in plants inoculated with *Pratylenchus brachyurus* (Pb), *Scutellonema brachyurus* (Sb) and *Helicotylenchus dihystera* (Hd). From left to right: check (without nematode), Pb, Sb and Hd.

It was also observed a general darkening in the root systems of soybean cv. Potência inoculated with Pb, Hd and Sb at 70 DAI, probably as a consequence of the coalescence of several lesions caused by the nematodes (Fig 6). Symptoms attributed to Hd and Sb were very similar to those reported for Pb.

## Discussion

In our study, we demonstrated that Pb, Hd and Sb are widely distributed in the States of Paraná, Santa Catarina and Mato Grosso do Sul, as observed in another Brazilian soybean growing regions [1]. Besides, we proved that Hd and Sb cause root lesions in soybean and can be considered as potential pathogens for soybean plants.

In Goiás State, Brazil, high population densities of Hd have been reported in corn growing areas [28] and, posteriorly, it was observed that Hd was the prevalent nematode species in coffee, corn and tomato growing areas in the State of Goiás and in the Federal District [29]. In Rio de Janeiro State, Hd was found in 30% of the samples collected in organic farms [30]. In sugarcane fields from Pernambuco State, Hd was also the dominant species [31, 32]. Hd was the prevalent nematode species in forest fragments and agricultural areas cropped with corn [33] and sugarcane [34] in Paraná State. In Mato Grosso State, it was observed that Hd was present in 97% and 81% of the soil and root samples, respectively, collected in cotton growing areas [35].

In relation to soybean, in Mato Grosso State, from 3,000 samples analyzed by the laboratory from Fundação Rio Verde, Hd was found in 92% of them [12]. Hd was reported in 78% of the samples collected in soybean fields in Rio Grande do Sul State [36] and in 47% of the samples collected in Goiás State [37]. Hd was also reported in 59% of the soybean samples collected in Paraná State in a previous study [38]. Our results corroborate with the several reports of prevalence of Hd in soil samples, since it was present in 77.48% of the collected samples, with densities reached more than 4,000 nematodes per 50 cm^3^ of soil.

Sb has been found in different regions from the States of Paraná, Mato Grosso do Sul and Maranhão, with population densities higher than 5,000 per 100 cm^3^ of soil and even higher in 10 g of roots [7]. Sb also was found in 23% from the samples collected in soybean fields in Paraná State in previous report [39]. In the present survey, Sb was found in 30.15% of the samples and populations reached more than 11,000 nematodes per 50 cm^3^ of soil, higher than the densities reported previously in soybean fields [7].

In relation to Pb, it was present in more than 50% of the soil samples in the present survey. This species has become a concern among cotton and soybean growers in the Cerrado region and nowadays is considered the main nematode species to soybean in Brazil [4, 1]. On soybean, losses caused by this nematode can reach to 30 to 50% of the yield, in which higher damages are observed in sand soils (< 20% clay) [5]. Symptoms are stunting of plants and leaf yellowing, besides the root lesions, characteristics from this genus [1]. We observed stunted and chlorotic plants, associated with lower productivities in the surveyed fields, but no quantifications or correlations with the nematode densities were done.

As observed previously [13], Hd can be found inside the soybean roots, causing brown lesions, contradicting the idea that this nematode is a migratory ectoparasitic or semi-endoparasitic species that may occur in very high numbers only in soil surrounding host roots [40]. Indeed, the majority of studies reported only the association of Hd with several plant species, including soybean, but no symptoms or damages have been quantified [41, 42, 38, 43]. The general darkening of roots observed in our study configurate a more aggressive symptom than those previously reported [13], confirming the hypothesis that Hd, together with Sb, can be considered as a potential pathogen to soybean in Brazil.

Similarly, Sb has been considered as a primarily ectoparasitic [44] associated with a range of agricultural and horticultural crops [17]. In our study, we observed the penetration of this species in soybean roots, as observed for Hd. Reports of the parasitism or association between Sb and soybean are scarce in worldwide literature [17], but in some regions of Brazil, as Paraná State, this nematode has been frequently found in soil and root samples collected in soybean fields [39, 7].

Both nematodes could facultatively had developed the migratory endoparasitic habit in order to better adaptation in hosts used in a cropping system, as the intensified use of agricultural lands provide roots along the year for nematode parasitism and adaptation. As an example, the evolution of plant parasitism in nematodes is reported for *Aphelenchoides* spp., which feed and reproduce on plant leaf mesophyll and on buds (as a migratory endoparasite) but can also feed on fungi [45]. Recently, this group was reported as the causal agent of a new soybean disease in Brazil, the soybean green stem and foliar retention syndrome [46], and also causing similar symptoms in cotton plants [47], which shows the great plasticity of *Aphelenchoides* spp. in adaptation to hosts imposed in the cropping systems. The same plasticity could be suggested to exist in Hd and Sb.

Since Hd and Sb were found inside soybean roots, root lesions and generalized darkening of root systems were observed, as a result of this type of parasitism (as showed in Fig 6). Root lesions caused by Sb in soybean were not yet reported in literature. Although losses were not quantified, it is expected to occur a reduction in soybean productivity when it is cropped in infested areas with Hd and Sb, as observed for Pb, since both penetrated and caused damages on roots, multiplied on soybean, and, therefore, considering their widespread, can be considered as potential pathogens for this crop and possibly to others, which comprise the cropping systems in Brazil.

## Supporting information

S1 File Data analysis for experiment 1, evaluation at 15 days after inoculation. (PDF)

S2 File Data analysis for experiment 2, evaluation at 15 days after inoculation. (PDF)

S3 File Data analysis for experiment 1, evaluation at 27 days after inoculation. (PDF)

S4 File Data analysis for experiment 2, evaluation at 27 days after inoculation. (PDF)

S5 File Data analysis for plant development in experiment 1, evaluation at 70 days after inoculation. (PDF)

S6 File Data analysis for nematode multiplication in experiment 1, evaluation at 70 days after inoculation. (PDF)

S7 File Data analysis for plant development in experiment 2, evaluation at 70 days after inoculation. (PDF)

S8 File Data analysis for nematode multiplication in experiment 2, evaluation at 70 days after inoculation. (PDF)
