# Supplementary material for: Two novel potential pathogens for soybean
Source: PLoS One. 2019 Aug 22;14(8):e0221416. doi: 10.1371/journal.pone.0221416 (PMC6705753; doi:10.1371/journal.pone.0221416)
Supplement: S1 File — (PDF) [file pone.0221416.s001.pdf]

15_DAI_experiment_1.R

Santino

Tue Jul 23 18:49:47 2019

```
rm(list = ls())
cs1<-read.table("C:\\analises nemato\\soja comparativo analises\\soja
comp 15 emb.txt",h=T,dec=",")
cs1
```

```
##    trat baer pen
## 1    Pb   21   9
## 2    Pb   16   3
## 3    Pb   38   0
## 4    Pb   21   0
## 5    Sb  203   0
## 6    Sb  375   0
## 7    Sb  389   0
## 8    Sb  446   0
## 9    Hd  444   0
## 10   Hd  554   0
## 11   Hd  453   0
## 12   Hd  560   0
```

```
data.frame(table(cs1$trat))
```

```
##   Var1 Freq
## 1   Hd    4
## 2   Pb    4
## 3   Sb    4
```

```
attach(cs1)
```

```
# mean and median
```

```
(Medias = with(cs1 [ 3], aggregate(. ~trat, data=cs1[ 3], mean)))
```

```
##   trat pen
## 1   Hd   0
## 2   Pb   3
## 3   Sb   0
```

```
(Medianas = with(cs1 [ 3], aggregate(. ~trat, data=cs1[ 3], median)))
```

```
##   trat pen
## 1   Hd 0.0
## 2   Pb 1.5
## 3   Sb 0.0
```

```
#standard deviation
sd(cs1$pen)

## [1] 2.662876

#variation coef
require(raster)
cv(cs1$pen, na.rm=TRUE)

## [1] 266.2876

#nematodes in roots

cs1p<-aov(cs1$pen~cs1$trat)
cs1p

## Call:
##    aov(formula = cs1$pen ~ cs1$trat)
##
## Terms:
##                 cs1$trat Residuals
## Sum of Squares        24        54
## Deg. of Freedom        2         9
##
## Residual standard error: 2.44949
## Estimated effects may be unbalanced

summary(cs1p)

##             Df Sum Sq Mean Sq F value Pr(>F)
## cs1$trat     2     24      12       2  0.191
## Residuals    9     54       6

par(mfrow=c(2,2)); plot(cs1p); layout(1)
```

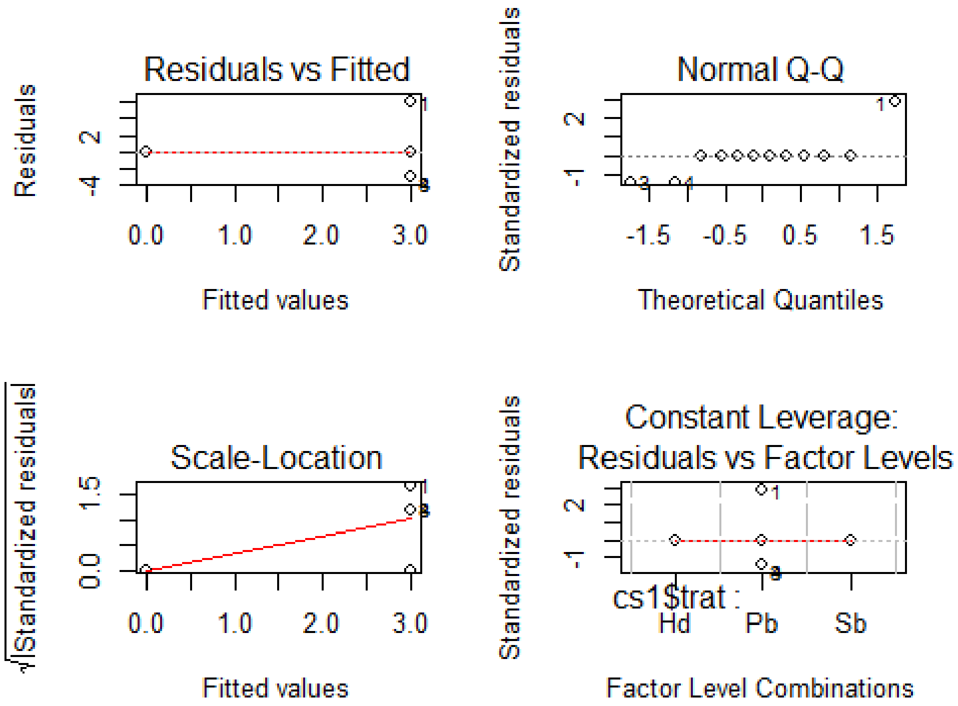


```
shapiro.test(cs1p$res)

##
##  Shapiro-Wilk normality test
##
## data:  cs1p$res
## W = 0.65002, p-value = 0.0002898

plot(pen ~ trat, data = cs1)
```

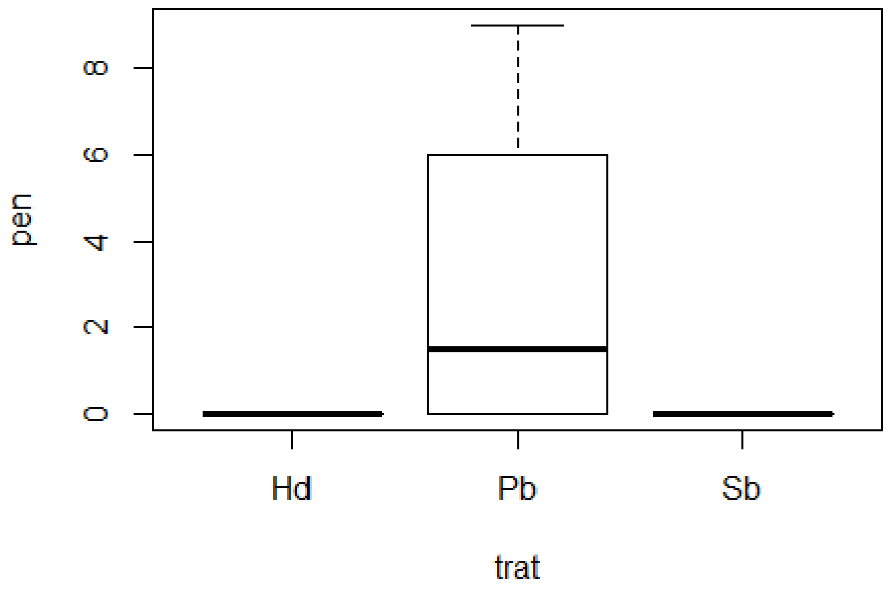


```
bartlett.test(cs1$pen, cs1$trat)

##
##  Bartlett test of homogeneity of variances
##
## data:  cs1$pen and cs1$trat
## Bartlett's K-squared = Inf, df = 2, p-value < 2.2e-16

# Transforma??o Box-Cox
boxcox(pen+0.01 ~ trat, data=cs1, plotit=T)
```

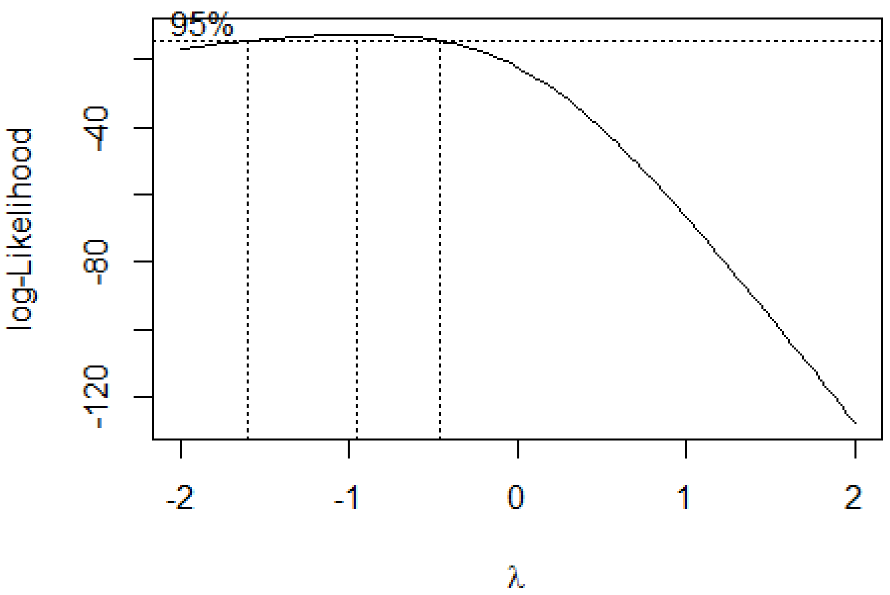


```
bc <- boxcox(pen+0.01 ~ trat, data=cs1, lam=seq(-2, .5, 1/10))
```

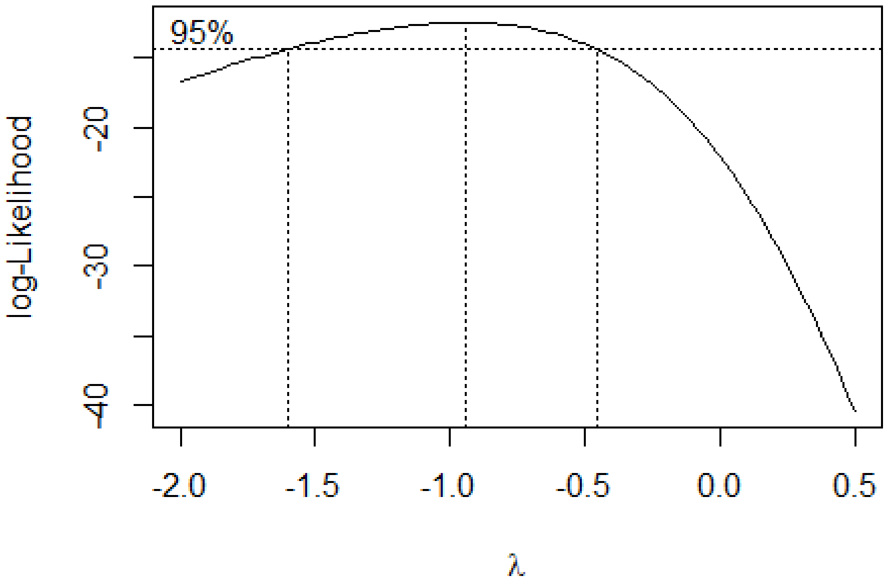


```
(lambda = bc$x[which.max(bc$y)])
```

```
## [1] -0.9393939
```

```r
n2<-log(cs1$pen+0.01)
```

```r
cs1p1<-aov(n2~cs1$trat)
cs1p1
```

```
## Call:
##    aov(formula = n2 ~ cs1$trat)
##
## Terms:
##                  cs1$trat Residuals
## Sum of Squares   26.08592  39.72992
## Deg. of Freedom         2         9
##
## Residual standard error: 2.101056
## Estimated effects may be unbalanced
```

```r
summary(cs1p1)
```

```
##             Df Sum Sq Mean Sq F value Pr(>F)
## cs1$trat     2  26.09  13.043   2.955  0.103
## Residuals    9  39.73   4.414
```

```r
par(mfrow=c(2,2)); plot(cs1p1); layout(1)
```

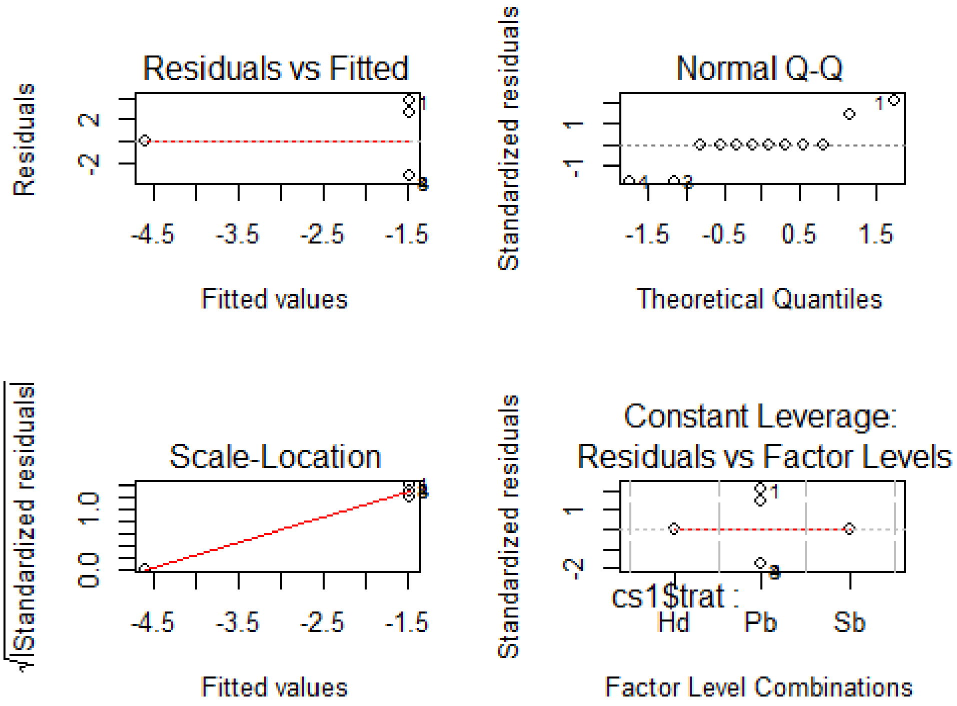


```r
shapiro.test(cs1p1$res)
```

```
##
##  Shapiro-Wilk normality test
##
## data:  cs1p1$res
## W = 0.79569, p-value = 0.008373
```

```
plot(n2 ~ trat, data = cs1)
```

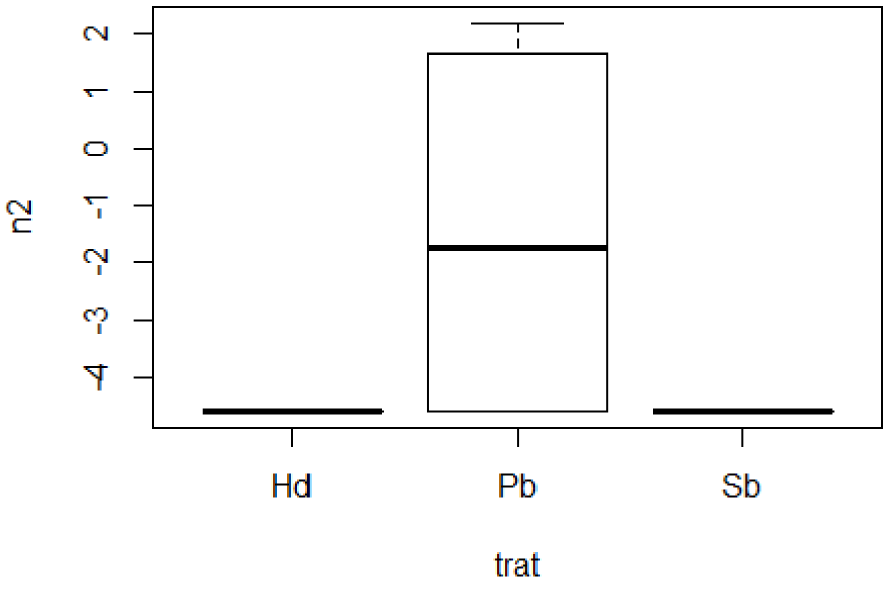


```
bartlett.test(n2, cs1$trat)
```

```
##
##  Bartlett test of homogeneity of variances
##
## data:  n2 and cs1$trat
## Bartlett's K-squared = Inf, df = 2, p-value < 2.2e-16
```

```
require(agricolae)
glr <- df.residual(cs1p1)
glr
```

```
## [1] 9
```

```
sqr <- deviance(cs1p1)
sqr
```

```
## [1] 39.72992
```

```
qmr <- sqr/glr
qmr
```

```
## [1] 4.414435
```

```
lsdn <- LSD.test(n2,cs1$trat, glr, qmr, alpha=0.05, p.adj="none")
lsdn
```

```
## $statistics
##    MSerror Df      Mean        CV  t.value      LSD
##   4.414435  9 -3.562619 -58.97503 2.262157 3.360821
##
## $parameters
##         test p.ajusted   name.t ntr alpha
##   Fisher-LSD      none cs1$trat   3  0.05
##
## $means
##           n2      std r       LCL        UCL      Min       Max
Q25
## Hd -4.605170 0.000000 4 -6.981629 -2.2287111 -4.60517 -4.605170 -
4.60517
## Pb -1.477516 3.639135 4 -3.853975  0.8989428 -4.60517  2.198335 -
4.60517
## Sb -4.605170 0.000000 4 -6.981629 -2.2287111 -4.60517 -4.605170 -
4.60517
##          Q50       Q75
## Hd -4.605170 -4.605170
## Pb -1.751615  1.376039
## Sb -4.605170 -4.605170
##
## $comparison
## NULL
##
## $groups
##           n2 groups
## Pb -1.477516      a
## Hd -4.605170      a
## Sb -4.605170      a
##
## attr(,"class")
## [1] "group"
```

```
par(mfrow=c(1,1))
pot.m <- with(cs1, tapply(pen, trat, mean))
pot.m
```

```
## Hd Pb Sb
##  0  3  0
```

```
bp <- barplot(pot.m, ylim=c(0,4))
text(bp, pot.m, label=round(pot.m, 3), pos=3)
title("15 DAI")
box()
```

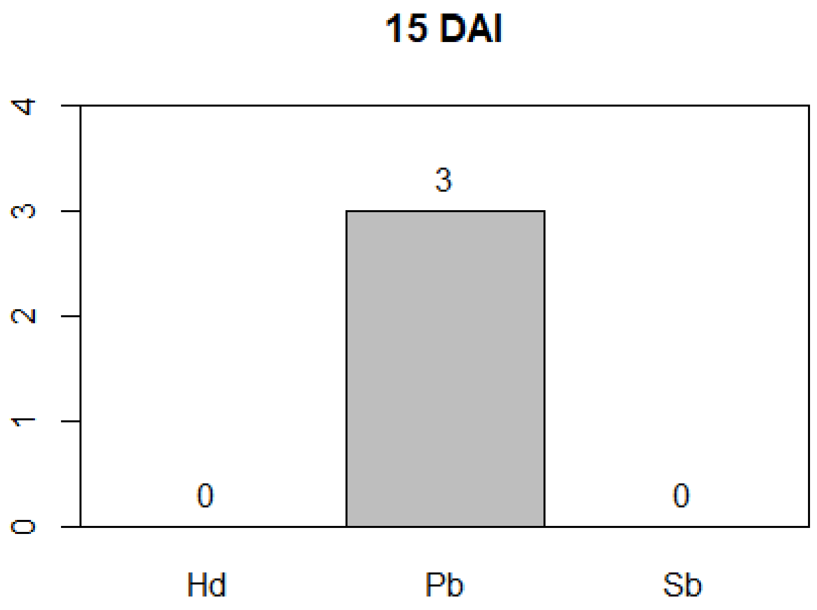
15 DAI
0
1
2
3
4
3
0
0
Hd
Pb
Sb
