# Supplementary material for: Two novel potential pathogens for soybean
Source: PLoS One. 2019 Aug 22;14(8):e0221416. doi: 10.1371/journal.pone.0221416 (PMC6705753; doi:10.1371/journal.pone.0221416)
Supplement: S2 File — (PDF) [file pone.0221416.s002.pdf]

15_DAI_experiment_2.R

Santino

Tue Jul 23 19:06:17 2019

```
rm(list = ls())
cs1<-read.table("C:\\analises nemato\\soja comparativo analises\\soja
comp 15 esq.txt",h=T,dec=",")
cs1
```

```
##    trat baer pen
## 1    Pb   17   6
## 2    Pb   16   1
## 3    Pb   15   2
## 4    Pb   25   7
## 5    Sb  223   2
## 6    Sb  331   7
## 7    Sb  257   3
## 8    Sb  223   3
## 9    Hd  464   0
## 10   Hd  552   0
## 11   Hd  423   1
## 12   Hd  462   1
```

```
data.frame(table(cs1$trat))
```

```
##   Var1 Freq
## 1   Hd    4
## 2   Pb    4
## 3   Sb    4
```

```
attach(cs1)
```

```
# mean and median
```

```
(Medias = with(cs1 [3], aggregate(. ~trat, data=cs1[3], mean)))
```

```
##   trat  pen
## 1   Hd 0.50
## 2   Pb 4.00
## 3   Sb 3.75
```

```
(Medianas = with(cs1 [3], aggregate(. ~trat, data=cs1[3], median)))
```

```
##   trat pen
## 1   Hd 0.5
## 2   Pb 4.0
## 3   Sb 3.0
```

```
#standard deviation
sd(cs1$pen)

## [1] 2.562846

#variation coef
require(raster)
cv(cs1$pen, na.rm=TRUE)

## [1] 93.19442

#nematodes in roots

cs1n<-aov(cs1$pen~cs1$trat)
cs1n

## Call:
##    aov(formula = cs1$pen ~ cs1$trat)
##
## Terms:
##                 cs1$trat Residuals
## Sum of Squares     30.50     41.75
## Deg. of Freedom        2         9
##
## Residual standard error: 2.153808
## Estimated effects may be unbalanced

summary(cs1n)

##             Df Sum Sq Mean Sq F value Pr(>F)
## cs1$trat     2  30.50  15.250   3.287 0.0848 .
## Residuals    9  41.75   4.639
## ---
## Signif. codes:  0 '***' 0.001 '**' 0.01 '*' 0.05 '.' 0.1 ' ' 1

par(mfrow=c(2,2)); plot(cs1n); layout(1)
```

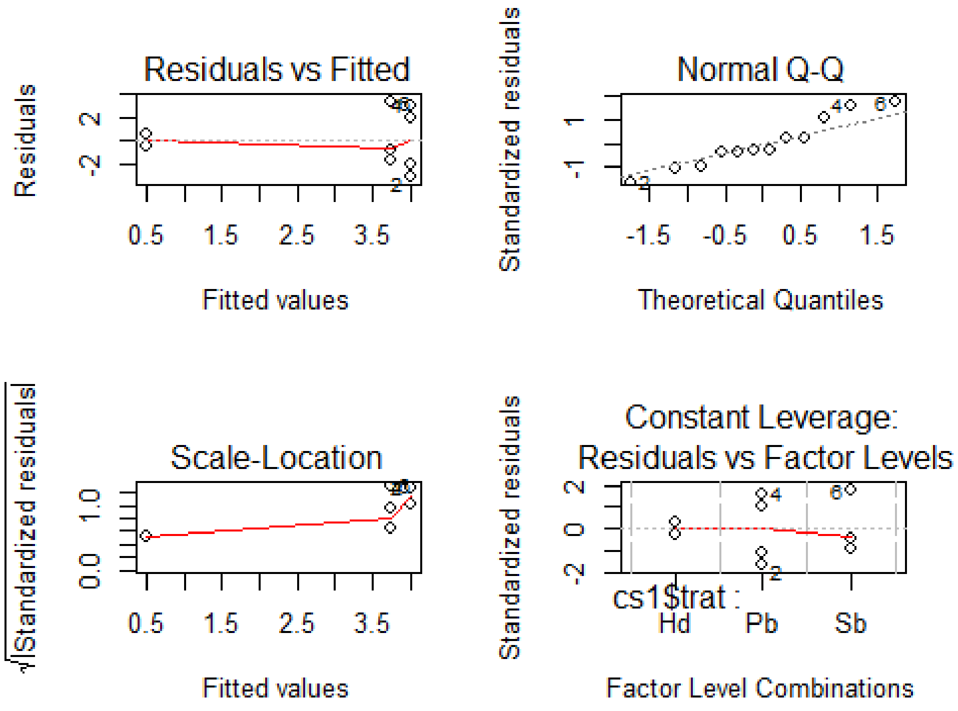


```r
shapiro.test(cs1n$res)

##
##  Shapiro-Wilk normality test
##
## data:  cs1n$res
## W = 0.94402, p-value = 0.5519

plot(pen ~ trat, data = cs1)
```

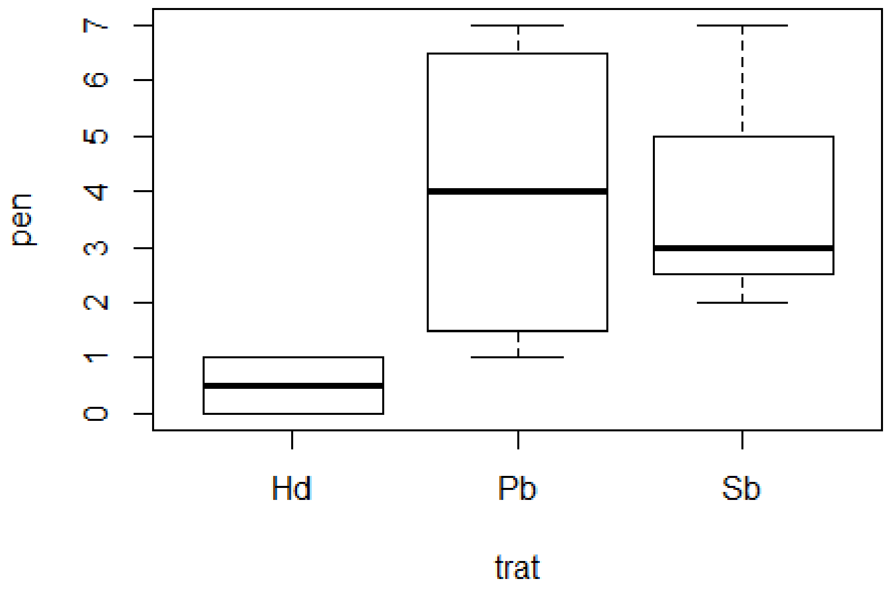


```
bartlett.test(cs1$pen, cs1$trat)

##
##  Bartlett test of homogeneity of variances
##
## data:  cs1$pen and cs1$trat
## Bartlett's K-squared = 5.095, df = 2, p-value = 0.07828

require(agricolae)
glr <- df.residual(cs1n)
glr

## [1] 9

sqr <- deviance(cs1n)
sqr

## [1] 41.75

qmr <- sqr/glr
qmr

## [1] 4.638889

lsdn <- LSD.test(cs1$pen,cs1$trat, glr, qmr, alpha=0.05, p.adj="none")
lsdn

## $statistics
##    MSerror Df Mean       CV  t.value      LSD
```

```
##   4.638889  9 2.75 78.32029 2.262157 3.445203
##
## $parameters
##         test p.ajusted   name.t ntr alpha
##   Fisher-LSD      none cs1$trat   3  0.05
##
## $means
##    cs1$pen       std r        LCL      UCL Min Max  Q25 Q50  Q75
## Hd    0.50 0.5773503 4 -1.936126 2.936126   0   1 0.00 0.5 1.00
## Pb    4.00 2.9439203 4  1.563874 6.436126   1   7 1.75 4.0 6.25
## Sb    3.75 2.2173558 4  1.313874 6.186126   2   7 2.75 3.0 4.00
##
## $comparison
## NULL
##
## $groups
##    cs1$pen groups
## Pb    4.00      a
## Sb    3.75     ab
## Hd    0.50      b
##
## attr(,"class")
## [1] "group"
```

```r
par(mfrow=c(1,1))
pot.m <- with(cs1, tapply(pen, trat, mean))
pot.m
```

```
##   Hd   Pb   Sb
## 0.50 4.00 3.75
```

```r
bp <- barplot(pot.m, ylim=c(0,6))
text(bp, pot.m, label=round(pot.m, 3), pos=3)
title("15 DAI")
box()
```

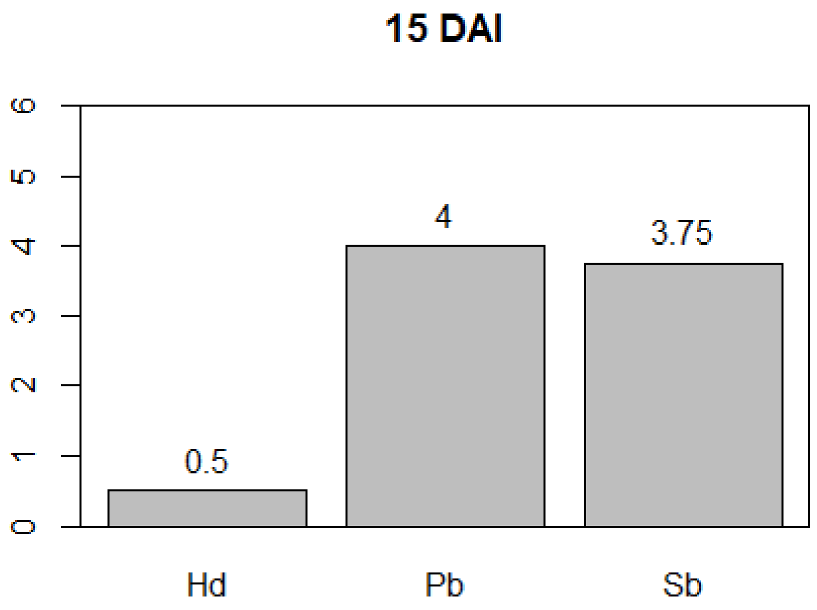
15 DAI
0
1
2
3
4
5
6
0.5
4
3.75
Hd
Pb
Sb
