# Supplementary material for: Two novel potential pathogens for soybean
Source: PLoS One. 2019 Aug 22;14(8):e0221416. doi: 10.1371/journal.pone.0221416 (PMC6705753; doi:10.1371/journal.pone.0221416)
Supplement: S3 File — (PDF) [file pone.0221416.s003.pdf]

27_DAI_experiment_1.R

Santino

Tue Jul 23 19:05:26 2019

```
rm(list = ls())
cs1<-read.table("C:\\analises nemato\\soja comparativo analises\\soja
comp 27 emb.txt",h=T,dec=",")
cs1
```

```
##    trat baer pen
## 1    Pb   76  63
## 2    Pb   38  51
## 3    Pb   41  22
## 4    Pb   27  16
## 5    Sb   11   1
## 6    Sb    9   2
## 7    Sb    5   2
## 8    Sb    7   4
## 9    Hd   12   0
## 10   Hd    7   3
## 11   Hd    5   0
## 12   Hd    7   8
```

```
data.frame(table(cs1$trat))
```

```
##   Var1 Freq
## 1   Hd    4
## 2   Pb    4
## 3   Sb    4
```

```
attach(cs1)
```

```
# mean and median
```

```
(Medias = with(cs1 [3], aggregate(. ~trat, data=cs1[ 3], mean)))
```

```
##   trat   pen
## 1   Hd  2.75
## 2   Pb 38.00
## 3   Sb  2.25
```

```
(Medianas = with(cs1 [3], aggregate(. ~trat, data=cs1[3], median)))
```

```
##   trat  pen
## 1   Hd  1.5
## 2   Pb 36.5
## 3   Sb  2.0
```

```r
#standard deviation
sd(cs1$pen)

## [1] 21.19748

#variation coef
require(raster)

cv(cs1$pen, na.rm=TRUE)

## [1] 147.8894

#nematodes in roots

cs1n<-aov(cs1$pen~cs1$trat)
cs1n

## Call:
##    aov(formula = cs1$pen ~ cs1$trat)
##
## Terms:
##                 cs1$trat Residuals
## Sum of Squares  3361.167  1581.500
## Deg. of Freedom        2         9
##
## Residual standard error: 13.25603
## Estimated effects may be unbalanced

summary(cs1n)

##             Df Sum Sq Mean Sq F value  Pr(>F)
## cs1$trat     2   3361  1680.6   9.564 0.00593 **
## Residuals    9   1582   175.7
## ---
## Signif. codes:  0 '***' 0.001 '**' 0.01 '*' 0.05 '.' 0.1 ' ' 1

par(mfrow=c(2,2)); plot(cs1n); layout(1)
```

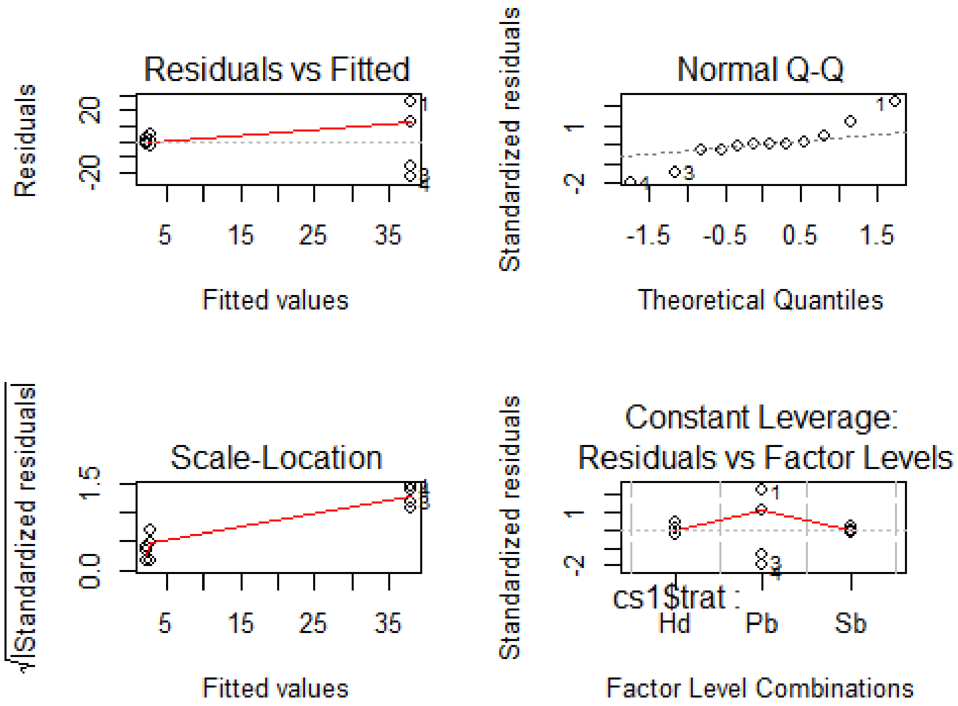


```
shapiro.test(cs1n$res)

##
##  Shapiro-Wilk normality test
##
## data:  cs1n$res
## W = 0.91806, p-value = 0.2703

plot(pen ~ trat, data = cs1)
```

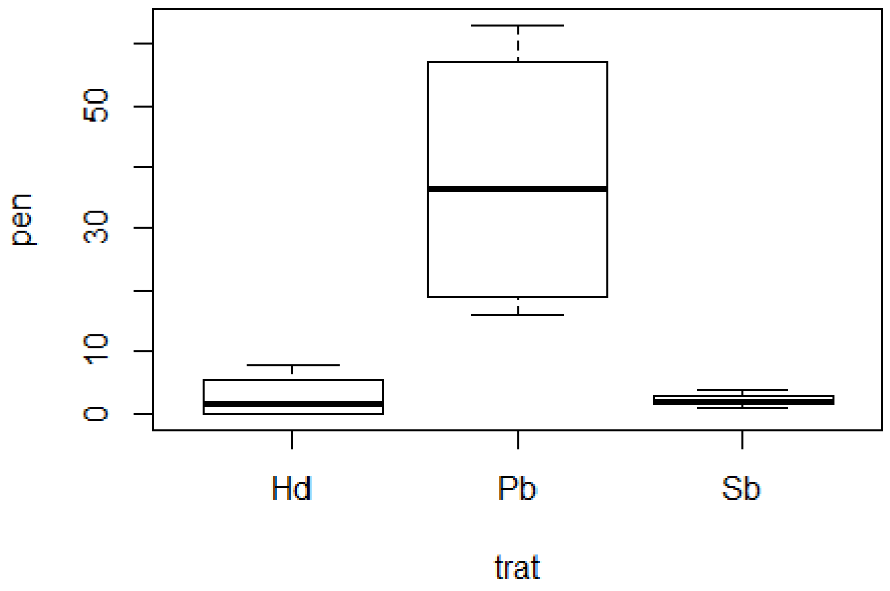


```
bartlett.test(cs1$pen, cs1$trat)

##
##  Bartlett test of homogeneity of variances
##
## data:  cs1$pen and cs1$trat
## Bartlett's K-squared = 16.078, df = 2, p-value = 0.0003226

# Transforma??o Box-Cox
boxcox(pen+0.01 ~ trat, data=cs1, plotit=T)
```

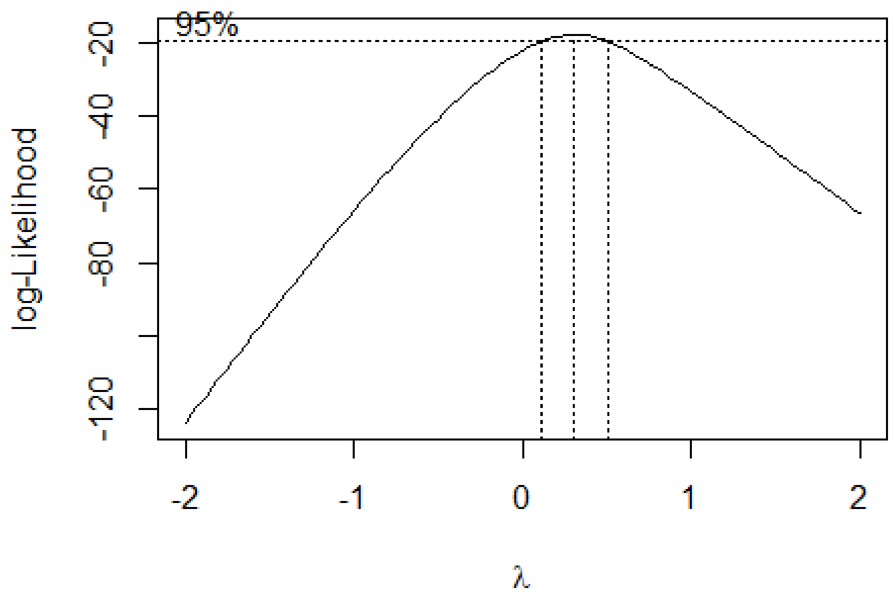


```
bc <- boxcox(pen+0.01 ~ trat, data=cs1, lam=seq(-.5, 1, 1/10))
```

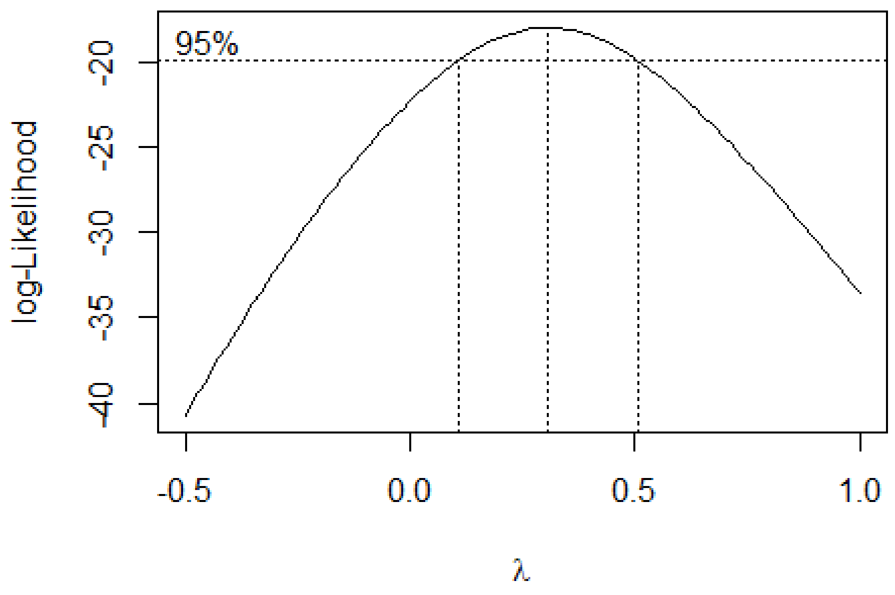


```
(lambda = bc$x[which.max(bc$y)])
```

```
## [1] 0.3030303
```

```
n1<-log(cs1$pen+0.01)
```

```
cs1n1<-aov(n1~cs1$trat)
cs1n1
```

```
## Call:
##    aov(formula = n1 ~ cs1$trat)
##
## Terms:
##                  cs1$trat Residuals
## Sum of Squares   50.06352  41.12068
## Deg. of Freedom         2         9
##
## Residual standard error: 2.137514
## Estimated effects may be unbalanced
```

```
summary(cs1n1)
```

```
##             Df Sum Sq Mean Sq F value Pr(>F)
## cs1$trat     2  50.06  25.032   5.479 0.0278 *
## Residuals    9  41.12   4.569
## ---
## Signif. codes:  0 '***' 0.001 '**' 0.01 '*' 0.05 '.' 0.1 ' ' 1
```

```
par(mfrow=c(2,2)); plot(cs1n1); layout(1)
```

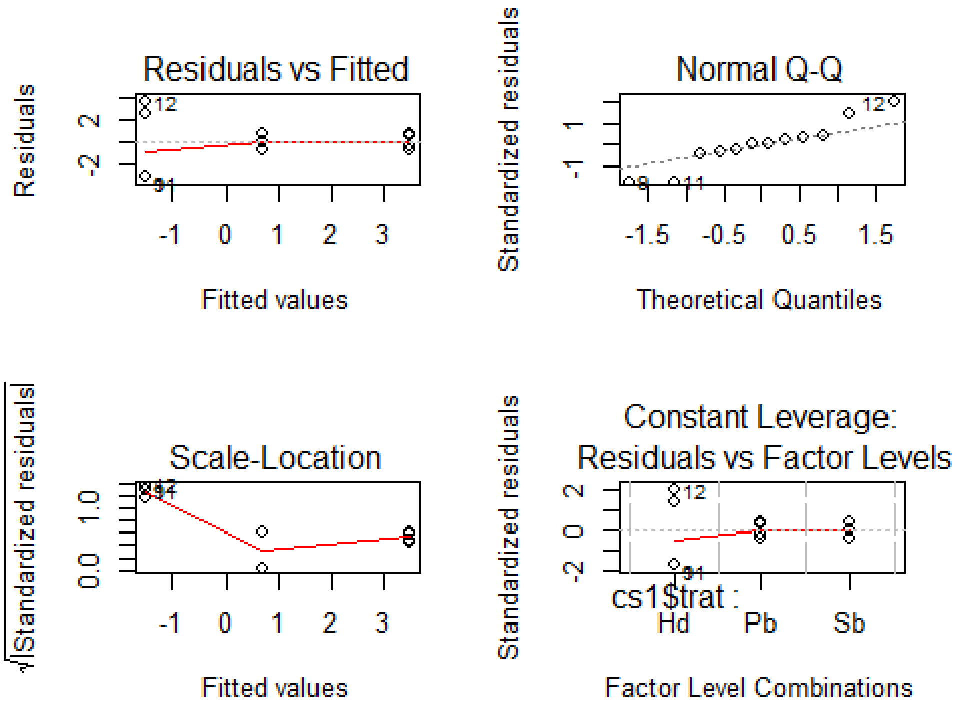


```
shapiro.test(cs1n1$res)
```

```
##
##  Shapiro-Wilk normality test
##
## data:  cs1n1$res
## W = 0.92836, p-value = 0.3631
```

```
plot(n1 ~ trat, data = cs1)
```

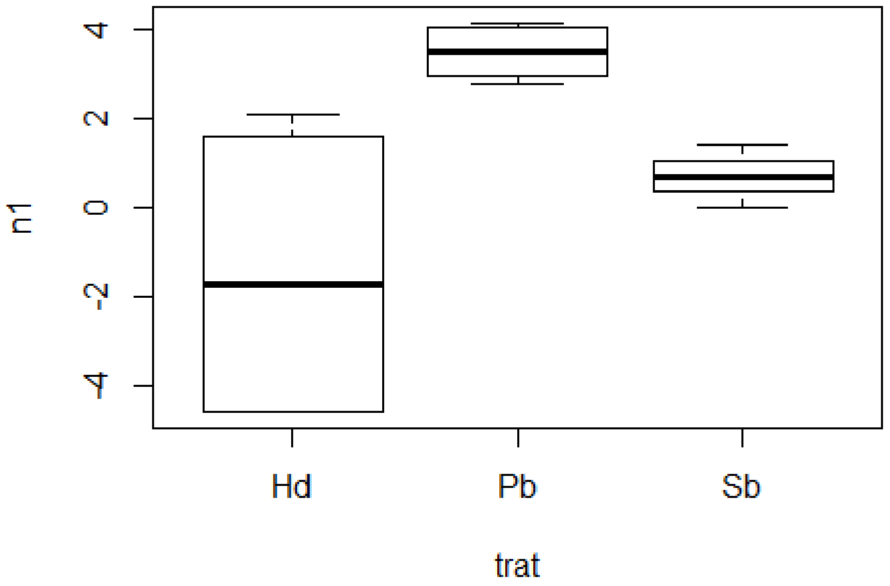


```
bartlett.test(n1, cs1$trat)
```

```
##
##  Bartlett test of homogeneity of variances
##
## data:  n1 and cs1$trat
## Bartlett's K-squared = 10.414, df = 2, p-value = 0.005477
```

```
require(agricolae)
glr <- df.residual(cs1n1)
glr
```

```
## [1] 9
```

```
sqr <- deviance(cs1n1)
sqr
```

```
## [1] 41.12068
```

```
qmr <- sqr/glr
qmr
```

```
## [1] 4.568964
```

```
lsdn <- LSD.test(n1,cs1$trat, glr, qmr, alpha=0.05, p.adj="none")
lsdn
```

```
## $statistics
##    MSerror Df      Mean       CV  t.value      LSD
##   4.568964  9 0.8922773 239.5571 2.262157 3.419138
##
## $parameters
##         test p.ajusted   name.t ntr alpha
##   Fisher-LSD      none cs1$trat   3  0.05
##
## $means
##            n1       std r       LCL       UCL          Min      Max
## Hd -1.5069274 3.5997875 4 -3.924623 0.9107684 -4.605170186 2.080691
## Pb  3.4850064 0.6569280 4  1.067311 5.9027021  2.773213527 4.143293
## Sb  0.6987528 0.5629099 4 -1.718943 3.1164485  0.009950331 1.388791
##           Q25        Q50       Q75
## Hd -4.6051702 -1.7516151 1.3466277
## Pb  3.0119261  3.5117593 3.9848396
## Sb  0.5260886  0.6981347 0.8707989
##
## $comparison
## NULL
##
## $groups
##            n1 groups
## Pb  3.4850064      a
## Sb  0.6987528     ab
## Hd -1.5069274      b
##
## attr(,"class")
## [1] "group"
```

```
par(mfrow=c(1,1))
pot.m <- with(cs1, tapply(pen, trat, mean))
pot.m
```

```
##    Hd    Pb    Sb
##  2.75 38.00  2.25
```

```
bp <- barplot(pot.m, ylim=c(0,50))
text(bp, pot.m, label=round(pot.m, 3), pos=3)
title("27 DAI")
box()
```

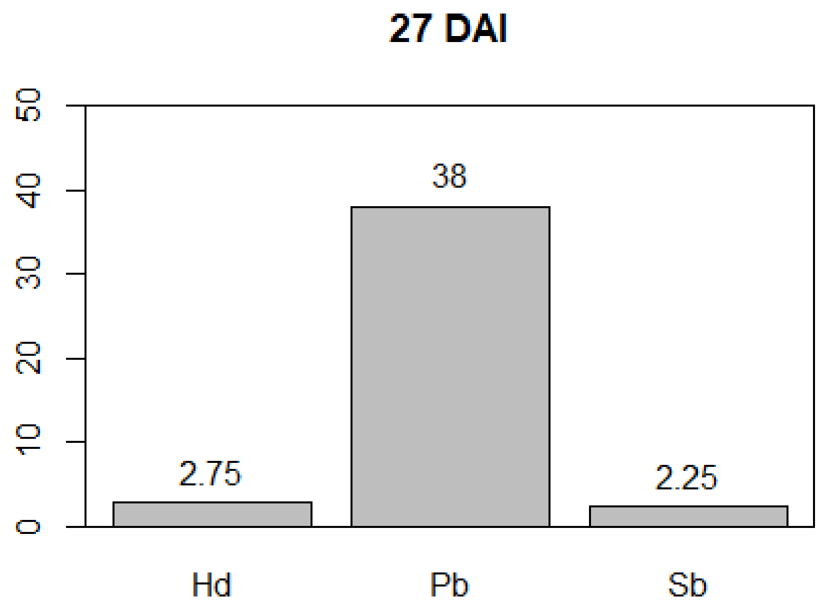

27 DAI
0
10
20
30
40
50
2.75
38
2.25
Hd
Pb
Sb
