# Supplementary material for: Two novel potential pathogens for soybean
Source: PLoS One. 2019 Aug 22;14(8):e0221416. doi: 10.1371/journal.pone.0221416 (PMC6705753; doi:10.1371/journal.pone.0221416)
Supplement: S4 File — (PDF) [file pone.0221416.s004.pdf]

27_DAI_experiment_2.R

Santino

Tue Jul 23 18:59:02 2019

```
rm(list = ls())
cs1<-read.table("C:\\analises nemato\\soja comparativo analises\\soja
comp 27 esq.txt",h=T,dec=",")
cs1
```

```
##    trat baer pen
## 1    Pb  102  72
## 2    Pb  122  22
## 3    Pb  216  65
## 4    Pb  178  55
## 5    Sb   28  42
## 6    Sb   26  27
## 7    Sb   31  29
## 8    Sb   21  52
## 9    Hd   21  55
## 10   Hd   36  13
## 11   Hd   41  19
## 12   Hd   30   7
```

```
data.frame(table(cs1$trat))
```

```
##   Var1 Freq
## 1   Hd    4
## 2   Pb    4
## 3   Sb    4
```

```
attach(cs1)
# mean and median
```

```
(Medias = with(cs1 [3], aggregate(. ~trat, data=cs1[ 3], mean)))
```

```
##   trat  pen
## 1   Hd 23.5
## 2   Pb 53.5
## 3   Sb 37.5
```

```
(Medianas = with(cs1 [3], aggregate(. ~trat, data=cs1[ 3], median)))
```

```
##   trat  pen
## 1   Hd 16.0
## 2   Pb 60.0
## 3   Sb 35.5
```

```
#standard deviation
sd(cs1$pen)

## [1] 21.48925

#variation coef
cv(cs1$pen, na.rm=TRUE)

## [1] 56.30371

#nematodes in roots


cs1n<-aov(cs1$pen~cs1$trat)
cs1n

## Call:
##    aov(formula = cs1$pen ~ cs1$trat)
##
## Terms:
##                  cs1$trat Residuals
## Sum of Squares   1802.667  3277.000
## Deg. of Freedom         2         9
##
## Residual standard error: 19.0817
## Estimated effects may be unbalanced

summary(cs1n)

##             Df Sum Sq Mean Sq F value Pr(>F)
## cs1$trat     2   1803   901.3   2.475  0.139
## Residuals    9   3277   364.1

par(mfrow=c(2,2)); plot(cs1n); layout(1)
```

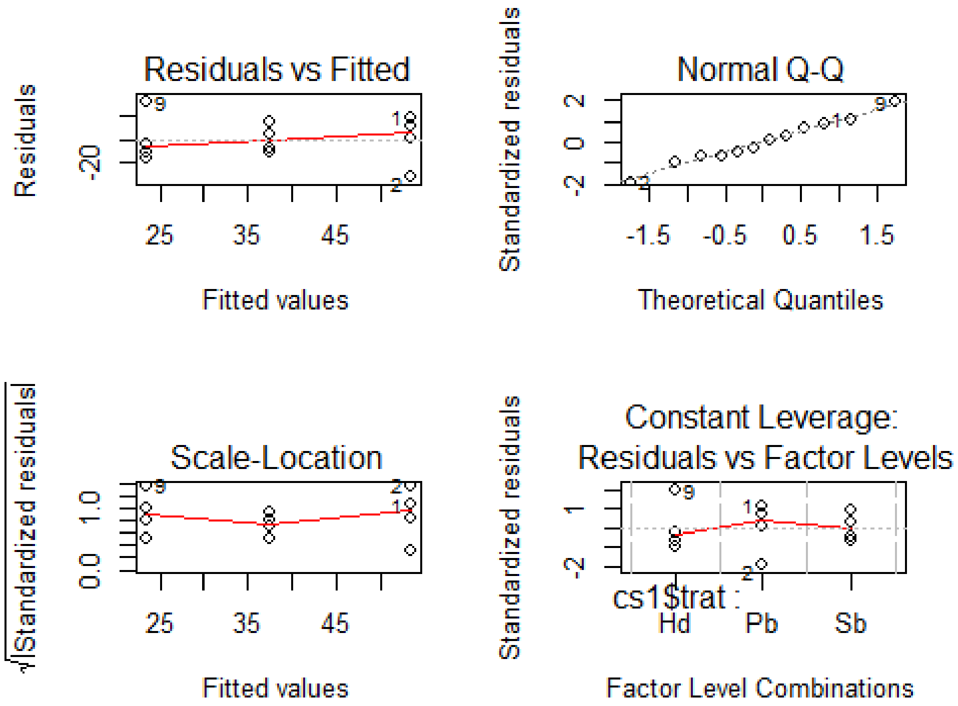


```r
shapiro.test(cs1n$res)

##
##  Shapiro-Wilk normality test
##
## data:  cs1n$res
## W = 0.98696, p-value = 0.9985

plot(pen ~ trat, data = cs1)
```

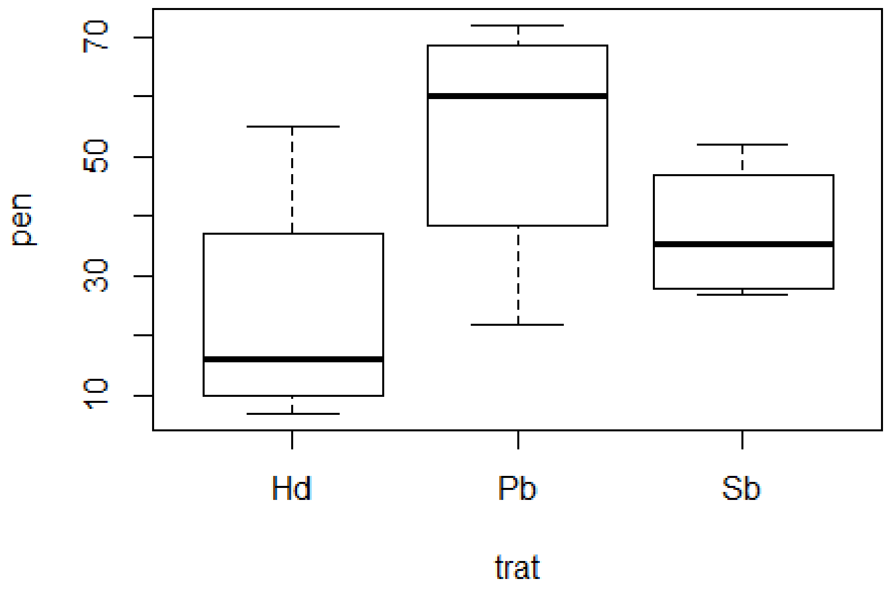


```
bartlett.test(cs1$pen, cs1$trat)

##
##  Bartlett test of homogeneity of variances
##
## data:  cs1$pen and cs1$trat
## Bartlett's K-squared = 1.1282, df = 2, p-value = 0.5689

require(agricolae)
glr <- df.residual(cs1n)
glr

## [1] 9

sqr <- deviance(cs1n)
sqr

## [1] 3277

qmr <- sqr/glr
qmr

## [1] 364.1111

lsdn <- LSD.test(cs1$pen,cs1$trat, glr, qmr, alpha=0.05, p.adj="none")
lsdn

## $statistics
##    MSerror Df     Mean       CV  t.value      LSD
```

```
##   364.1111  9 38.16667 49.99571 2.262157 30.52283
##
## $parameters
##         test p.ajusted   name.t ntr alpha
##   Fisher-LSD      none cs1$trat   3  0.05
##
## $means
##    cs1$pen      std r       LCL      UCL Min Max   Q25  Q50   Q75
## Hd    23.5 21.56386 4  1.917103 45.0829   7  55 11.50 16.0 28.00
## Pb    53.5 22.12841 4 31.917103 75.0829  22  72 46.75 60.0 66.75
## Sb    37.5 11.73314 4 15.917103 59.0829  27  52 28.50 35.5 44.50
##
## $comparison
## NULL
##
## $groups
##    cs1$pen groups
## Pb    53.5      a
## Sb    37.5      a
## Hd    23.5      a
##
## attr(,"class")
## [1] "group"
```

```
par(mfrow=c(1,1))
pot.m <- with(cs1, tapply(pen, trat, mean))
pot.m
```

```
##   Hd   Pb   Sb
## 23.5 53.5 37.5
```

```
bp <- barplot(pot.m, ylim=c(0,70))
text(bp, pot.m, label=round(pot.m, 3), pos=3)
title("27 DAI")
box()
```

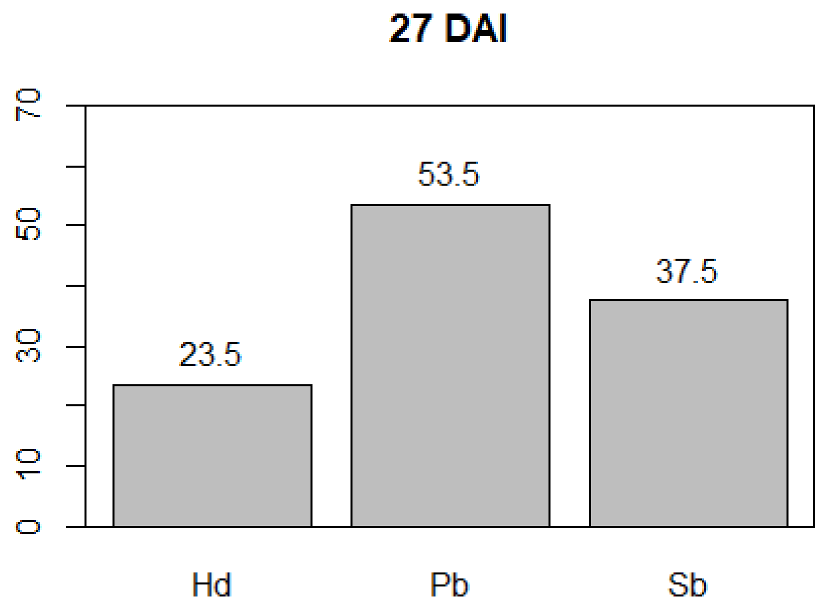
27 DAI
0
10
30
50
70
23.5
53.5
37.5
Hd
Pb
Sb
