# Supplementary material for: Two novel potential pathogens for soybean
Source: PLoS One. 2019 Aug 22;14(8):e0221416. doi: 10.1371/journal.pone.0221416 (PMC6705753; doi:10.1371/journal.pone.0221416)
Supplement: S5 File — (PDF) [file pone.0221416.s005.pdf]

70_dai_experiment_1.R

Santino

Tue Jul 23 18:53:01 2019

```
rm(list = ls())
cs1<-read.table("C:\\analises nemato\\soja comparativo analises\\Soja
comparativo emb p.txt",h=T,dec=",")
cs1
```

```
##         trat   mfr mfpa
## 1  POTENCIA 21.31 43.0
## 2  POTENCIA 23.14 44.0
## 3  POTENCIA 27.97 57.0
## 4  POTENCIA 19.94 35.0
## 5  POTENCIA 24.14 37.0
## 6  POTENCIA 14.20 37.0
## 7  POTENCIA 13.55 39.0
## 8  POTENCIA 13.90 34.0
## 9        A8 11.42 32.0
## 10       A8 13.21 26.0
## 11       A8 12.40 31.5
## 12       A8 10.93 25.0
## 13       A8 11.58 14.0
## 14       A8 10.43 45.0
## 15       A8  6.24 18.0
## 16       A8 16.43 26.0
## 17      A13 11.76 32.0
## 18      A13 16.62 38.0
## 19      A13  9.10 26.0
## 20      A13  8.27 33.0
## 21      A13 12.43 32.5
## 22      A13  9.28 29.0
## 23      A13  8.05 22.5
## 24      A13 15.99 35.0
## 25      A15  8.63 23.5
## 26      A15 11.75 32.0
## 27      A15 10.61 23.0
## 28      A15 18.12 30.0
## 29      A15 13.61 32.5
## 30      A15  5.76 15.5
## 31      A15  6.45  7.5
## 32      A15  6.84 16.5
```

```
data.frame(table(cs1$trat))
```

```
##       Var1 Freq
## 1      A13    8
```

```
## 2      A15    8
## 3       A8    8
## 4 POTENCIA    8
```

```r
require(graphics)
require(ExpDes)
```

```
## Loading required package: ExpDes
```

```r
require(MASS)
```

```r
require(agricolae)
```

```r
attach(cs1)
```

```r
# mean and median
```

```r
(Medias = with(cs1 [, 2:3], aggregate(. ~trat, data=cs1[ ,2:3], mean)))
```

```
##       trat      mfr    mfpa
## 1      A13 11.43750 31.0000
## 2      A15 10.22125 22.5625
## 3       A8 11.58000 27.1875
## 4 POTENCIA 19.76875 40.7500
```

```r
(Medianas = with(cs1 [, 2:3], aggregate(. ~trat, data=cs1[ ,2:3],
median)))
```

```
##       trat    mfr  mfpa
## 1      A13 10.520 32.25
## 2      A15  9.620 23.25
## 3       A8 11.500 26.00
## 4 POTENCIA 20.625 38.00
```

```r
#standard deviation
sd(cs1$mfr)
```

```
## [1] 5.471586
```

```r
sd(cs1$mfpa)
```

```
## [1] 10.12741
```

```r
#variation coef
require(raster)
```

```r
cv(cs1$mfr, na.rm=TRUE)
```

```
## [1] 41.28915
```

```r
cv(cs1$mfpa, na.rm=TRUE)
```

```
## [1] 33.34128
```

```
#fresh top weigth
cs1mfp<-aov(cs1$mfpa~cs1$trat)
cs1mfp

## Call:
##    aov(formula = cs1$mfpa ~ cs1$trat)
##
## Terms:
##                 cs1$trat Residuals
## Sum of Squares  1433.812  1745.688
## Deg. of Freedom        3        28
##
## Residual standard error: 7.895947
## Estimated effects may be unbalanced

summary(cs1mfp)

##             Df Sum Sq Mean Sq F value   Pr(>F)
## cs1$trat     3   1434   477.9   7.666 0.000684 ***
## Residuals   28   1746    62.3
## ---
## Signif. codes:  0 '***' 0.001 '**' 0.01 '*' 0.05 '.' 0.1 ' ' 1

par(mfrow=c(2,2)); plot(cs1mfp); layout(1)
```

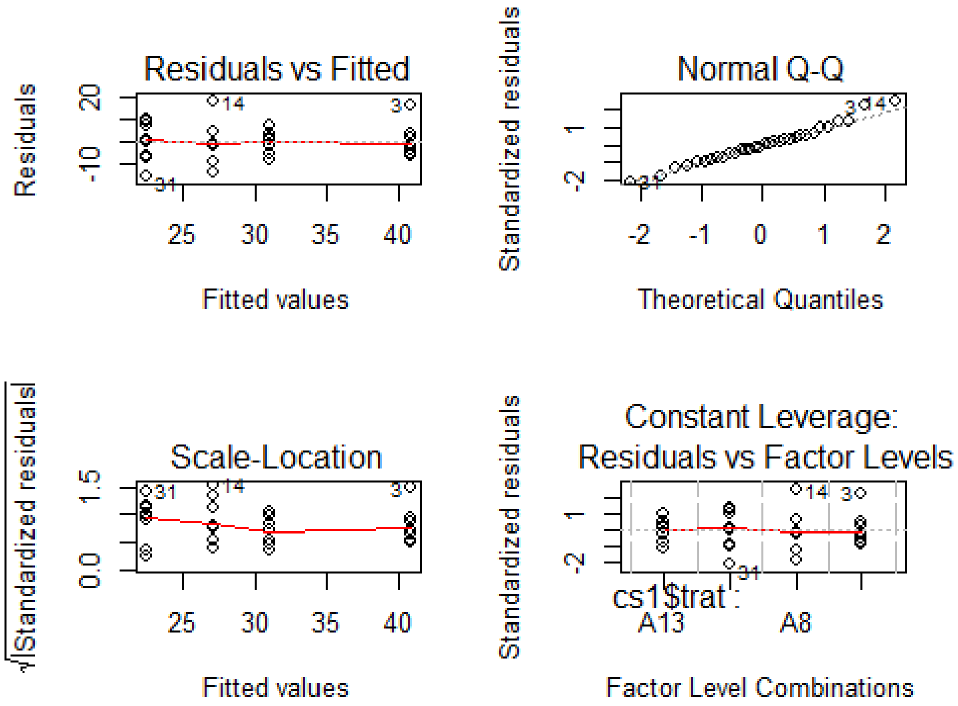


```
shapiro.test(cs1mfp$res)

##
##  Shapiro-Wilk normality test
```

```
##
## data:  cs1mfp$res
## W = 0.98245, p-value = 0.8668
```

```
require(agricolae)
glr <- df.residual(cs1mfp)
glr
```

```
## [1] 28
```

```
sqr <- deviance(cs1mfp)
sqr
```

```
## [1] 1745.688
```

```
qmr <- sqr/glr
qmr
```

```
## [1] 62.34598
```

```
lsdpa <- LSD.test(cs1$mfpa,cs1$trat, glr, qmr, alpha=0.05, p.adj="none")
lsdpa
```

```
## $statistics
##    MSerror Df   Mean       CV  t.value      LSD
##   62.34598 28 30.375 25.99489 2.048407 8.087057
##
## $parameters
##         test p.ajusted   name.t ntr alpha
##   Fisher-LSD      none cs1$trat   4  0.05
##
## $means
##           cs1$mfpa      std r      LCL      UCL  Min  Max   Q25   Q50
## A13        31.0000 4.978525 8 25.28159 36.71841 22.5 38.0 28.25 32.25
## A15        22.5625 8.926035 8 16.84409 28.28091  7.5 32.5 16.25 23.25
## A8         27.1875 9.448876 8 21.46909 32.90591 14.0 45.0 23.25 26.00
## POTENCIA   40.7500 7.459414 8 35.03159 46.46841 34.0 57.0 36.50 38.00
##              Q75
## A13       33.500
## A15       30.500
## A8        31.625
## POTENCIA  43.250
##
## $comparison
## NULL
##
## $groups
##           cs1$mfpa groups
## POTENCIA   40.7500      a
## A13        31.0000      b
## A8         27.1875     bc
## A15        22.5625      c
```

```
##
## attr(,"class")
## [1] "group"
```

```r
#fresh root weigth

cs1mfr<-aov(cs1$mfr~cs1$trat)
cs1mfr
```

```
## Call:
##    aov(formula = cs1$mfr ~ cs1$trat)
##
## Terms:
##                 cs1$trat Residuals
## Sum of Squares  461.9318  466.1541
## Deg. of Freedom        3        28
##
## Residual standard error: 4.08024
## Estimated effects may be unbalanced
```

```r
summary(cs1mfr)
```

```
##             Df Sum Sq Mean Sq F value   Pr(>F)
## cs1$trat     3  461.9  153.98   9.249 0.000205 ***
## Residuals   28  466.2   16.65
## ---
## Signif. codes:  0 '***' 0.001 '**' 0.01 '*' 0.05 '.' 0.1 ' ' 1
```

```r
par(mfrow=c(2,2)); plot(cs1mfr); layout(1)
```

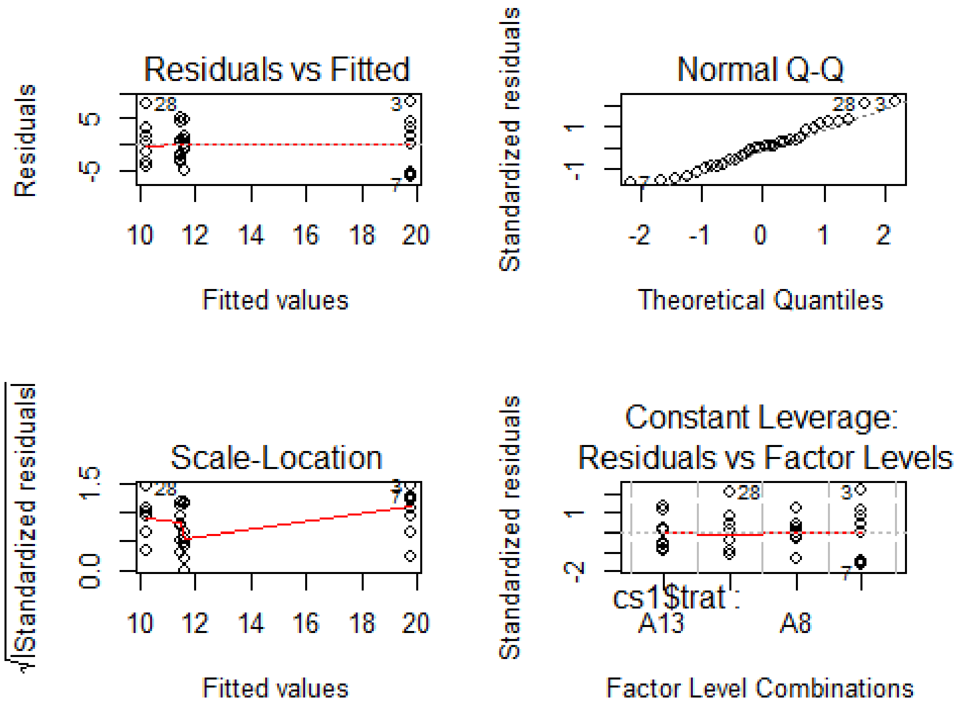


```
shapiro.test(cs1mfr$res)

##
##  Shapiro-Wilk normality test
##
## data:  cs1mfr$res
## W = 0.96964, p-value = 0.4896

require(agricolae)
glr <- df.residual(cs1mfr)
glr

## [1] 28

sqr <- deviance(cs1mfr)
sqr

## [1] 466.1541

qmr <- sqr/glr
qmr

## [1] 16.64836

lsdr <- LSD.test(cs1$mfr,cs1$trat, glr, qmr, alpha=0.05, p.adj="none")
lsdr

## $statistics
##    MSerror Df      Mean        CV  t.value      LSD
```

```
##   16.64836 28 13.25188 30.78991 2.048407 4.178997
## 
## $parameters
##         test p.ajusted   name.t ntr alpha
##   Fisher-LSD      none cs1$trat   4  0.05
## 
## $means
##            cs1$mfr      std r       LCL      UCL   Min   Max     Q25
Q50
## A13      11.43750 3.386940 8  8.482503 14.39250  8.05 16.62  8.8925
10.520
## A15      10.22125 4.216500 8  7.266253 13.17625  5.76 18.12  6.7425
9.620
## A8       11.58000 2.857021 8  8.625003 14.53500  6.24 16.43 10.8050
11.500
## POTENCIA 19.76875 5.401911 8 16.813753 22.72375 13.55 27.97 14.1250
20.625
##              Q75
## A13      13.3200
## A15      12.2150
## A8       12.6025
## POTENCIA 23.3900
## 
## $comparison
## NULL
## 
## $groups
##            cs1$mfr groups
## POTENCIA 19.76875      a
## A8       11.58000      b
## A13      11.43750      b
## A15      10.22125      b
## 
## attr(,"class")
## [1] "group"
```
