# Supplementary material for: Two novel potential pathogens for soybean
Source: PLoS One. 2019 Aug 22;14(8):e0221416. doi: 10.1371/journal.pone.0221416 (PMC6705753; doi:10.1371/journal.pone.0221416)
Supplement: S6 File — (PDF) [file pone.0221416.s006.pdf]

70_dai_experiment_2.R

Santino

Tue Jul 23 19:12:33 2019

```
rm(list = ls())
cs1<-read.table("C:\\analises nemato\\soja comparativo analises\\Soja
comparativo esq p.txt",h=T,dec=",")
cs1
```

```
##        trat   mfr mfpa
## 1  POTENCIA 31.11 43.0
## 2  POTENCIA 30.10 44.0
## 3  POTENCIA 32.60 57.0
## 4  POTENCIA 32.14 47.0
## 5  POTENCIA 23.90 39.0
## 6  POTENCIA 31.12 46.0
## 7  POTENCIA 33.10 39.0
## 8        A8 39.53 44.0
## 9        A8 22.80 41.5
## 10       A8 20.31 33.5
## 11       A8 24.63 53.5
## 12       A8 26.12 40.0
## 13       A8 34.72 49.0
## 14       A8 37.67 49.0
## 15      A13 26.79 65.0
## 16      A13 15.21 52.0
## 17      A13 33.00 43.0
## 18      A13 43.52 69.5
## 19      A13 38.92 62.5
## 20      A13 32.37 50.5
## 21      A13 42.72 56.0
## 22      A15 34.58 48.0
## 23      A15 41.62 64.5
## 24      A15 39.30 53.0
## 25      A15 26.42 66.5
## 26      A15 38.04 52.0
## 27      A15 32.67 58.5
## 28      A15 32.92 57.0
```

```
data.frame(table(cs1$trat))
```

```
##       Var1 Freq
## 1      A13    7
## 2      A15    7
## 3       A8    7
## 4 POTENCIA    7
```

```r
require(graphics)
require(ExpDes)

require(MASS)

require(agricolae)

attach(cs1)

# mean and median

(Medias = with(cs1 [, 2:3], aggregate(. ~trat, data=cs1[ ,2:3], mean)))

##       trat      mfr     mfpa
## 1      A13 33.21857 56.92857
## 2      A15 35.07857 57.07143
## 3       A8 29.39714 44.35714
## 4 POTENCIA 30.58143 45.00000

(Medianas = with(cs1 [, 2:3], aggregate(. ~trat, data=cs1[ ,2:3],
median)))

##       trat   mfr mfpa
## 1      A13 33.00   56
## 2      A15 34.58   57
## 3       A8 26.12   44
## 4 POTENCIA 31.12   44

#standard deviation
sd(cs1$mfr)

## [1] 6.954303

sd(cs1$mfpa)

## [1] 9.324454

#variation coef
require(raster)

cv(cs1$mfr, na.rm=TRUE)

## [1] 21.68549

cv(cs1$mfpa, na.rm=TRUE)

## [1] 18.34104

#fresh top weigth

cs1mfp<-aov(cs1$mfpa~cs1$trat)
cs1mfp

## Call:
##    aov(formula = cs1$mfpa ~ cs1$trat)
##
```

```
## Terms:
##                  cs1$trat Residuals
## Sum of Squares  1064.241  1283.286
## Deg. of Freedom        3        24
##
## Residual standard error: 7.312335
## Estimated effects may be unbalanced
```

```
summary(cs1mfp)
```

```
##             Df Sum Sq Mean Sq F value  Pr(>F)
## cs1$trat     3   1064   354.7   6.634 0.00202 **
## Residuals   24   1283    53.5
## ---
## Signif. codes:  0 '***' 0.001 '**' 0.01 '*' 0.05 '.' 0.1 ' ' 1
```

```
par(mfrow=c(2,2)); plot(cs1mfp); layout(1)
```

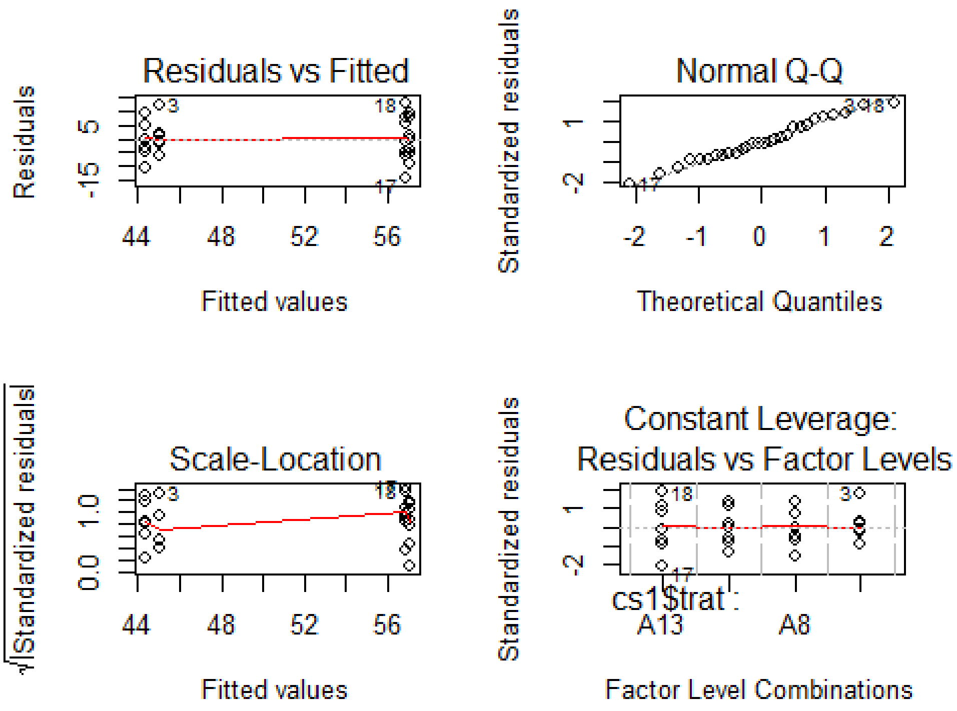


```
shapiro.test(cs1mfp$res)
```

```
##
##  Shapiro-Wilk normality test
##
## data:  cs1mfp$res
## W = 0.97821, p-value = 0.8054
```

```
require(agricolae)
glr <- df.residual(cs1mfp)
glr
```

```
## [1] 24

sqr <- deviance(cs1mfp)
sqr

## [1] 1283.286

qmr <- sqr/glr
qmr

## [1] 53.47024

lsdpa <- LSD.test(cs1$mfpa,cs1$trat, glr, qmr, alpha=0.05, p.adj="none")
lsdpa

## $statistics
##    MSerror Df     Mean       CV  t.value      LSD
##   53.47024 24 50.83929 14.38324 2.063899 8.066969
##
## $parameters
##         test p.ajusted   name.t ntr alpha
##   Fisher-LSD      none cs1$trat   4  0.05
##
## $means
##           cs1$mfpa      std r      LCL      UCL  Min  Max   Q25 Q50
Q75
## A13       56.92857 9.262058 7 51.22436 62.63278 43.0 69.5 51.25  56
63.75
## A15       57.07143 6.717071 7 51.36722 62.77564 48.0 66.5 52.50  57
61.50
## A8        44.35714 6.731235 7 38.65293 50.06135 33.5 53.5 40.75  44
49.00
## POTENCIA 45.00000 6.137318 7 39.29579 50.70421 39.0 57.0 41.00  44
46.50
##
## $comparison
## NULL
##
## $groups
##           cs1$mfpa groups
## A15       57.07143      a
## A13       56.92857      a
## POTENCIA 45.00000      b
## A8        44.35714      b
##
## attr(,"class")
## [1] "group"

#fresh roots weigth

cs1mfr<-aov(cs1$mfr~cs1$trat)
cs1mfr
```

```
## Call:
##    aov(formula = cs1$mfr ~ cs1$trat)
##
## Terms:
##                 cs1$trat Residuals
## Sum of Squares  138.1151 1167.6680
## Deg. of Freedom        3        24
##
## Residual standard error: 6.975158
## Estimated effects may be unbalanced
```

```
summary(cs1mfr)
```

```
##             Df Sum Sq Mean Sq F value Pr(>F)
## cs1$trat     3  138.1   46.04   0.946  0.434
## Residuals   24 1167.7   48.65
```

```
par(mfrow=c(2,2)); plot(cs1mfr); layout(1)
```

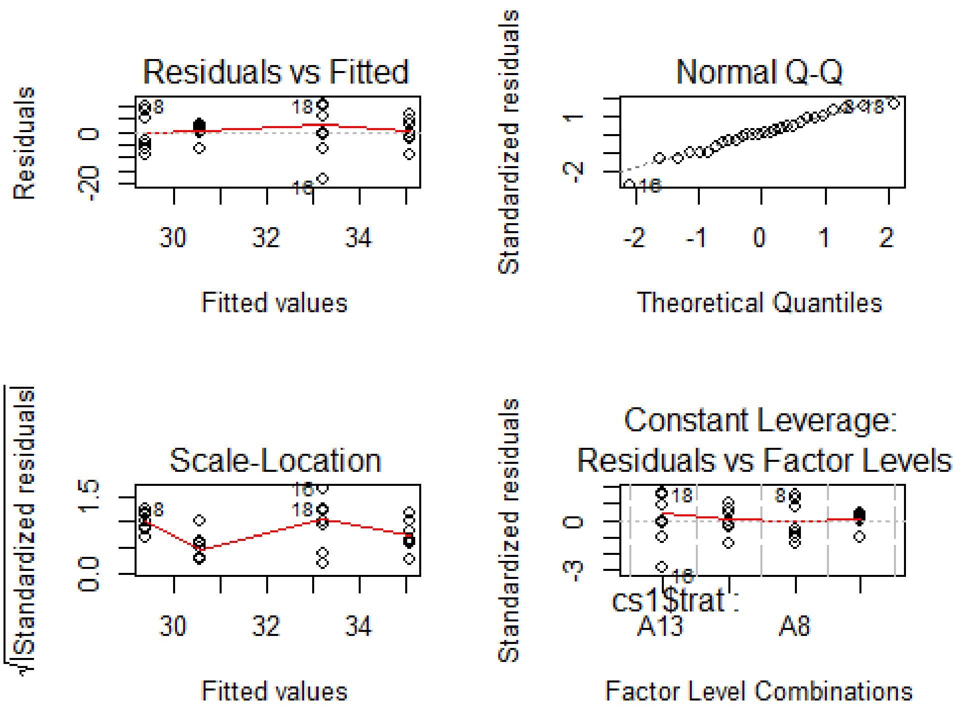


```
shapiro.test(cs1mfr$res)
```

```
##
##  Shapiro-Wilk normality test
##
## data:  cs1mfr$res
## W = 0.96625, p-value = 0.4844
```

```r
require(agricolae)
glr <- df.residual(cs1mfr)
glr
```

```
## [1] 24
```

```r
sqr <- deviance(cs1mfr)
sqr
```

```
## [1] 1167.668
```

```r
qmr <- sqr/glr
qmr
```

```
## [1] 48.65283
```

```r
lsdr <- LSD.test(cs1$mfr,cs1$trat, glr, qmr, alpha=0.05, p.adj="none")
lsdr
```

```
## $statistics
##    MSerror Df     Mean       CV  t.value      LSD
##   48.65283 24 32.06893 21.75052 2.063899 7.694996
##
## $parameters
##         test p.ajusted   name.t ntr alpha
##   Fisher-LSD      none cs1$trat   4  0.05
##
## $means
##            cs1$mfr      std r      LCL      UCL   Min   Max    Q25
Q50
## A13       33.21857 9.960806 7 27.77739 38.65976 15.21 43.52 29.580
33.00
## A15       35.07857 5.083648 7 29.63739 40.51976 26.42 41.62 32.795
34.58
## A8        29.39714 7.735325 7 23.95596 34.83833 20.31 39.53 23.715
26.12
## POTENCIA 30.58143 3.116881 7 25.14024 36.02261 23.90 33.10 30.605
31.12
##              Q75
## A13       40.820
## A15       38.670
## A8        36.195
## POTENCIA 32.370
##
## $comparison
## NULL
##
## $groups
##            cs1$mfr groups
## A15       35.07857      a
## A13       33.21857      a
## POTENCIA 30.58143      a
```

```
## A8        29.39714       a
##
## attr(,"class")
## [1] "group"
```
