# Supplementary material for: Two novel potential pathogens for soybean
Source: PLoS One. 2019 Aug 22;14(8):e0221416. doi: 10.1371/journal.pone.0221416 (PMC6705753; doi:10.1371/journal.pone.0221416)
Supplement: S7 File — (PDF) [file pone.0221416.s007.pdf]

70_dai_experiment_1_nematode.R

Santino

Tue Jul 23 19:18:39 2019

```
rm(list = ls())
cs1<-read.table("C:\\analises nemato\\soja comparativo analises\\Soja
comparativo emb n.txt",h=T,dec=",")
cs1
```

```
##    trat raiz solo  tot    fr nema
## 1    A8  720    8  728 0.728   63
## 2    A8  616    4  620 0.620   47
## 3    A8  280    3  283 0.283   23
## 4    A8  704   12  716 0.716   64
## 5    A8  798    5  803 0.803   69
## 6    A8  320    6  326 0.326   31
## 7    A8  380    8  388 0.388   61
## 8    A8 1144   40 1184 1.184   70
## 9   A13  800  161  961 0.961   68
## 10  A13  420  357  777 0.777   25
## 11  A13  510  253  763 0.763   56
## 12  A13  816  270 1086 1.086   99
## 13  A13  816  111  927 0.927   66
## 14  A13 1148  646 1794 1.794  124
## 15  A13 1530  207 1737 1.737  190
## 16  A13  506  763 1269 1.269   32
## 17  A15  384 1711 2095 2.095   44
## 18  A15  308 3048 3356 3.356   26
## 19  A15  266 1152 1418 1.418   25
## 20  A15  612 2160 2772 2.772   34
## 21  A15  160  480  640 0.640   12
## 22  A15  306  393  699 0.699   53
## 23  A15  168  904 1072 1.072   26
## 24  A15  102 6480 6582 6.582   15
```

```
data.frame(table(cs1$trat))
```

```
##   Var1 Freq
## 1  A13    8
## 2  A15    8
## 3   A8    8
```

```
require(graphics)
require(ExpDes)

require(MASS)

require(agricolae)
```

```r
attach(cs1)

# mean and median

(Medias = with(cs1 [, 2:6], aggregate(. ~trat, data=cs1[ ,2:6], mean)))

##   trat   raiz    solo     tot      fr   nema
## 1  A13 818.25  346.00 1164.25 1.16425 82.500
## 2  A15 288.25 2041.00 2329.25 2.32925 29.375
## 3   A8 620.25   10.75  631.00 0.63100 53.500

(Medianas = with(cs1 [, 2:6], aggregate(. ~trat, data=cs1[ ,2:6],
median)))

##   trat raiz   solo    tot     fr nema
## 1  A13  808  261.5 1023.5 1.0235   67
## 2  A15  286 1431.5 1756.5 1.7565   26
## 3   A8  660    7.0  668.0 0.6680   62

#standard deviation
sd(cs1$raiz)

## [1] 353.9614

sd(cs1$solo)

## [1] 1435.856

sd(cs1$tot)

## [1] 1337.702

sd(cs1$fr)

## [1] 1.337702

sd(cs1$nema)

## [1] 39.28969

#variation coef
require(raster)

cv(cs1$raiz, na.rm=TRUE)

## [1] 61.49612

cv(cs1$solo, na.rm=TRUE)

## [1] 179.6504

cv(cs1$tot, na.rm=TRUE)

## [1] 97.29925

cv(cs1$fr, na.rm=TRUE)
```

```
## [1] 97.29925

cv(cs1$nema, na.rm=TRUE)

## [1] 71.2738

#nematodes in roots

cr<-aov(cs1$raiz~cs1$trat)
cr

## Call:
##    aov(formula = cs1$raiz ~ cs1$trat)
##
## Terms:
##                 cs1$trat Residuals
## Sum of Squares   1147541   1734099
## Deg. of Freedom        2        21
##
## Residual standard error: 287.3606
## Estimated effects may be unbalanced

summary(cr)

##             Df  Sum Sq Mean Sq F value  Pr(>F)
## cs1$trat     2 1147541  573771   6.948 0.00483 **
## Residuals   21 1734099   82576
## ---
## Signif. codes:  0 '***' 0.001 '**' 0.01 '*' 0.05 '.' 0.1 ' ' 1

par(mfrow=c(2,2)); plot(cr); layout(1)
```

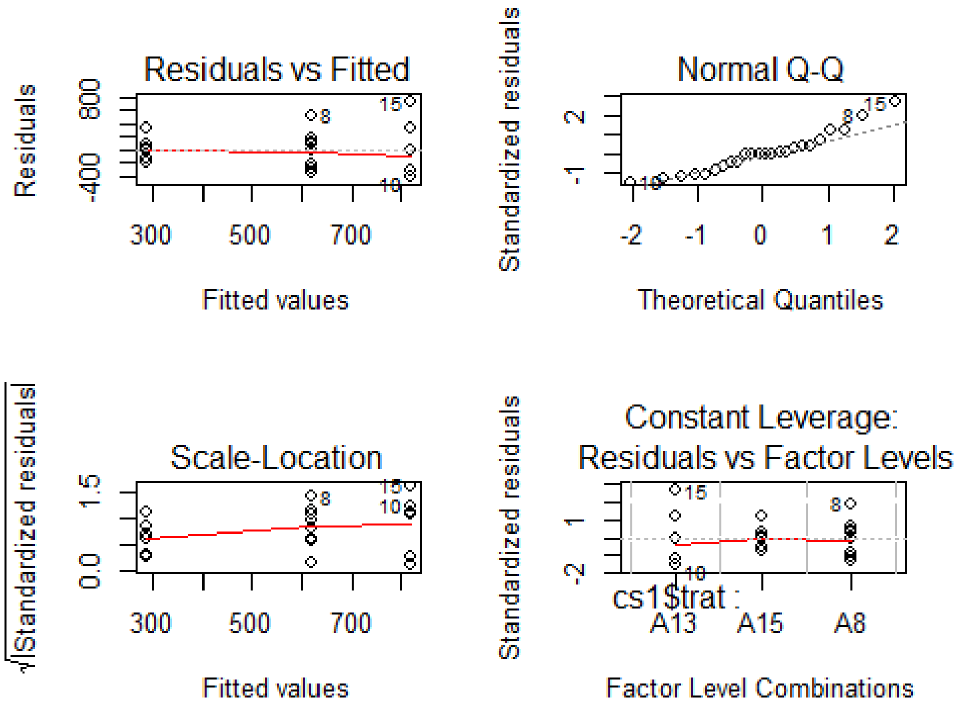


```
shapiro.test(cr$res)

##
##  Shapiro-Wilk normality test
##
## data:  cr$res
## W = 0.93554, p-value = 0.1297

# Transforma Box-Cox
boxcox(raiz ~ trat, data=cs1, plotit=T)
```

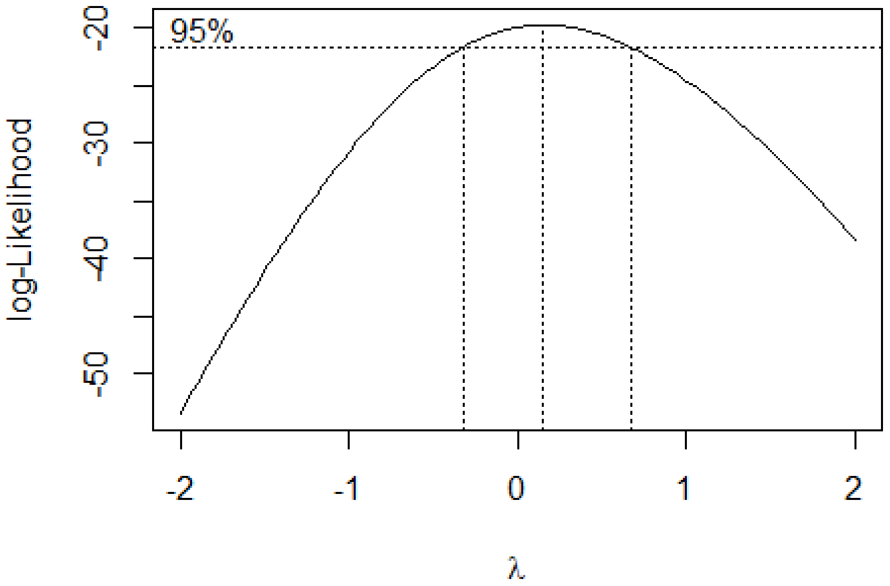


```
bc <- boxcox(raiz ~ trat, data=cs1, lam=seq(-.5, .5, 1/10))
```

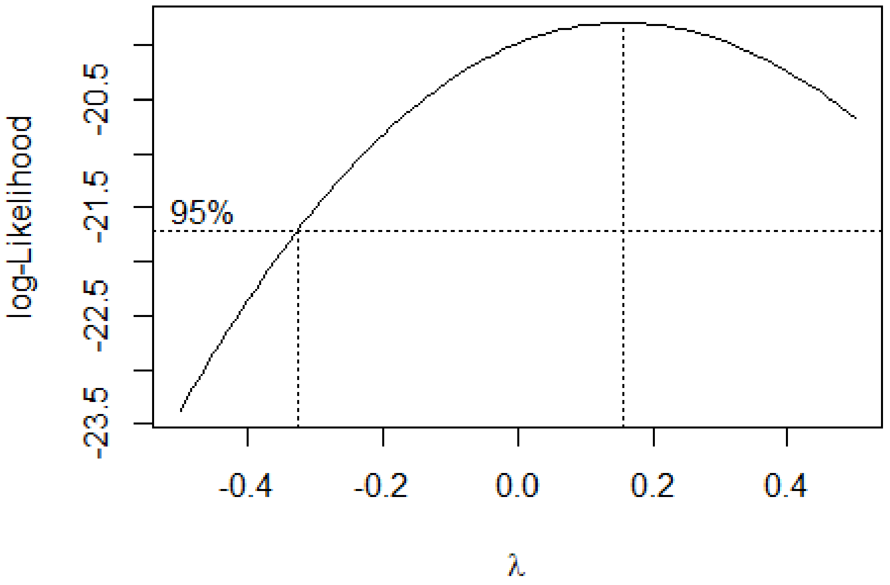


```
(lambda = bc$x[which.max(bc$y)])
```

```
## [1] 0.1565657

rl<-log(cs1$raiz+0.01)

cr1<-aov(rl~cs1$trat)
cr1

## Call:
##    aov(formula = rl ~ cs1$trat)
##
## Terms:
##                 cs1$trat Residuals
## Sum of Squares  5.121165  5.286113
## Deg. of Freedom        2        21
##
## Residual standard error: 0.5017167
## Estimated effects may be unbalanced

summary(cr1)

##             Df Sum Sq Mean Sq F value   Pr(>F)
## cs1$trat     2  5.121  2.5606   10.17 0.000815 ***
## Residuals   21  5.286  0.2517
## ---
## Signif. codes:  0 '***' 0.001 '**' 0.01 '*' 0.05 '.' 0.1 ' ' 1

par(mfrow=c(2,2)); plot(cr1); layout(1)
```

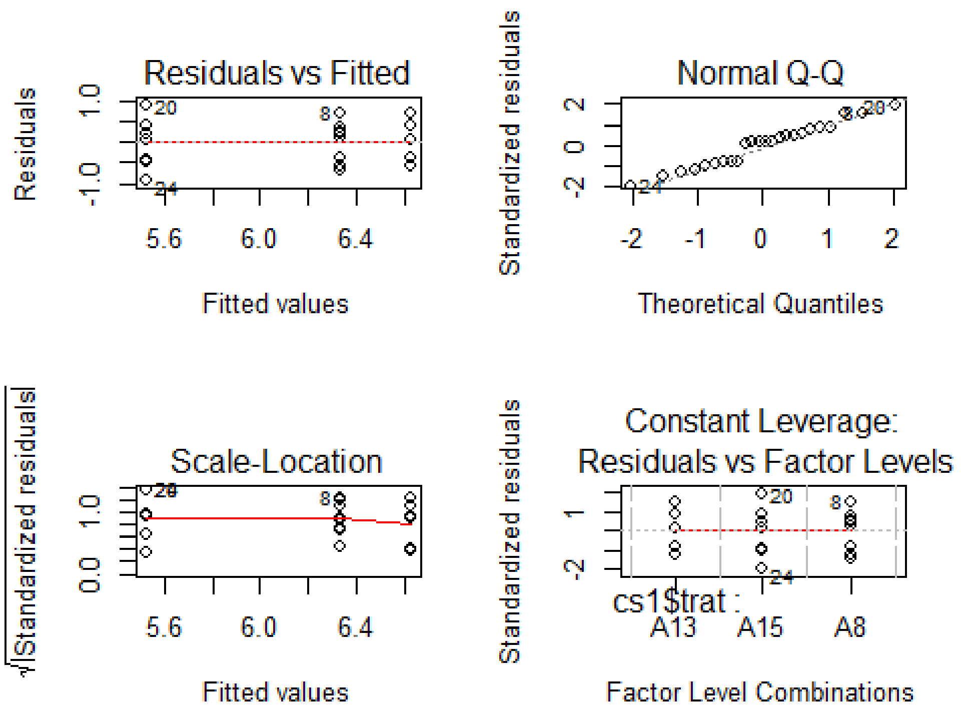

```
shapiro.test(cr1$res)

##
##  Shapiro-Wilk normality test
##
## data:  cr1$res
## W = 0.95865, p-value = 0.4119

require(agricolae)
glr <- df.residual(cr1)
glr

## [1] 21

sqr <- deviance(cr1)
sqr

## [1] 5.286113

qmr <- sqr/glr
qmr

## [1] 0.2517197

lsdr <- LSD.test(rl,cs1$trat, glr, qmr, alpha=0.05, p.adj="none")
lsdr

## $statistics
##     MSerror Df     Mean       CV  t.value       LSD
##   0.2517197 21 6.159571 8.145319 2.079614 0.5216885
##
## $parameters
##         test p.ajusted   name.t ntr alpha
##   Fisher-LSD      none cs1$trat   3  0.05
##
## $means
##           rl       std r      LCL      UCL      Min      Max      Q25
## A13 6.621695 0.4387730 8 6.252805 6.990584 6.040279 7.333030 6.232462
## A15 5.528629 0.5649527 8 5.159739 5.897518 4.625071 6.416749 5.111827
## A8  6.328389 0.4934225 8 5.959500 6.697278 5.634825 7.042295 5.897236
##          Q50      Q75
## A13 6.694525 6.789766
## A15 5.653576 5.785266
## A8  6.490028 6.604979
##
## $comparison
## NULL
##
## $groups
##           rl groups
## A13 6.621695      a
## A8  6.328389      a
## A15 5.528629      b
```

```
##
## attr(,"class")
## [1] "group"

#nematodes on soil

cs<-aov(cs1$solo~cs1$trat)
cs

## Call:
##    aov(formula = cs1$solo ~ cs1$trat)
##
## Terms:
##                 cs1$trat Residuals
## Sum of Squares  18952887  28465785
## Deg. of Freedom        2        21
##
## Residual standard error: 1164.265
## Estimated effects may be unbalanced

summary(cs)

##             Df   Sum Sq Mean Sq F value  Pr(>F)
## cs1$trat     2 18952887 9476443   6.991 0.00471 **
## Residuals   21 28465785 1355514
## ---
## Signif. codes:  0 '***' 0.001 '**' 0.01 '*' 0.05 '.' 0.1 ' ' 1

par(mfrow=c(2,2)); plot(cs); layout(1)
```

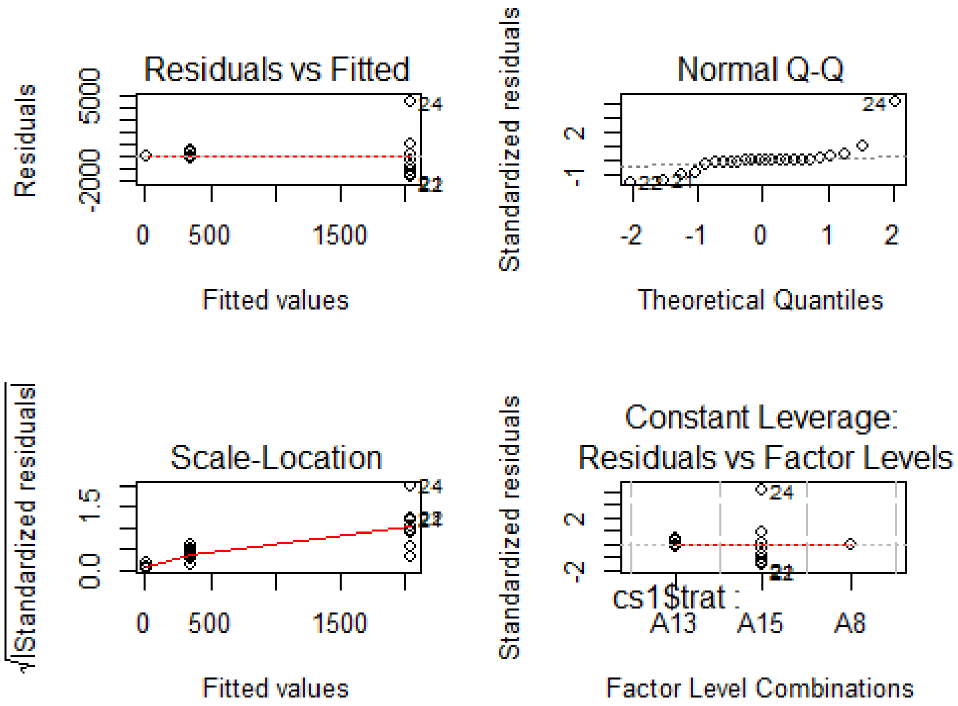


```
shapiro.test(cs$res)

##
##  Shapiro-Wilk normality test
##
## data:  cs$res
## W = 0.66455, p-value = 3.544e-06

# Transforma??o Box-Cox
boxcox(solo ~ trat, data=cs1, plotit=T)
```

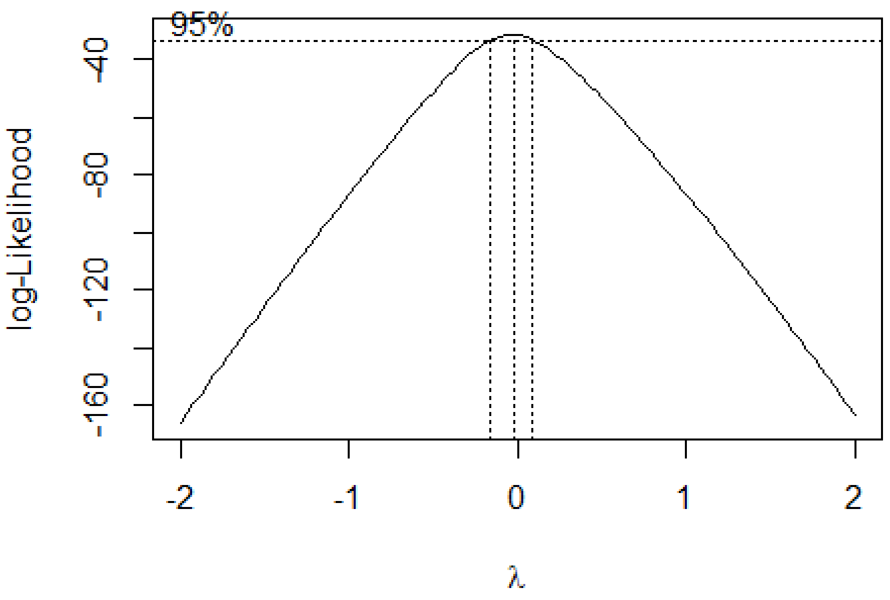


```
bc <- boxcox(solo ~ trat, data=cs1, lam=seq(-.5, .5, 1/10))
```

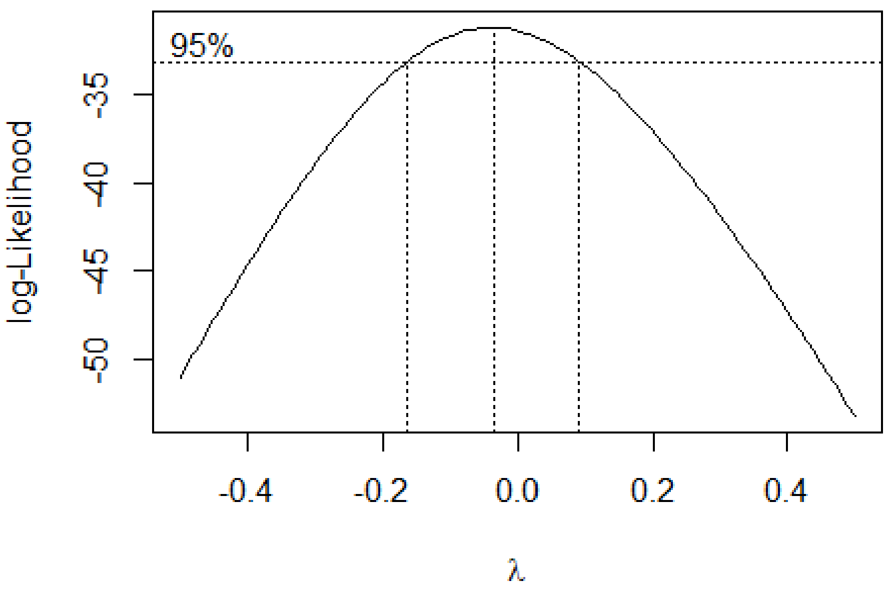


```
(lambda = bc$x[which.max(bc$y)])
```

```
## [1] -0.03535354

sl<-log(cs1$solo+0.01)

cr11<-aov(sl~cs1$trat)
cr11

## Call:
##    aov(formula = sl ~ cs1$trat)
##
## Terms:
##                  cs1$trat Residuals
## Sum of Squares  114.19887  13.66398
## Deg. of Freedom         2        21
##
## Residual standard error: 0.8066384
## Estimated effects may be unbalanced

summary(cr11)

##             Df Sum Sq Mean Sq F value   Pr(>F)
## cs1$trat     2 114.20   57.10   87.75 6.35e-11 ***
## Residuals   21  13.66    0.65
## ---
## Signif. codes:  0 '***' 0.001 '**' 0.01 '*' 0.05 '.' 0.1 ' ' 1

par(mfrow=c(2,2)); plot(cr11); layout(1)
```

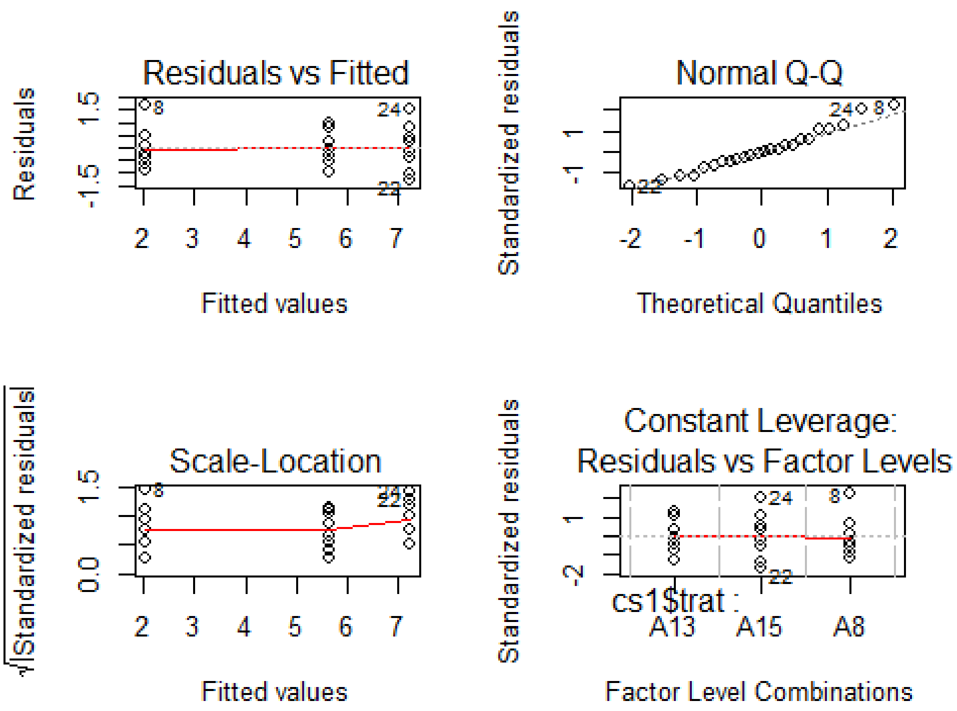

```r
shapiro.test(cr11$res)

##
##  Shapiro-Wilk normality test
##
## data:  cr11$res
## W = 0.96893, p-value = 0.6406

require(agricolae)
glr <- df.residual(cr11)
glr

## [1] 21

sqr <- deviance(cr11)
sqr

## [1] 13.66398

qmr <- sqr/glr
qmr

## [1] 0.6506656

lsdr <- LSD.test(sl,cs1$trat, glr, qmr, alpha=0.05, p.adj="none")
lsdr

## $statistics
##     MSerror Df     Mean       CV  t.value       LSD
##   0.6506656 21 4.974942 16.21403 2.079614 0.8387482
##
## $parameters
##         test p.ajusted   name.t ntr alpha
##   Fisher-LSD      none cs1$trat   3  0.05
##
## $means
##           sl       std r      LCL      UCL      Min      Max      Q25
## A13 5.655199 0.6573244 8 5.062114 6.248284 4.709620 6.637271 5.269942
## A15 7.240647 0.9388672 8 6.647562 7.833731 5.973835 8.776477 6.648582
## A8  2.028980 0.7990304 8 1.435896 2.622065 1.101940 3.689129 1.555775
##          Q50      Q75
## A13 5.565944 6.026027
## A15 7.247051 7.763962
## A8  1.937058 2.181953
##
## $comparison
## NULL
##
## $groups
##           sl groups
## A15 7.240647      a
## A13 5.655199      b
## A8  2.028980      c
```

```
##
## attr(,"class")
## [1] "group"
```

```r
#total nematodes (on soil + in roots)

ct<-aov(cs1$tot~cs1$trat)
ct
```

```
## Call:
##    aov(formula = cs1$tot ~ cs1$trat)
##
## Terms:
##                 cs1$trat Residuals
## Sum of Squares  12068356  29088945
## Deg. of Freedom        2        21
##
## Residual standard error: 1176.94
## Estimated effects may be unbalanced
```

```r
summary(ct)
```

```
##             Df   Sum Sq Mean Sq F value Pr(>F)
## cs1$trat     2 12068356 6034178   4.356 0.0261 *
## Residuals   21 29088945 1385188
## ---
## Signif. codes:  0 '***' 0.001 '**' 0.01 '*' 0.05 '.' 0.1 ' ' 1
```

```r
par(mfrow=c(2,2)); plot(ct); layout(1)
```

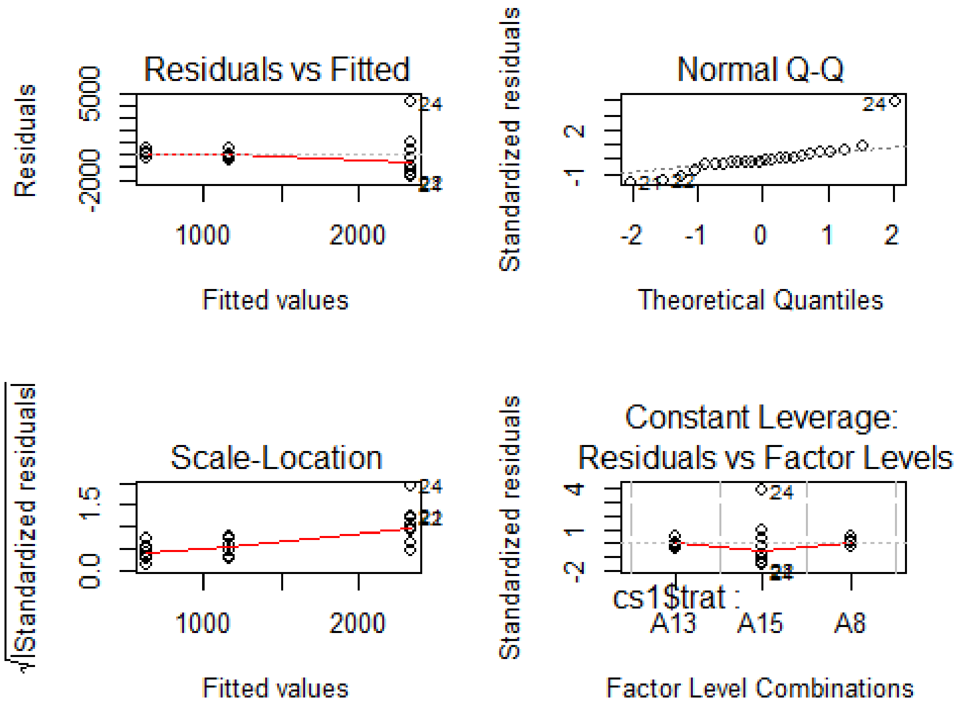


```
shapiro.test(ct$res)

##
##  Shapiro-Wilk normality test
##
## data:  ct$res
## W = 0.77411, p-value = 0.0001144

# Transforma??o Box-Cox
boxcox(tot ~ trat, data=cs1, plotit=T)
```

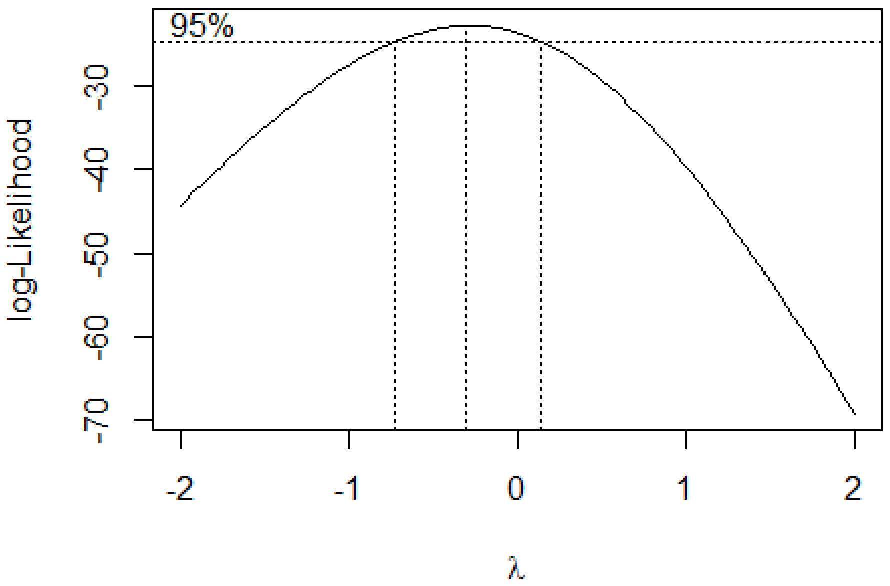


```
bc <- boxcox(tot ~ trat, data=cs1, lam=seq(-.5, .5, 1/10))
```

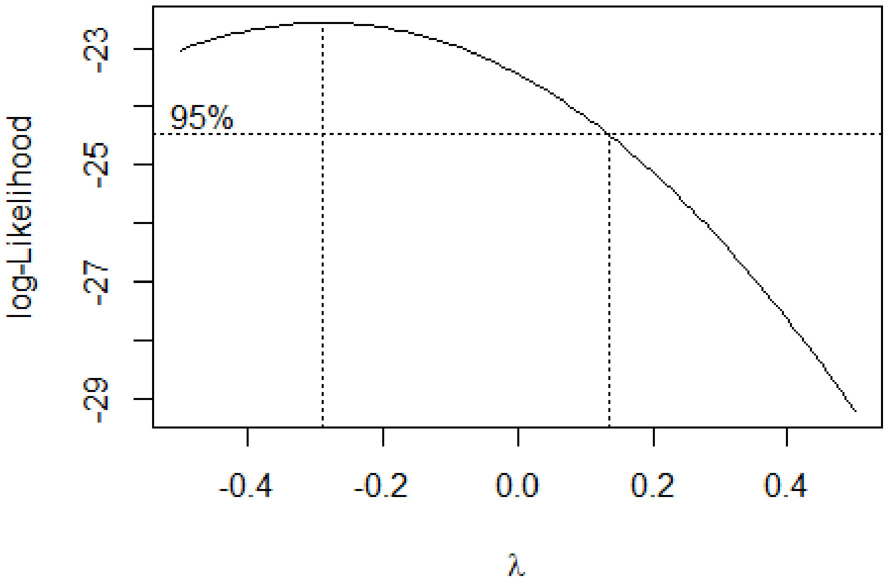


```
(lambda = bc$x[which.max(bc$y)])
```

```
## [1] -0.2878788

tl<-log(cs1$tot+0.01)

cr12<-aov(tl~cs1$trat)
cr12

## Call:
##    aov(formula = tl ~ cs1$trat)
##
## Terms:
##                 cs1$trat Residuals
## Sum of Squares  5.097063  7.052647
## Deg. of Freedom        2        21
##
## Residual standard error: 0.5795173
## Estimated effects may be unbalanced

summary(cr12)

##             Df Sum Sq Mean Sq F value  Pr(>F)
## cs1$trat     2  5.097  2.5485   7.589 0.00331 **
## Residuals   21  7.053  0.3358
## ---
## Signif. codes:  0 '***' 0.001 '**' 0.01 '*' 0.05 '.' 0.1 ' ' 1

par(mfrow=c(2,2)); plot(cr12); layout(1)
```

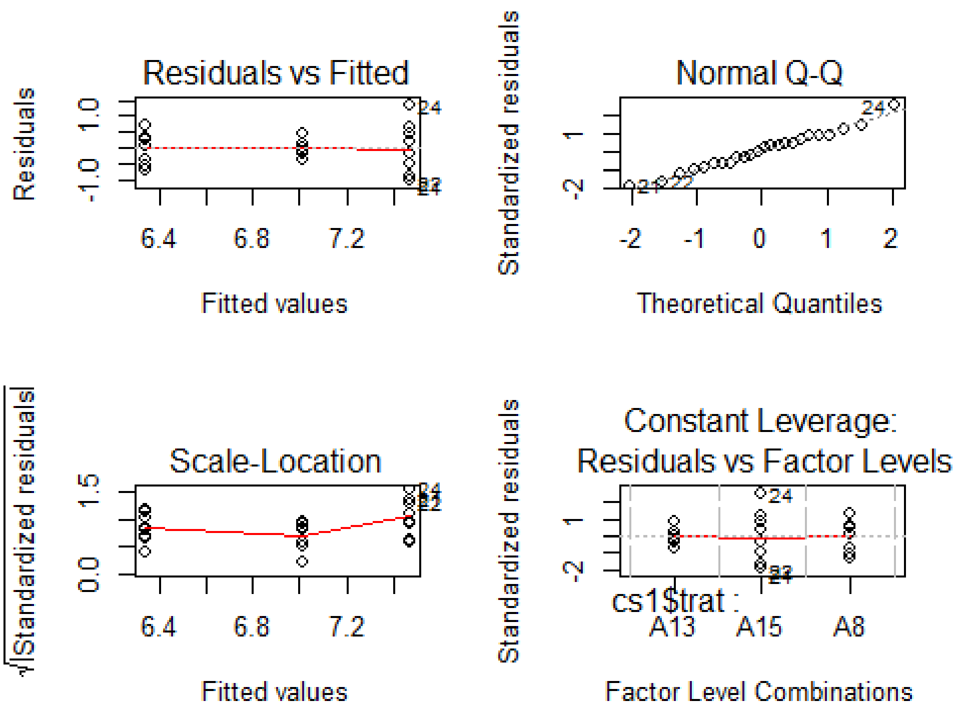

```
shapiro.test(cr12$res)

##
##  Shapiro-Wilk normality test
##
## data:  cr12$res
## W = 0.98524, p-value = 0.9698

require(agricolae)
glr <- df.residual(cr12)
glr

## [1] 21

sqr <- deviance(cr12)
sqr

## [1] 7.052647

qmr <- sqr/glr
qmr

## [1] 0.3358403

lsdr <- LSD.test(tl,cs1$trat, glr, qmr, alpha=0.05, p.adj="none")
lsdr

## $statistics
##     MSerror Df     Mean       CV  t.value       LSD
##   0.3358403 21 6.940165 8.350194 2.079614 0.6025861
##
## $parameters
##         test p.ajusted   name.t ntr alpha
##   Fisher-LSD      none cs1$trat   3  0.05
##
## $means
##            tl       std r      LCL      UCL      Min      Max      Q25
## A13 7.010133 0.3317340 8 6.584040 7.436225 6.637271 7.492209 6.787837
## A15 7.466337 0.8072200 8 7.040244 7.892430 6.461484 8.792095 6.870384
## A8  6.344027 0.4958521 8 5.917934 6.770120 5.645482 7.076662 5.917505
##          Q50      Q75
## A13 6.929125 7.224474
## A15 7.452162 7.975123
## A8  6.501715 6.614828
##
## $comparison
## NULL
##
## $groups
##            tl groups
## A15 7.466337      a
## A13 7.010133      a
## A8  6.344027      b
```

```
##
## attr(,"class")
## [1] "group"
```

```r
#reproduction factor

cs1fr<-aov(cs1$fr~cs1$trat)
cs1fr
```

```
## Call:
##    aov(formula = cs1$fr ~ cs1$trat)
##
## Terms:
##                 cs1$trat Residuals
## Sum of Squares  12.06836  29.08894
## Deg. of Freedom        2        21
##
## Residual standard error: 1.17694
## Estimated effects may be unbalanced
```

```r
summary(cs1fr)
```

```
##             Df Sum Sq Mean Sq F value Pr(>F)
## cs1$trat     2  12.07   6.034   4.356 0.0261 *
## Residuals   21  29.09   1.385
## ---
## Signif. codes:  0 '***' 0.001 '**' 0.01 '*' 0.05 '.' 0.1 ' ' 1
```

```r
par(mfrow=c(2,2)); plot(cs1fr); layout(1)
```

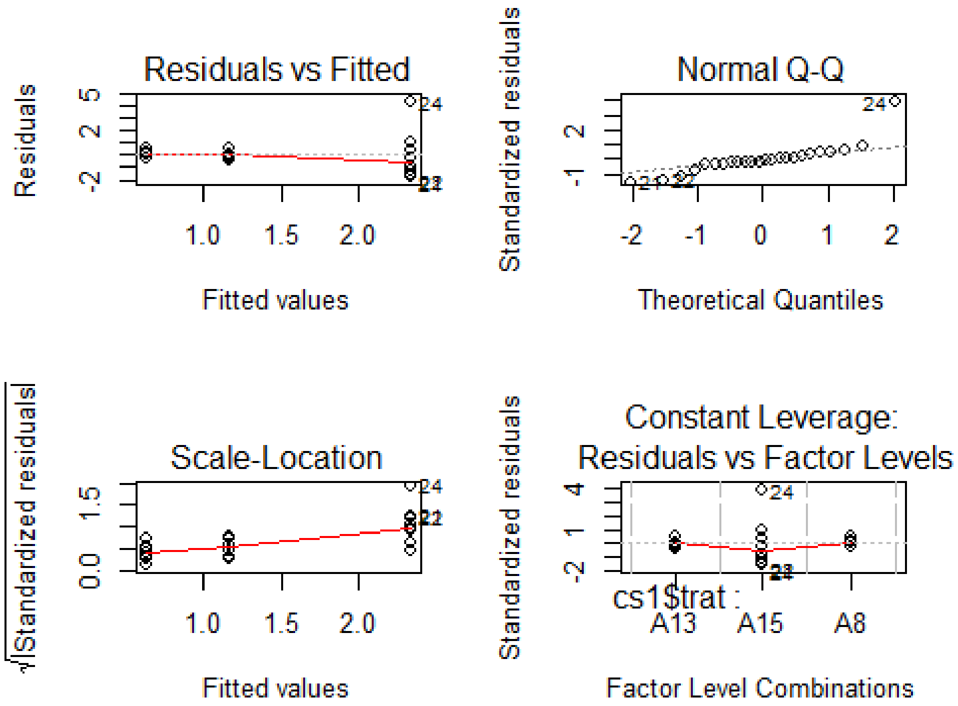


```
shapiro.test(cs1fr$res)

##
##  Shapiro-Wilk normality test
##
## data:  cs1fr$res
## W = 0.77411, p-value = 0.0001144

plot(fr ~ trat, data = cs1)
```

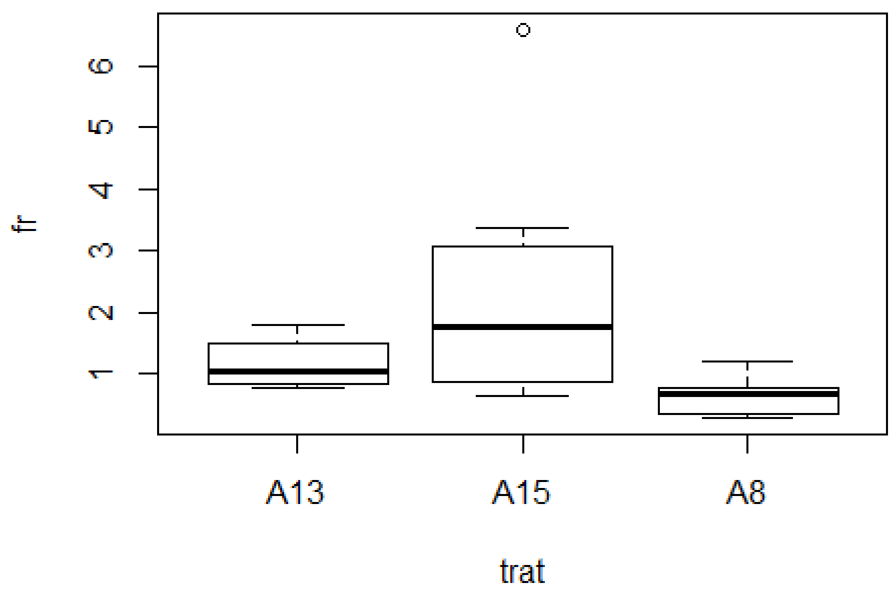


```
bartlett.test(cs1$fr, cs1$trat)
```

```
##
##  Bartlett test of homogeneity of variances
##
## data:  cs1$fr and cs1$trat
## Bartlett's K-squared = 25.258, df = 2, p-value = 3.276e-06
```

```
# Transforma Box-Cox
boxcox(fr+0.01 ~ trat, data=cs1, plotit=T)
```

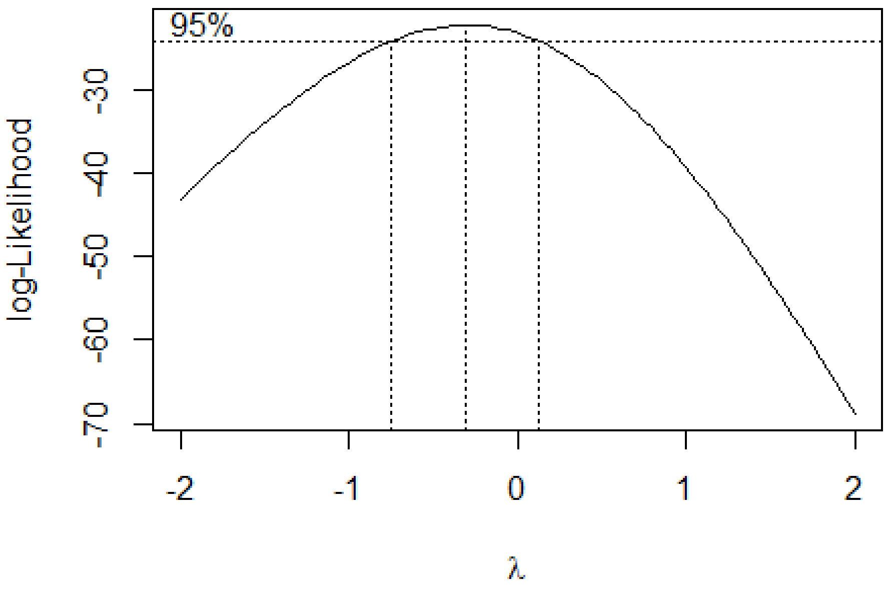


```
bc <- boxcox(fr+0.01 ~ trat, data=cs1, lam=seq(-.5, .5, 1/10))
```

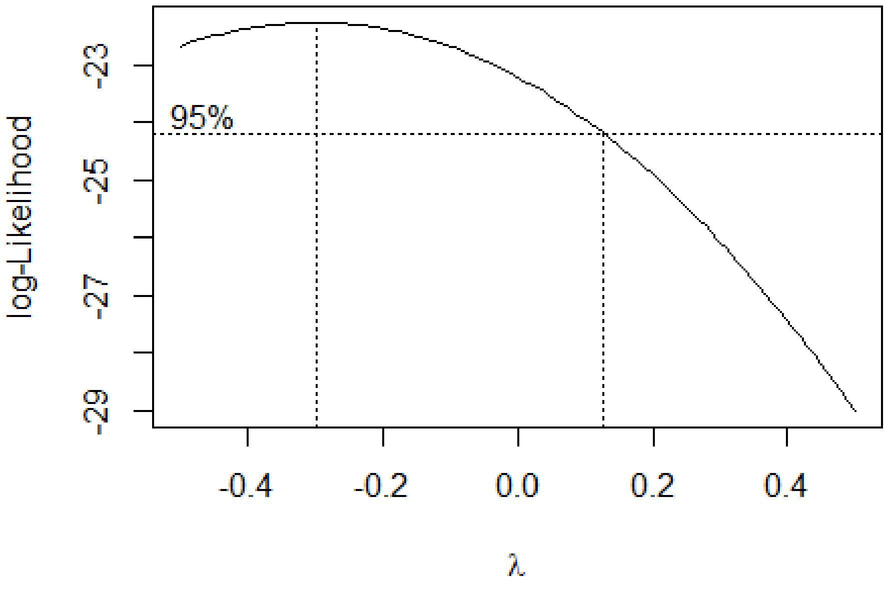


```
(lambda = bc$x[which.max(bc$y)])
```

```
## [1] -0.2979798

fr1<-log(cs1$fr+0.01)

cs1fr1<-aov(fr1~cs1$trat)
cs1fr1

## Call:
##    aov(formula = fr1 ~ cs1$trat)
##
## Terms:
##                 cs1$trat Residuals
## Sum of Squares  4.985887  6.919143
## Deg. of Freedom        2        21
##
## Residual standard error: 0.5740061
## Estimated effects may be unbalanced

summary(cs1fr1)

##             Df Sum Sq Mean Sq F value  Pr(>F)
## cs1$trat     2  4.986  2.4929   7.566 0.00335 **
## Residuals   21  6.919  0.3295
## ---
## Signif. codes:  0 '***' 0.001 '**' 0.01 '*' 0.05 '.' 0.1 ' ' 1

par(mfrow=c(2,2)); plot(cs1fr1); layout(1)
```

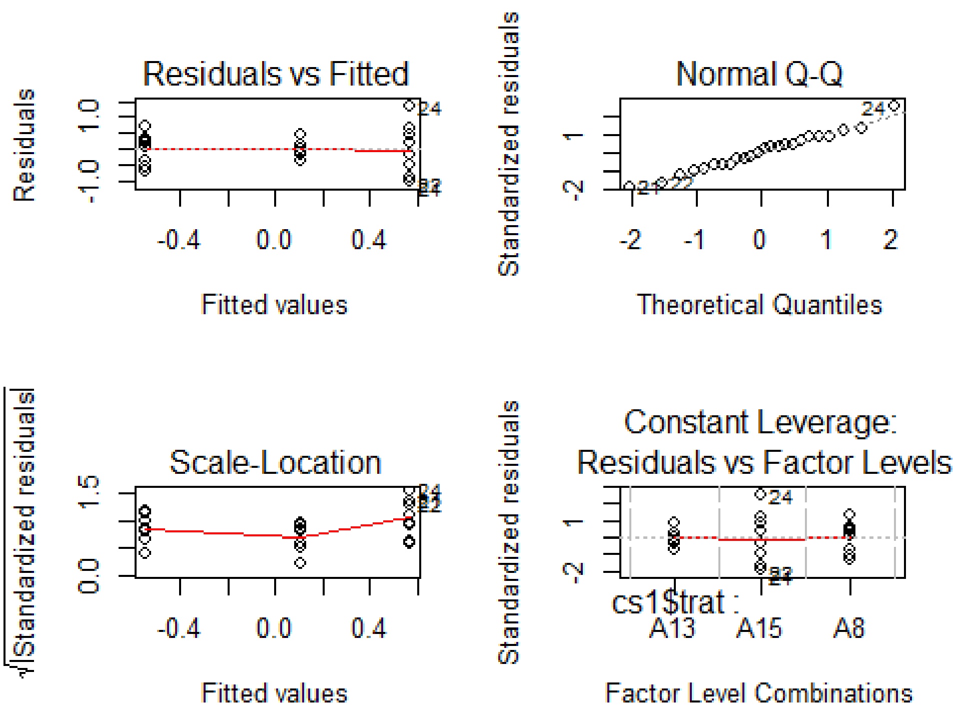


```
shapiro.test(cs1fr1$res)
```

```
##
##  Shapiro-Wilk normality test
##
## data:  cs1fr1$res
## W = 0.98496, p-value = 0.9671
```

```
plot(fr1 ~ trat, data = cs1)
```

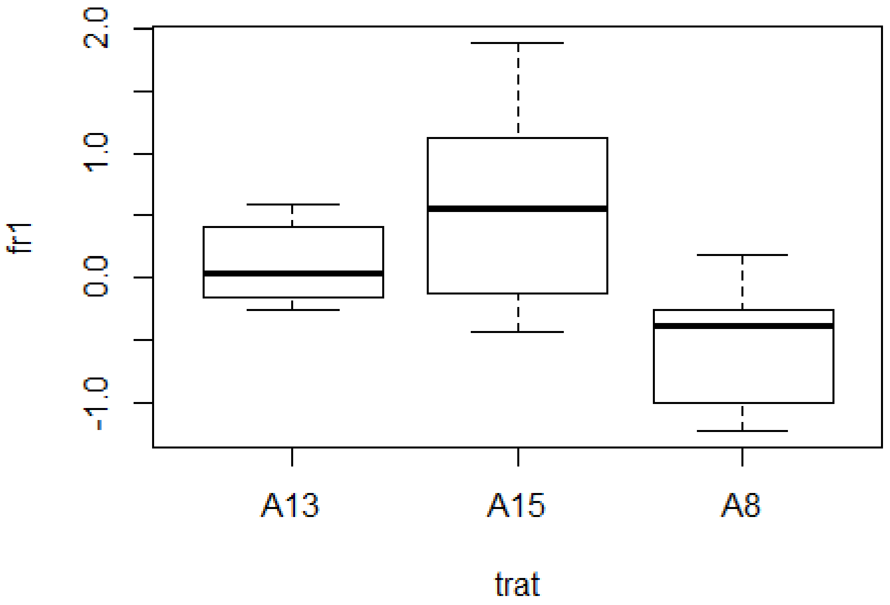


```
bartlett.test(fr1, cs1$trat)
```

```
##
##  Bartlett test of homogeneity of variances
##
## data:  fr1 and cs1$trat
## Bartlett's K-squared = 5.1019, df = 2, p-value = 0.07801
```

```
require(agricolae)
glr <- df.residual(cs1fr1)
glr
```

```
## [1] 21
```

```
sqr <- deviance(cs1fr1)
sqr
```

```
## [1] 6.919143
```

```
qmr <- sqr/glr
qmr
```

```
## [1] 0.329483
```

```
lsd <- LSD.test(fr1,cs1$trat, glr, qmr, alpha=0.05, p.adj="none")
lsd
```

```
## $statistics
##    MSerror Df       Mean       CV  t.value       LSD
##   0.329483 21 0.04443006 1291.932 2.079614 0.5968555
## 
## $parameters
##         test p.ajusted   name.t ntr alpha
##   Fisher-LSD      none cs1$trat   3  0.05
## 
## $means
##              fr1       std r        LCL        UCL        Min       Max
## A13  0.1117685 0.3288829 8 -0.3102721  0.5338091 -0.2574762 0.5900064
## A15  0.5659339 0.8022470 8  0.1438933  0.9879744 -0.4307829 1.8858568
## A8  -0.5444122 0.4865026 8 -0.9664528 -0.1223716 -1.2275827 0.1773090
##              Q25         Q50        Q75
## A13 -0.10868576  0.03111919  0.3240339
## A15 -0.02686655  0.55029517  1.0708088
## A8  -0.96363849 -0.39112036 -0.2796146
## 
## $comparison
## NULL
## 
## $groups
##              fr1 groups
## A15  0.5659339      a
## A13  0.1117685      a
## A8  -0.5444122      b
## 
## attr(,"class")
## [1] "group"
```

```
#nematodes per gram of roots

cs1n<-aov(cs1$nema~cs1$trat)
cs1n
```

```
## Call:
##    aov(formula = cs1$nema ~ cs1$trat)
## 
## Terms:
##                 cs1$trat Residuals
## Sum of Squares  11320.75  24183.88
## Deg. of Freedom        2        21
## 
## Residual standard error: 33.93543
## Estimated effects may be unbalanced
```

```
summary(cs1n)

##              Df Sum Sq Mean Sq F value Pr(>F)
## cs1$trat      2  11321    5660   4.915 0.0177 *
## Residuals    21  24184    1152
## ---
## Signif. codes:  0 '***' 0.001 '**' 0.01 '*' 0.05 '.' 0.1 ' ' 1

par(mfrow=c(2,2)); plot(cs1n); layout(1)
```

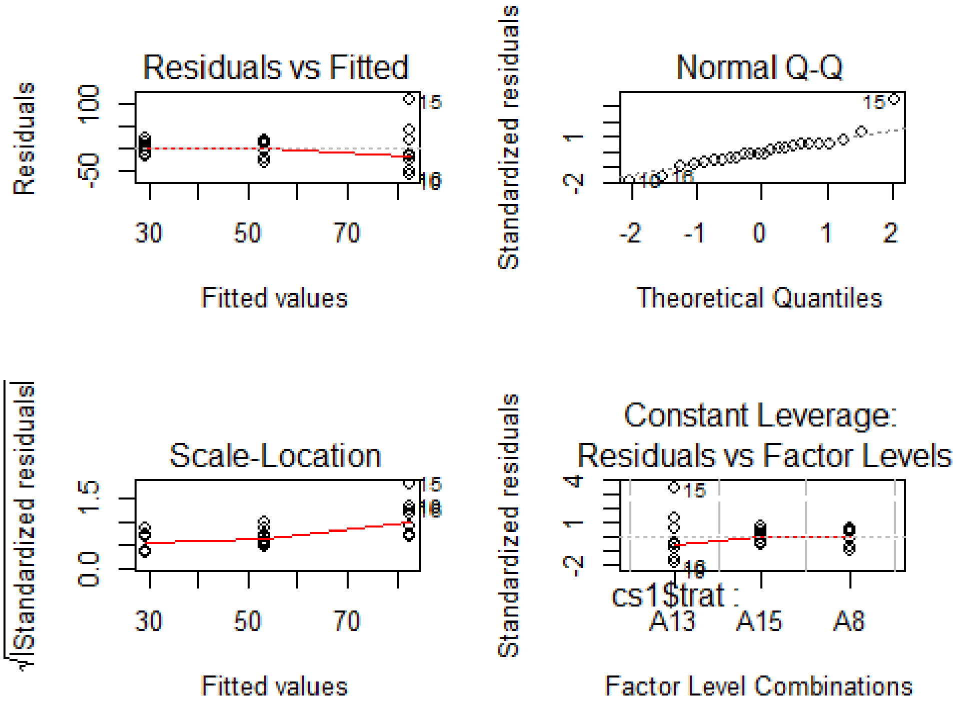


```
shapiro.test(cs1n$res)

##
##  Shapiro-Wilk normality test
##
## data:  cs1n$res
## W = 0.89235, p-value = 0.01487

plot(nema ~ trat, data = cs1)
```

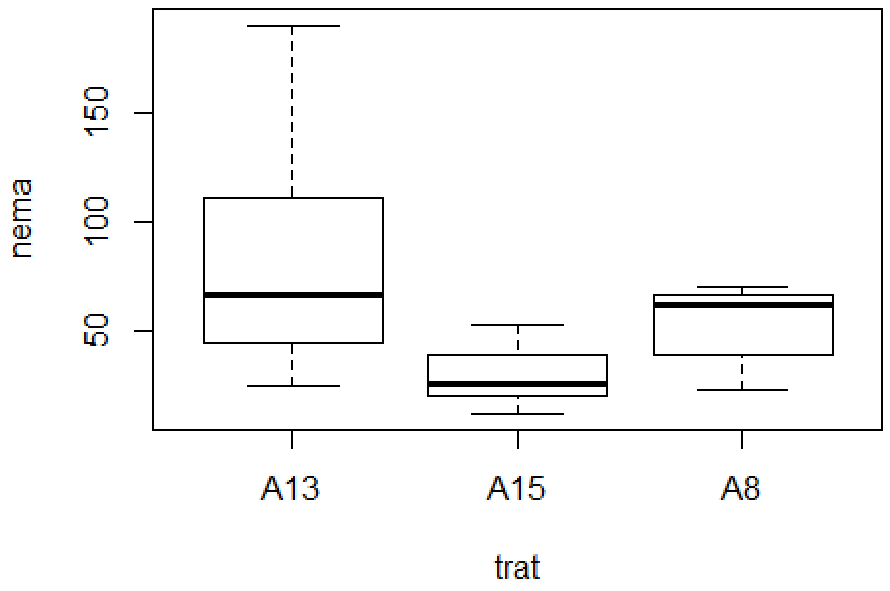


```
bartlett.test(cs1$nema, cs1$trat)

##
##  Bartlett test of homogeneity of variances
##
## data:  cs1$nema and cs1$trat
## Bartlett's K-squared = 14.025, df = 2, p-value = 0.0009007

# Transforma Box-Cox
boxcox(nema+0.01 ~ trat, data=cs1, plotit=T)
```

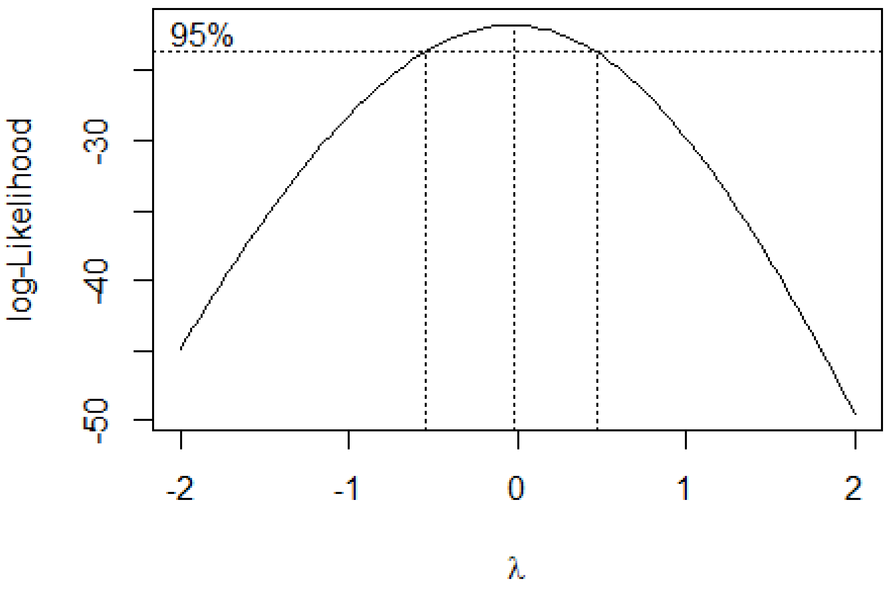


```
bc <- boxcox(nema+0.01 ~ trat, data=cs1, lam=seq(-.5, .5, 1/10))
```

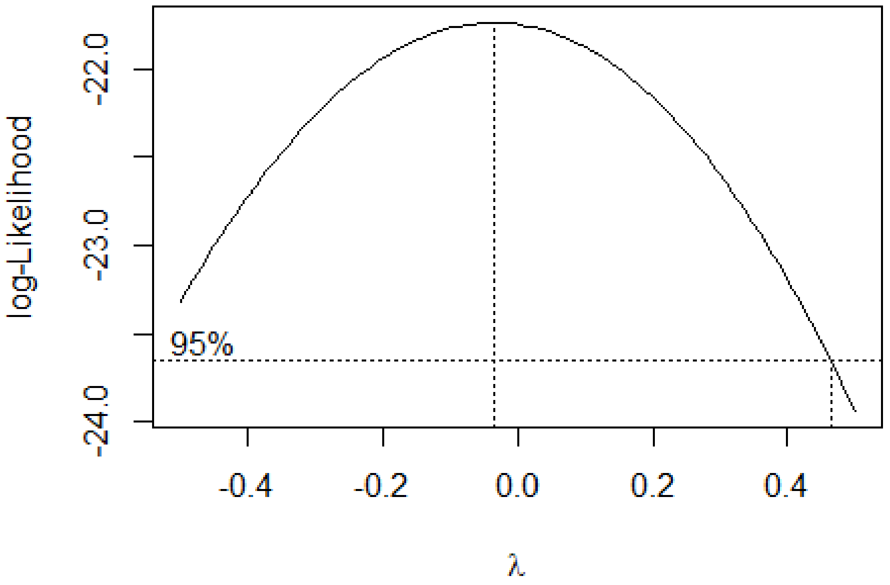


```
(lambda = bc$x[which.max(bc$y)])
```

```
## [1] -0.03535354
```

```
n1<-log(cs1$nema+0.01)
```

```
cs1n1<-aov(n1~cs1$trat)
cs1n1
```

```
## Call:
##    aov(formula = n1 ~ cs1$trat)
##
## Terms:
##                 cs1$trat Residuals
## Sum of Squares  3.727281  6.123970
## Deg. of Freedom        2        21
##
## Residual standard error: 0.5400163
## Estimated effects may be unbalanced
```

```
summary(cs1n1)
```

```
##             Df Sum Sq Mean Sq F value  Pr(>F)
## cs1$trat     2  3.727  1.8636   6.391 0.00679 **
## Residuals   21  6.124  0.2916
## ---
## Signif. codes:  0 '***' 0.001 '**' 0.01 '*' 0.05 '.' 0.1 ' ' 1
```

```
par(mfrow=c(2,2)); plot(cs1n1); layout(1)
```

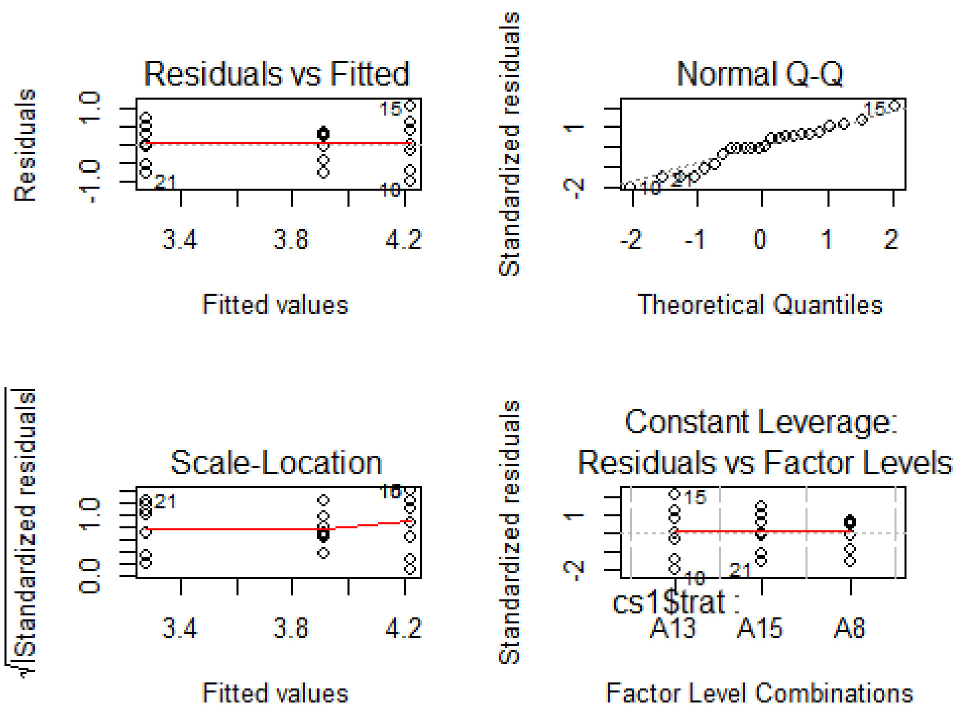


```
shapiro.test(cs1n1$res)
```

```
##
##  Shapiro-Wilk normality test
##
## data:  cs1n1$res
## W = 0.96184, p-value = 0.4766
```

```r
plot(n1 ~ trat, data = cs1)
```

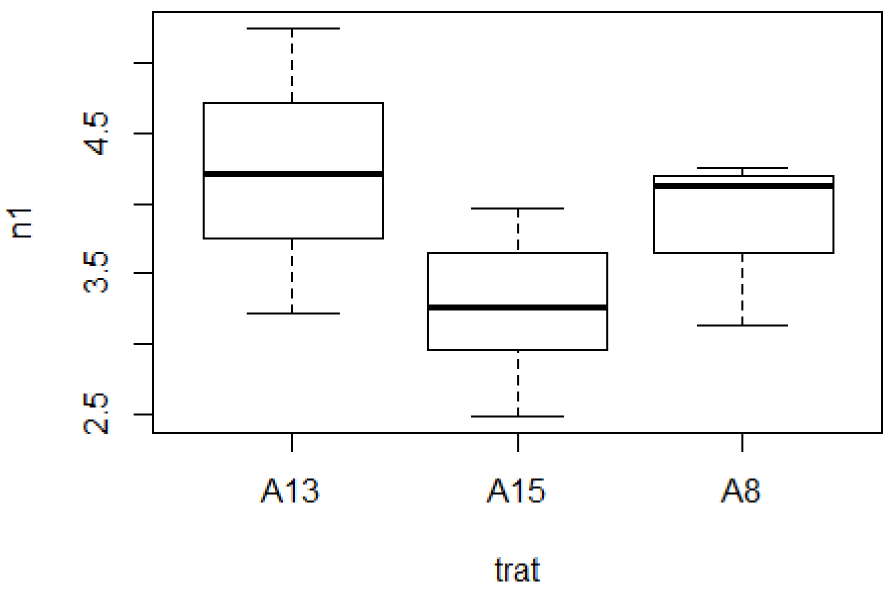


```r
bartlett.test(n1, cs1$trat)
```

```
##
##  Bartlett test of homogeneity of variances
##
## data:  n1 and cs1$trat
## Bartlett's K-squared = 1.5801, df = 2, p-value = 0.4538
```

```r
require(agricolae)
glr <- df.residual(cs1n1)
glr
```

```
## [1] 21
```

```r
sqr <- deviance(cs1n1)
sqr
```

```
## [1] 6.12397
```

```r
qmr <- sqr/glr
qmr
```

```
## [1] 0.2916176
```

```
lsdn <- LSD.test(n1,cs1$trat, glr, qmr, alpha=0.05, p.adj="none")
lsdn
```

```
## $statistics
##     MSerror Df    Mean       CV  t.value       LSD
##   0.2916176 21 3.80467 14.19351 2.079614 0.5615127
##
## $parameters
##         test p.ajusted   name.t ntr alpha
##   Fisher-LSD      none cs1$trat   3  0.05
##
## $means
##           n1       std r      LCL      UCL      Min      Max      Q25
## A13 4.222872 0.6723805 8 3.825822 4.619921 3.219276 5.247077 3.885660
## A15 3.276531 0.5005383 8 2.879481 3.673580 2.485740 3.970481 3.091636
## A8  3.914607 0.4149925 8 3.517558 4.311657 3.135929 4.248638 3.746348
##          Q50      Q75
## A13 4.204730 4.651506
## A15 3.258481 3.591095
## A8  4.127166 4.177842
##
## $comparison
## NULL
##
## $groups
##           n1 groups
## A13 4.222872      a
## A8  3.914607      a
## A15 3.276531      b
##
## attr(,"class")
## [1] "group"
```
