# Supplementary material for: Two novel potential pathogens for soybean
Source: PLoS One. 2019 Aug 22;14(8):e0221416. doi: 10.1371/journal.pone.0221416 (PMC6705753; doi:10.1371/journal.pone.0221416)
Supplement: S8 File — (PDF) [file pone.0221416.s008.pdf]

70_dai_experiment_2_nematode.R

Santino

Tue Jul 23 19:11:01 2019

```r
rm(list = ls())
cs1<-read.table("C:\\analises nemato\\soja comparativo analises\\Soja
comparativo esq n.txt",h=T,dec=",")
cs1
```

```
##    trat raiz solo  tot    fr nema
## 1    A8 2040   20 2060 2.060   52
## 2    A8 1134   11 1145 1.145   50
## 3    A8  924    5  929 0.929   46
## 4    A8 1320    7 1327 1.327   54
## 5    A8 4620   31 4651 4.651  178
## 6    A8 1218   16 1234 1.234   36
## 7    A8  946   68 1014 1.014   27
## 8   A13  450  328  778 0.778   29
## 9   A13  506  483  989 0.989   65
## 10  A13 1120  740 1860 1.860   56
## 11  A13  270  299  569 0.569   13
## 12  A13   96  302  398 0.398   10
## 13  A13  450  467  917 0.917   28
## 14  A13  352 1416 1768 1.768   41
## 15  A15  232 4752 4984 4.984  144
## 16  A15   92 3316 3408 3.408   82
## 17  A15  336 2544 2880 2.880   73
## 18  A15   44 2088 2132 2.132   81
## 19  A15  120 1992 2112 2.112   56
## 20  A15  184 2184 2368 2.368   72
## 21  A15  168 3072 3240 3.240   98
```

```r
data.frame(table(cs1$trat))
```

```
##   Var1 Freq
## 1  A13    7
## 2  A15    7
## 3   A8    7
```

```r
require(graphics)
require(ExpDes)

require(MASS)

require(agricolae)

attach(cs1)
```

```
# mean and median

(Medias = with(cs1 [, 2:6], aggregate(. ~trat, data=cs1[ ,2:6], mean)))

##   trat      raiz       solo      tot       fr     nema
## 1  A13  463.4286  576.42857 1039.857 1.039857 34.57143
## 2  A15  168.0000 2849.71429 3017.714 3.017714 86.57143
## 3   A8 1743.1429   22.57143 1765.714 1.765714 63.28571

(Medianas = with(cs1 [, 2:6], aggregate(. ~trat, data=cs1[ ,2:6],
median)))

##   trat raiz solo  tot    fr nema
## 1  A13  450  467  917 0.917   29
## 2  A15  168 2544 2880 2.880   81
## 3   A8 1218   16 1234 1.234   50

#standard deviation
sd(cs1$raiz)

## [1] 1024.327

sd(cs1$solo)

## [1] 1380.779

sd(cs1$tot)

## [1] 1276.489

sd(cs1$fr)

## [1] 1.276489

sd(cs1$nema)

## [1] 40.49027

#variation coef
require(raster)

cv(cs1$raiz, na.rm=TRUE)

## [1] 129.412

cv(cs1$solo, na.rm=TRUE)

## [1] 120.1125

cv(cs1$tot, na.rm=TRUE)

## [1] 65.76127

cv(cs1$fr, na.rm=TRUE)

## [1] 65.76127
```

```r
cv(cs1$nema, na.rm=TRUE)

## [1] 65.86333

#nematodes in roots
cr<-aov(cs1$raiz~cs1$trat)
cr

## Call:
##    aov(formula = cs1$raiz ~ cs1$trat)
##
## Terms:
##                  cs1$trat Residuals
## Sum of Squares    9814051  11170869
## Deg. of Freedom         2        18
##
## Residual standard error: 787.7841
## Estimated effects may be unbalanced

summary(cr)

##             Df   Sum Sq Mean Sq F value  Pr(>F)
## cs1$trat     2  9814051 4907025   7.907 0.00343 **
## Residuals   18 11170869  620604
## ---
## Signif. codes:  0 '***' 0.001 '**' 0.01 '*' 0.05 '.' 0.1 ' ' 1

par(mfrow=c(2,2)); plot(cr); layout(1)
```

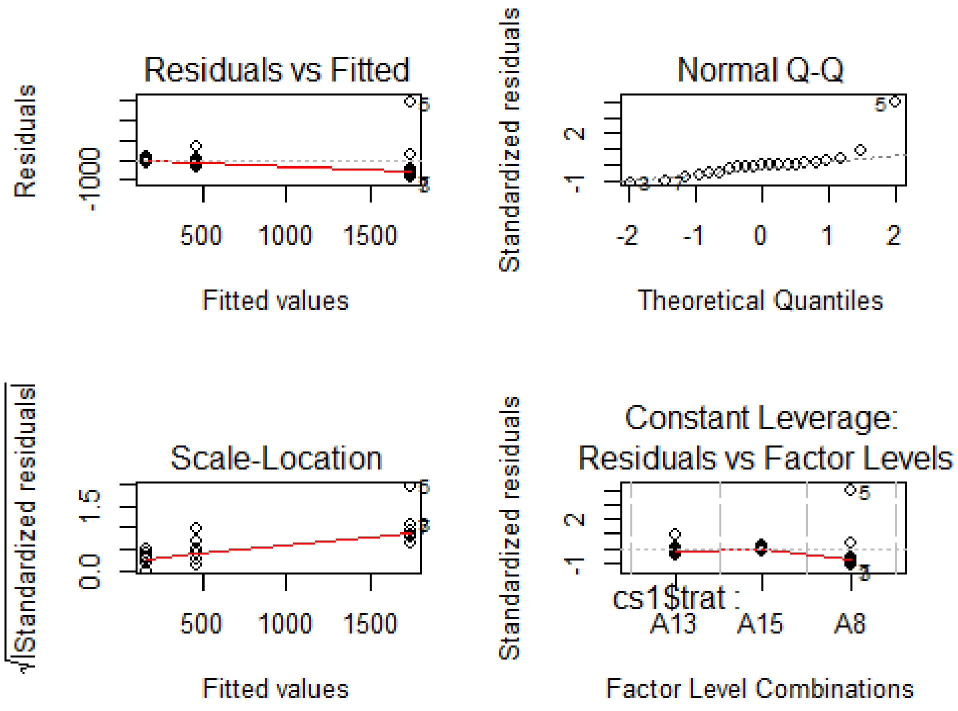

```r
shapiro.test(cr$res)
```

```
##
##  Shapiro-Wilk normality test
##
## data:  cr$res
## W = 0.6752, p-value = 1.373e-05
```

```r
# Transforma??o Box-Cox
boxcox(raiz ~ trat, data=cs1, plotit=T)
```

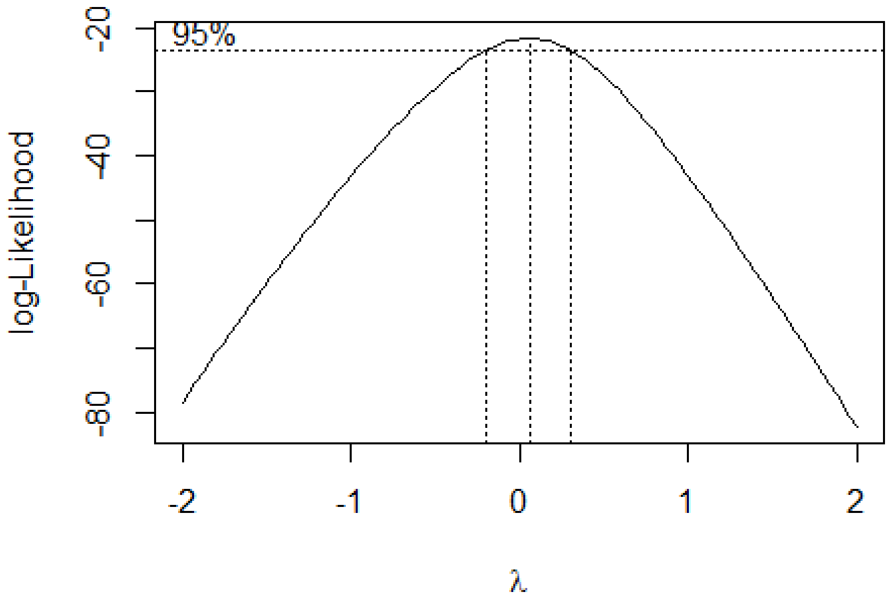


```r
bc <- boxcox(raiz ~ trat, data=cs1, lam=seq(-.5, .5, 1/10))
```

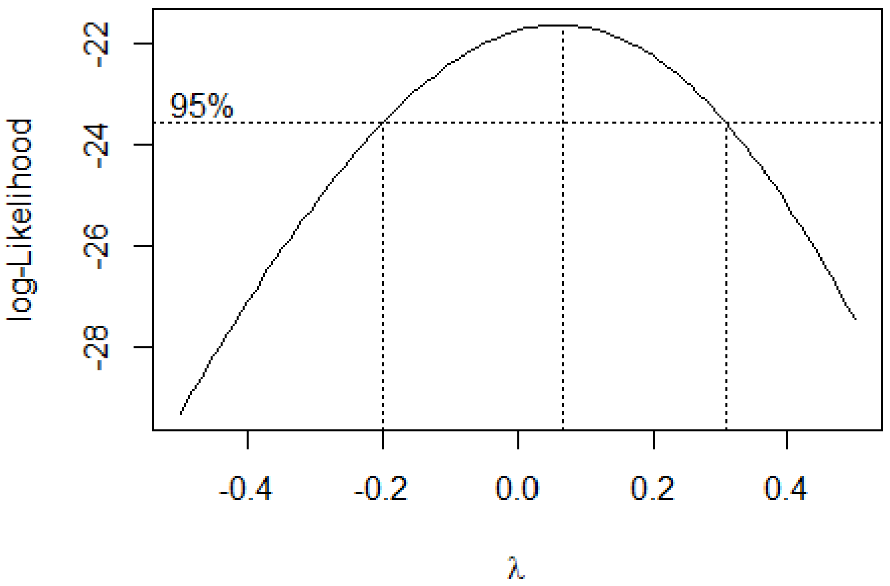


```
(lambda = bc$x[which.max(bc$y)])
```

```
## [1] 0.06565657
```

```
rl<-log(cs1$raiz+0.01)

cr1<-aov(rl~cs1$trat)
cr1
```

```
## Call:
##    aov(formula = rl ~ cs1$trat)
##
## Terms:
##                  cs1$trat Residuals
## Sum of Squares  19.317304  7.931676
## Deg. of Freedom         2        18
##
## Residual standard error: 0.6638137
## Estimated effects may be unbalanced
```

```
summary(cr1)
```

```
##             Df Sum Sq Mean Sq F value  Pr(>F)
## cs1$trat     2 19.317   9.659   21.92 1.5e-05 ***
## Residuals   18  7.932   0.441
## ---
## Signif. codes:  0 '***' 0.001 '**' 0.01 '*' 0.05 '.' 0.1 ' ' 1
```

```r
par(mfrow=c(2,2)); plot(cr1); layout(1)
```

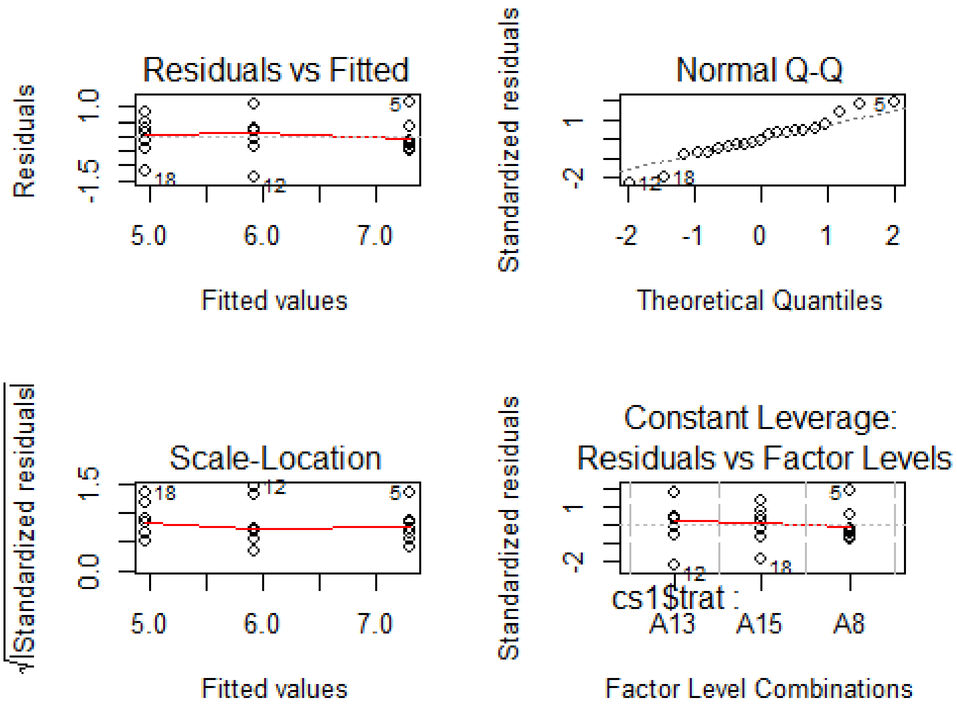


```r
shapiro.test(cr1$res)

##
##  Shapiro-Wilk normality test
##
## data:  cr1$res
## W = 0.95961, p-value = 0.5082

require(agricolae)
glr <- df.residual(cr1)
glr

## [1] 18

sqr <- deviance(cr1)
sqr

## [1] 7.931676

qmr <- sqr/glr
qmr

## [1] 0.4406487

lsdr <- LSD.test(rl,cs1$trat, glr, qmr, alpha=0.05, p.adj="none")
lsdr
```

```
## $statistics
##     MSerror Df     Mean      CV  t.value       LSD
##   0.4406487 18 6.059681 10.9546 2.100922 0.7454562
##
## $parameters
##         test p.ajusted   name.t ntr alpha
##   Fisher-LSD      none cs1$trat   3  0.05
##
## $means
##           rl       std r      LCL      UCL      Min      Max      Q25
## A13 5.927537 0.7440546 7 5.400420 6.454654 4.564452 7.021093 5.731059
## A15 4.956689 0.6667274 7 4.429572 5.483806 3.784417 5.817141 4.654736
## A8  7.294817 0.5690372 7 6.767700 7.821935 6.828723 8.438152 6.942884
##          Q50      Q75
## A13 6.109270 6.167913
## A15 5.124024 5.330885
## A8  7.104974 7.403052
##
## $comparison
## NULL
##
## $groups
##           rl groups
## A8  7.294817      a
## A13 5.927537      b
## A15 4.956689      c
##
## attr(,"class")
## [1] "group"
```

```
#nematodes on soil

cs<-aov(cs1$solo~cs1$trat)
cs
```

```
## Call:
##    aov(formula = cs1$solo ~ cs1$trat)
##
## Terms:
##                 cs1$trat Residuals
## Sum of Squares  31423752   6707247
## Deg. of Freedom        2        18
##
## Residual standard error: 610.43
## Estimated effects may be unbalanced
```

```
summary(cs)
```

```
##             Df   Sum Sq  Mean Sq F value   Pr(>F)
## cs1$trat     2 31423752 15711876   42.16 1.61e-07 ***
## Residuals   18  6707247   372625
```

```
## ---
## Signif. codes:  0 '***' 0.001 '**' 0.01 '*' 0.05 '.' 0.1 ' ' 1
par(mfrow=c(2,2)); plot(cs); layout(1)
```

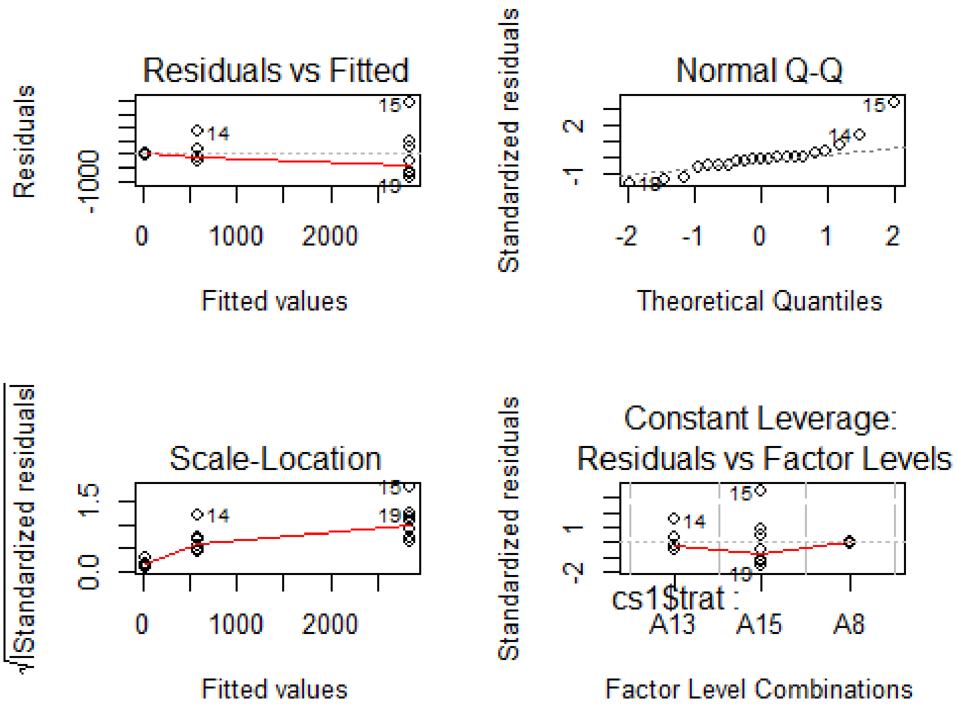


```
shapiro.test(cs$res)

##
##  Shapiro-Wilk normality test
##
## data:  cs$res
## W = 0.83129, p-value = 0.002056

# Transforma??o Box-Cox
boxcox(solo ~ trat, data=cs1, plotit=T)
```

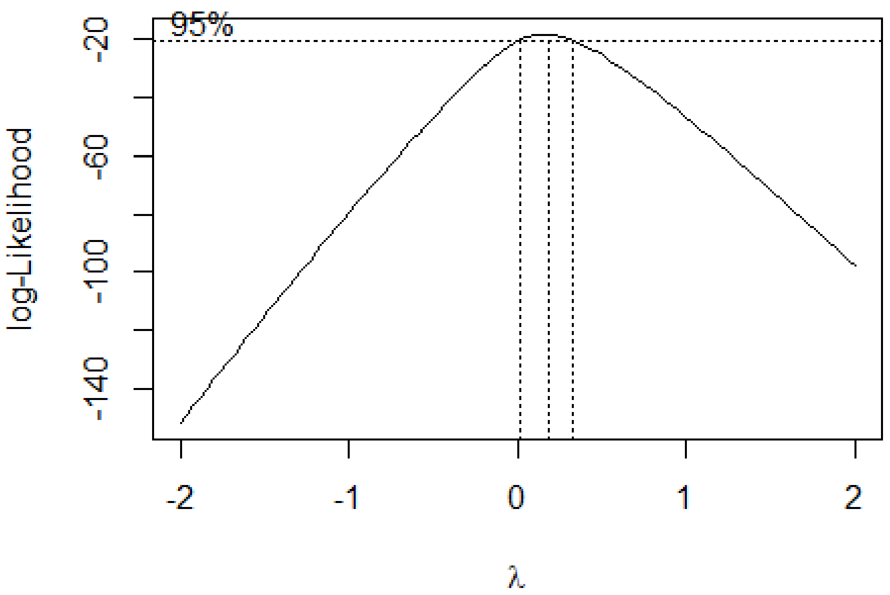


```
bc <- boxcox(solo ~ trat, data=cs1, lam=seq(-.5, .5, 1/10))
```

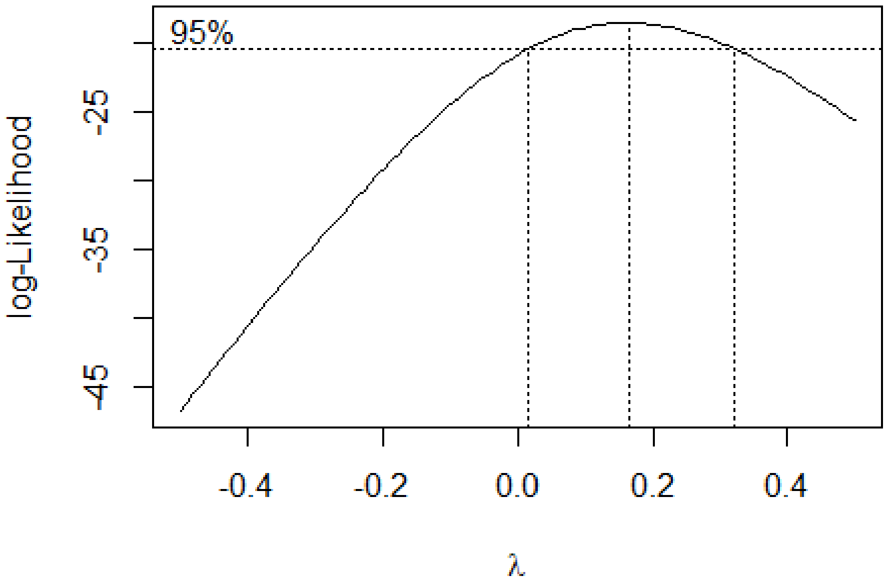


```
(lambda = bc$x[which.max(bc$y)])
```

```
## [1] 0.1666667

sl<-log(cs1$solo+0.01)

cr11<-aov(sl~cs1$trat)
cr11

## Call:
##    aov(formula = sl ~ cs1$trat)
##
## Terms:
##                 cs1$trat Residuals
## Sum of Squares  95.98234   7.27344
## Deg. of Freedom        2        18
##
## Residual standard error: 0.635673
## Estimated effects may be unbalanced

summary(cr11)

##             Df Sum Sq Mean Sq F value   Pr(>F)
## cs1$trat     2  95.98   47.99   118.8 4.27e-11 ***
## Residuals   18   7.27    0.40
## ---
## Signif. codes:  0 '***' 0.001 '**' 0.01 '*' 0.05 '.' 0.1 ' ' 1

par(mfrow=c(2,2)); plot(cr11); layout(1)
```

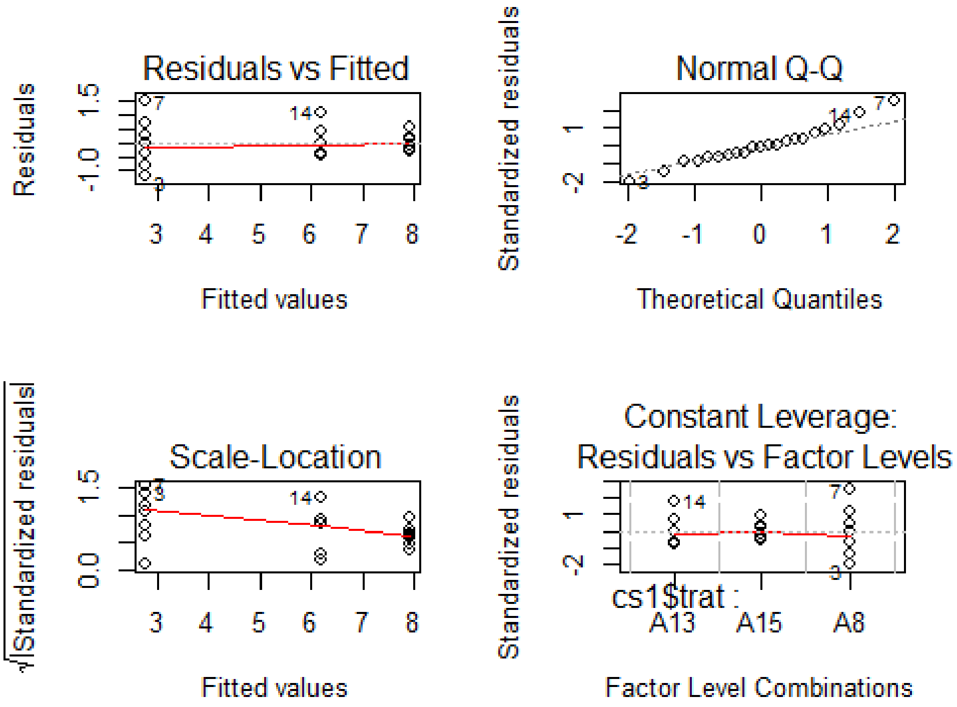

```r
shapiro.test(cr11$res)

##
##  Shapiro-Wilk normality test
##
## data:  cr11$res
## W = 0.97043, p-value = 0.7426

require(agricolae)
glr <- df.residual(cr11)
glr

## [1] 18

sqr <- deviance(cr11)
sqr

## [1] 7.273443

qmr <- sqr/glr
qmr

## [1] 0.4040802

lsdr <- LSD.test(sl,cs1$trat, glr, qmr, alpha=0.05, p.adj="none")
lsdr

## $statistics
##     MSerror Df     Mean       CV  t.value       LSD
##   0.4040802 18 5.626087 11.29867 2.100922 0.7138545
##
## $parameters
##         test p.ajusted   name.t ntr alpha
##   Fisher-LSD      none cs1$trat   3  0.05
##
## $means
##            sl       std r      LCL      UCL      Min      Max      Q25
## A13 6.198947 0.5682895 7 5.694176 6.703719 5.700477 7.255598 5.751752
## A15 7.910601 0.3122255 7 7.405830 8.415373 7.596899 8.466323 7.666442
## A8  2.768713 0.8898330 7 2.263941 3.273484 1.611436 4.219655 2.173071
##          Q50      Q75
## A13 6.146351 6.393351
## A15 7.841497 8.068302
## A8  2.773214 3.215271
##
## $comparison
## NULL
##
## $groups
##            sl groups
## A15 7.910601      a
## A13 6.198947      b
## A8  2.768713      c
```

```
##
## attr(,"class")
## [1] "group"

#total nematodes (on soil + in roots)

ct<-aov(cs1$tot~cs1$trat)
ct

## Call:
##    aov(formula = cs1$tot ~ cs1$trat)
##
## Terms:
##                 cs1$trat Residuals
## Sum of Squares  14014680  18573798
## Deg. of Freedom        2        18
##
## Residual standard error: 1015.814
## Estimated effects may be unbalanced

summary(ct)

##             Df   Sum Sq Mean Sq F value  Pr(>F)
## cs1$trat     2 14014680 7007340   6.791 0.00635 **
## Residuals   18 18573798 1031878
## ---
## Signif. codes:  0 '***' 0.001 '**' 0.01 '*' 0.05 '.' 0.1 ' ' 1

par(mfrow=c(2,2)); plot(ct); layout(1)
```

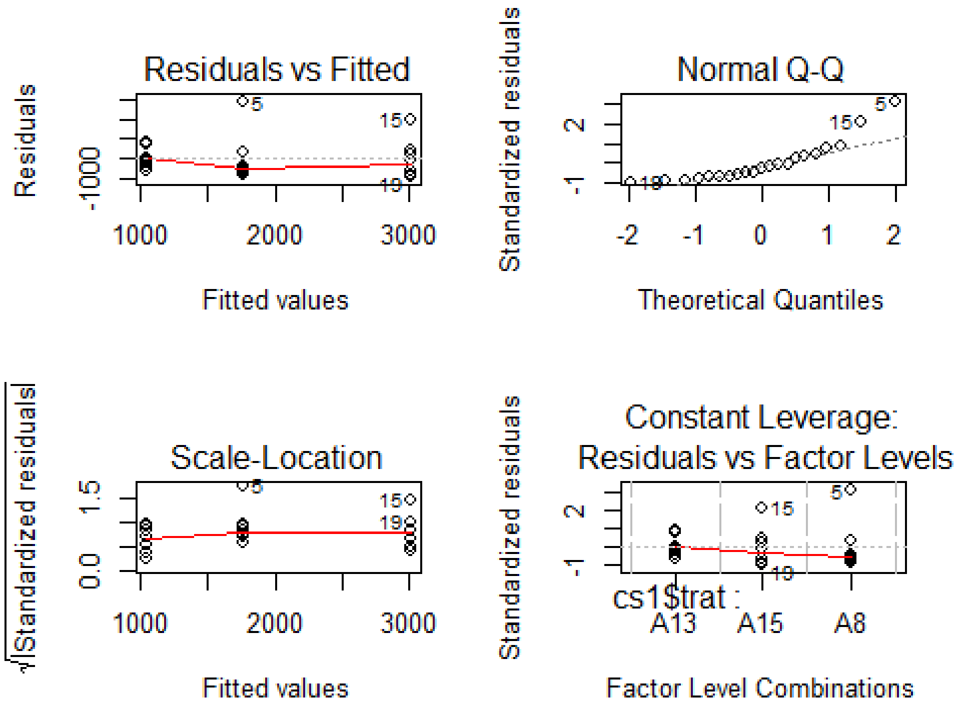


```
shapiro.test(ct$res)

##
##  Shapiro-Wilk normality test
##
## data:  ct$res
## W = 0.8106, p-value = 0.000957

# Transforma Box-Cox
boxcox(tot ~ trat, data=cs1, plotit=T)
```

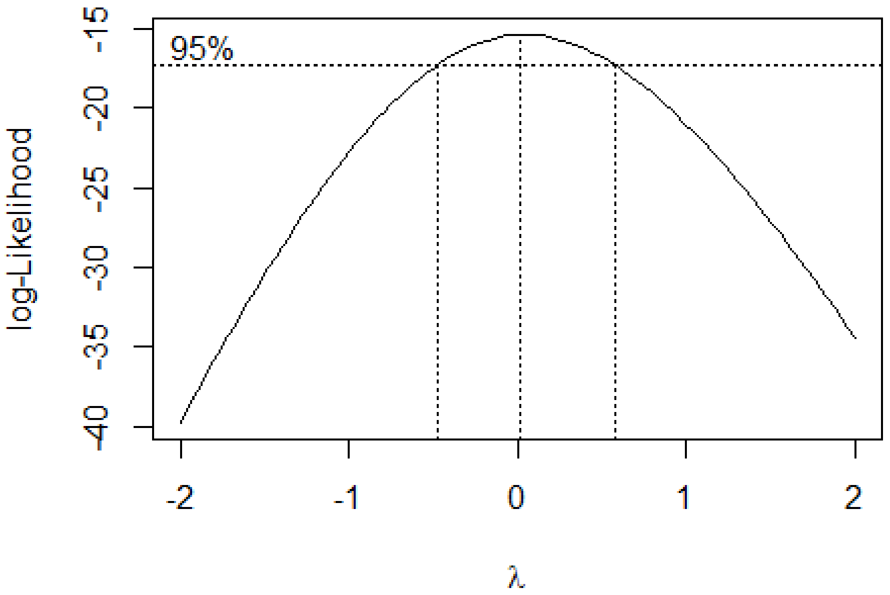


```
bc <- boxcox(tot ~ trat, data=cs1, lam=seq(-.5, .5, 1/10))
```

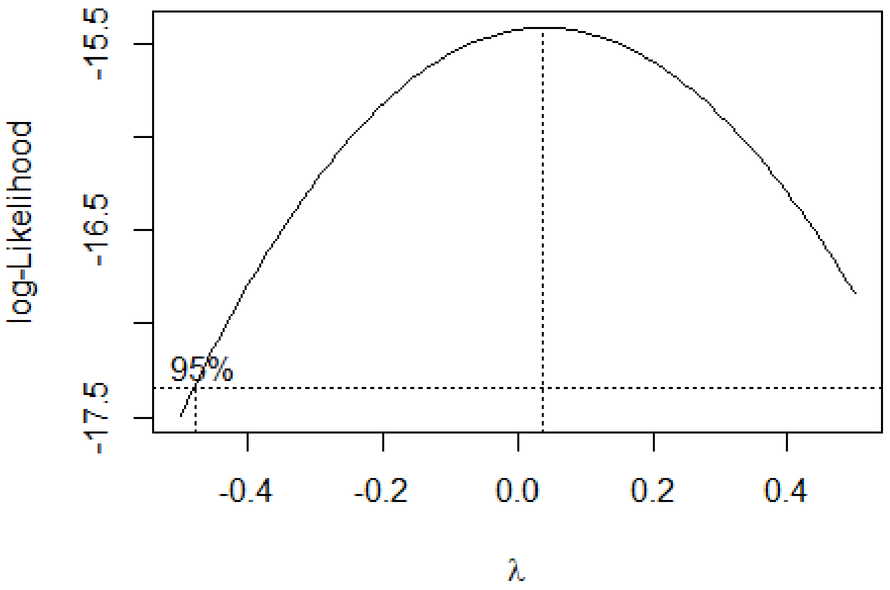


```
(lambda = bc$x[which.max(bc$y)])
```

```
## [1] 0.03535354

tl<-log(cs1$tot+0.01)

cr12<-aov(tl~cs1$trat)
cr12

## Call:
##    aov(formula = tl ~ cs1$trat)
##
## Terms:
##                 cs1$trat Residuals
## Sum of Squares  4.689068  4.345967
## Deg. of Freedom        2        18
##
## Residual standard error: 0.4913681
## Estimated effects may be unbalanced

summary(cr12)

##             Df Sum Sq Mean Sq F value  Pr(>F)
## cs1$trat     2  4.689  2.3445   9.711 0.00138 **
## Residuals   18  4.346  0.2414
## ---
## Signif. codes:  0 '***' 0.001 '**' 0.01 '*' 0.05 '.' 0.1 ' ' 1

par(mfrow=c(2,2)); plot(cr12); layout(1)
```

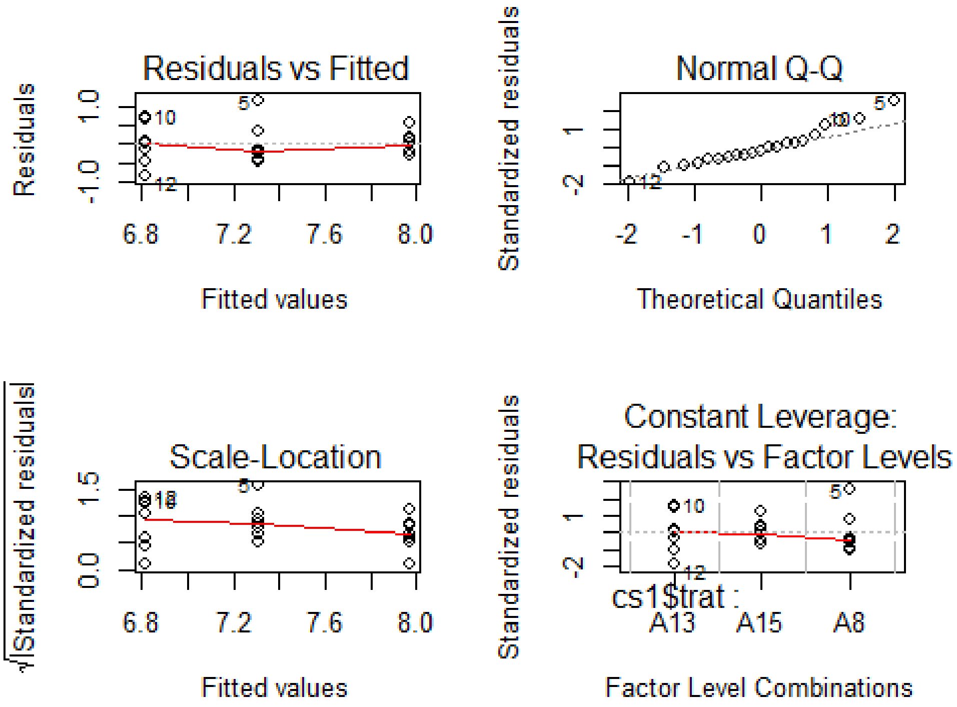

```r
shapiro.test(cr12$res)

##
##  Shapiro-Wilk normality test
##
## data:  cr12$res
## W = 0.95286, p-value = 0.3853

require(agricolae)
glr <- df.residual(cr12)
glr

## [1] 18

sqr <- deviance(cr12)
sqr

## [1] 4.345967

qmr <- sqr/glr
qmr

## [1] 0.2414426

lsdr <- LSD.test(tl,cs1$trat, glr, qmr, alpha=0.05, p.adj="none")
lsdr

## $statistics
##     MSerror Df     Mean       CV  t.value       LSD
##   0.2414426 18 7.365744 6.670991 2.100922 0.5518015
##
## $parameters
##         test p.ajusted   name.t ntr alpha
##   Fisher-LSD      none cs1$trat   3  0.05
##
## $means
##           tl       std r      LCL      UCL      Min      Max      Q25
## A13 6.815841 0.5609580 7 6.425658 7.206023 5.986477 7.528337 6.500319
## A15 7.969539 0.3076345 7 7.579357 8.359722 7.655395 8.513990 7.717313
## A8  7.311853 0.5612619 7 6.921670 7.702035 6.834120 8.444840 6.982418
##          Q50      Q75
## A13 6.821118 7.187157
## A15 7.965549 8.108608
## A8  7.118024 7.410575
##
## $comparison
## NULL
##
## $groups
##           tl groups
## A15 7.969539      a
## A8  7.311853      b
## A13 6.815841      b
```

```
##
## attr(,"class")
## [1] "group"

#reproduction factor

cs1fr<-aov(cs1$fr~cs1$trat)
cs1fr

## Call:
##    aov(formula = cs1$fr ~ cs1$trat)
##
## Terms:
##                 cs1$trat Residuals
## Sum of Squares  14.01468  18.57380
## Deg. of Freedom        2        18
##
## Residual standard error: 1.015814
## Estimated effects may be unbalanced

summary(cs1fr)

##             Df Sum Sq Mean Sq F value  Pr(>F)
## cs1$trat     2  14.02   7.007   6.791 0.00635 **
## Residuals   18  18.57   1.032
## ---
## Signif. codes:  0 '***' 0.001 '**' 0.01 '*' 0.05 '.' 0.1 ' ' 1

par(mfrow=c(2,2)); plot(cs1fr); layout(1)
```

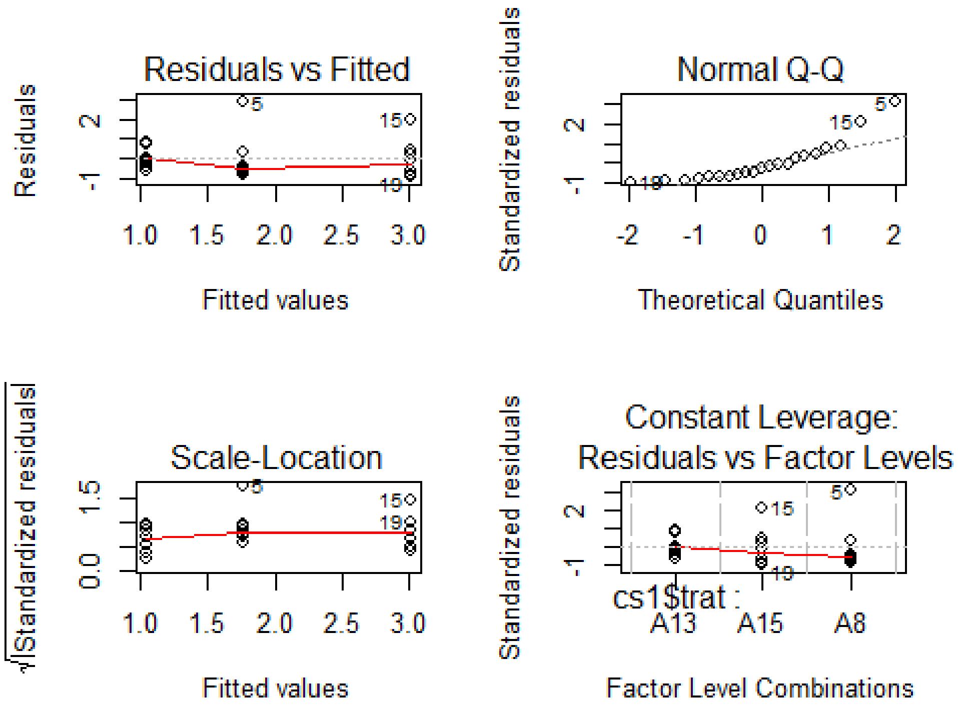


```
shapiro.test(cs1fr$res)

##
##  Shapiro-Wilk normality test
##
## data:  cs1fr$res
## W = 0.8106, p-value = 0.000957

plot(fr ~ trat, data = cs1)
```

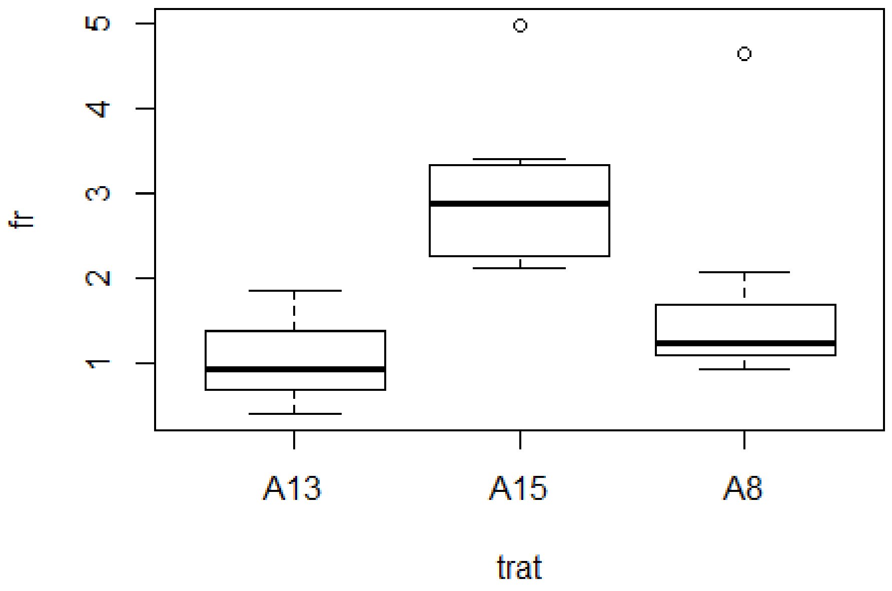


```
bartlett.test(cs1$fr, cs1$trat)

##
##  Bartlett test of homogeneity of variances
##
## data:  cs1$fr and cs1$trat
## Bartlett's K-squared = 3.6321, df = 2, p-value = 0.1627

# Transforma??o Box-Cox
boxcox(fr+0.01 ~ trat, data=cs1, plotit=T)
```

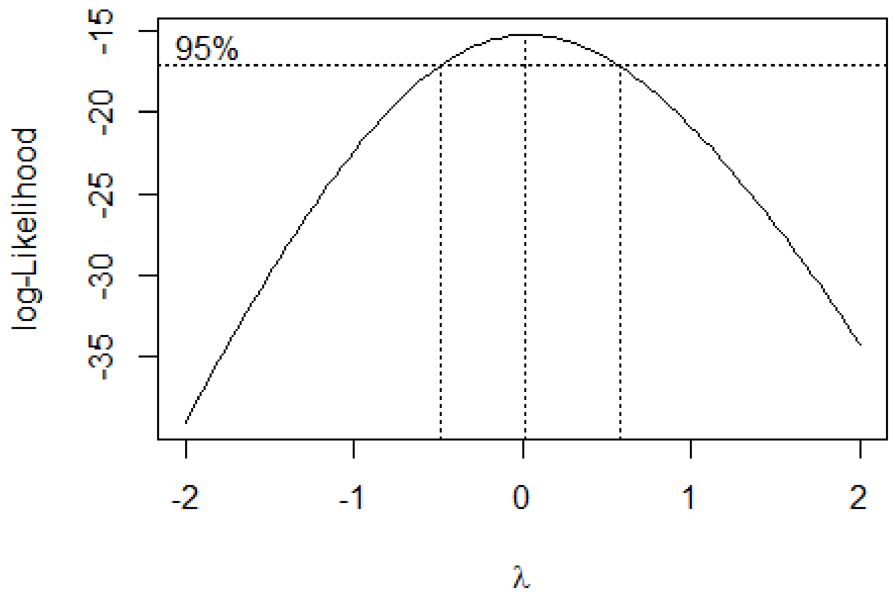


```r
bc <- boxcox(fr+0.01 ~ trat, data=cs1, lam=seq(-.5, .5, 1/10))
```

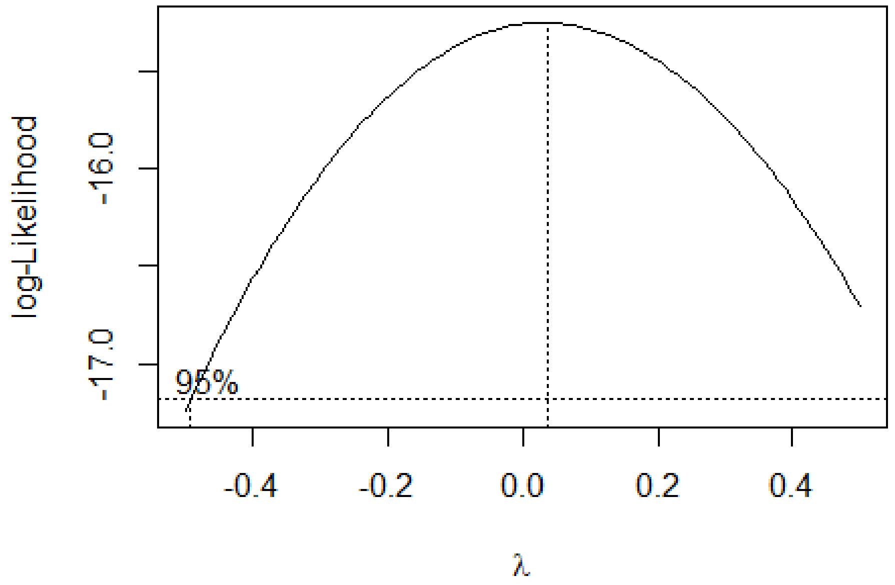


```r
(lambda = bc$x[which.max(bc$y)])
```

```
## [1] 0.03535354
```

```
fr1<-log(cs1$fr+0.01)
```

```
cs1fr1<-aov(fr1~cs1$trat)
cs1fr1
```

```
## Call:
##    aov(formula = fr1 ~ cs1$trat)
##
## Terms:
##                 cs1$trat Residuals
## Sum of Squares  4.618550  4.278314
## Deg. of Freedom        2        18
##
## Residual standard error: 0.4875286
## Estimated effects may be unbalanced
```

```
summary(cs1fr1)
```

```
##             Df Sum Sq Mean Sq F value  Pr(>F)
## cs1$trat     2  4.619  2.3093   9.716 0.00138 **
## Residuals   18  4.278  0.2377
## ---
## Signif. codes:  0 '***' 0.001 '**' 0.01 '*' 0.05 '.' 0.1 ' ' 1
```

```
par(mfrow=c(2,2)); plot(cs1fr1); layout(1)
```

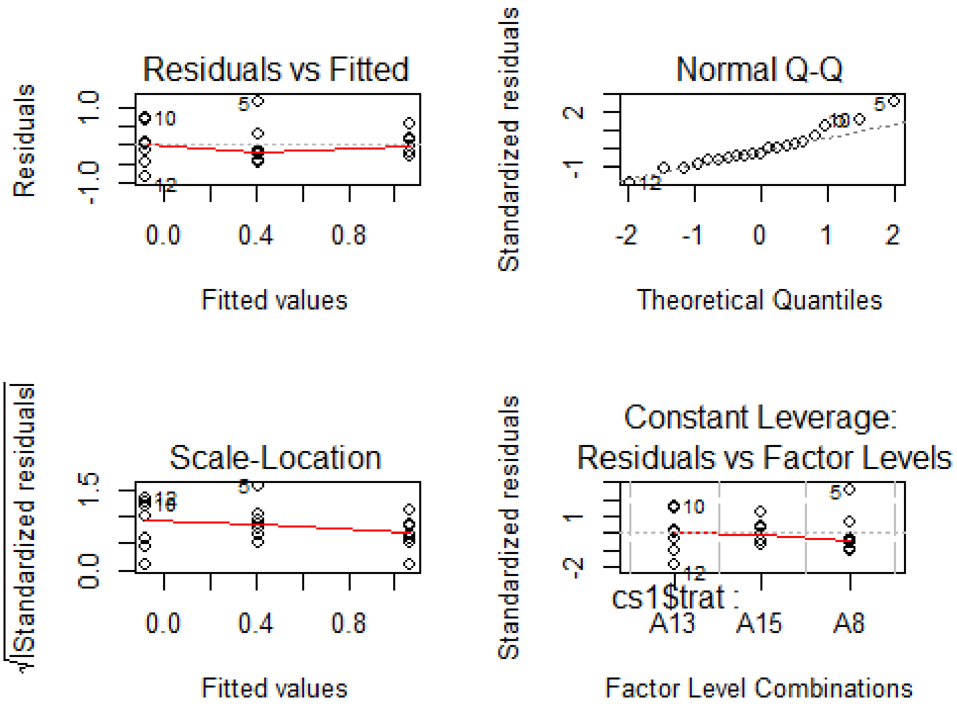


```
shapiro.test(cs1fr1$res)
```

```
##
##  Shapiro-Wilk normality test
##
## data:  cs1fr1$res
## W = 0.95199, p-value = 0.3712
```

```r
plot(fr1 ~ trat, data = cs1)
```

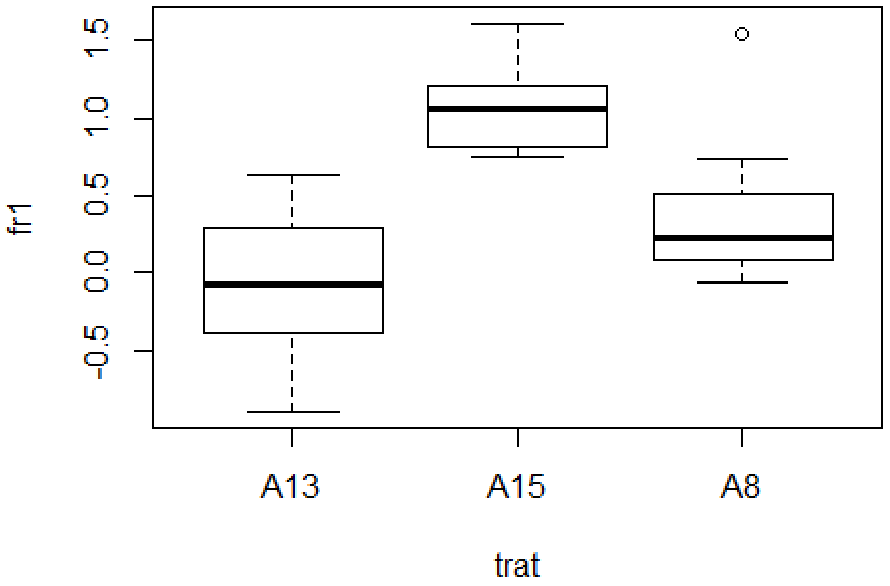


```r
bartlett.test(fr1, cs1$trat)
```

```
##
##  Bartlett test of homogeneity of variances
##
## data:  fr1 and cs1$trat
## Bartlett's K-squared = 2.2306, df = 2, p-value = 0.3278
```

```r
require(agricolae)
glr <- df.residual(cs1fr1)
glr
```

```
## [1] 18
```

```r
sqr <- deviance(cs1fr1)
sqr
```

```
## [1] 4.278314
```

```r
qmr <- sqr/glr
qmr
```

```
## [1] 0.2376841
```

```r
lsd <- LSD.test(fr1,cs1$trat, glr, qmr, alpha=0.05, p.adj="none")
lsd
```

```
## $statistics
##     MSerror Df      Mean      CV  t.value       LSD
##   0.2376841 18 0.4657813 104.669 2.100922 0.5474898
##
## $parameters
##         test p.ajusted   name.t ntr alpha
##   Fisher-LSD      none cs1$trat   3  0.05
##
## $means
##               fr1       std r         LCL       UCL        Min       Max
## A13 -0.07951039 0.5543086 7 -0.46664411 0.3076233 -0.8964881 0.6259384
## A15  1.06536616 0.3066377 7  0.67823244 1.4524999  0.7523590 1.6082372
## A8   0.41148800 0.5583615 7  0.02435428 0.7986217 -0.0629398 1.5392300
##               Q25         Q50       Q75
## A13 -0.39235500 -0.07580171 0.2872443
## A15  0.81399989  1.06125650 1.2038553
## A8   0.08390844  0.21833199 0.5089885
##
## $comparison
## NULL
##
## $groups
##               fr1 groups
## A15  1.06536616      a
## A8   0.41148800      b
## A13 -0.07951039      b
##
## attr(,"class")
## [1] "group"
```

```r
#nematodes per gram of roots

cs1n<-aov(cs1$nema~cs1$trat)
cs1n
```

```
## Call:
##    aov(formula = cs1$nema ~ cs1$trat)
##
## Terms:
##                  cs1$trat Residuals
## Sum of Squares   9498.381 23290.857
## Deg. of Freedom         2        18
##
## Residual standard error: 35.97133
## Estimated effects may be unbalanced
```

```
summary(cs1n)

##              Df Sum Sq Mean Sq F value Pr(>F)
## cs1$trat      2   9498    4749    3.67  0.046 *
## Residuals    18  23291    1294
## ---
## Signif. codes:  0 '***' 0.001 '**' 0.01 '*' 0.05 '.' 0.1 ' ' 1

par(mfrow=c(2,2)); plot(cs1n); layout(1)
```

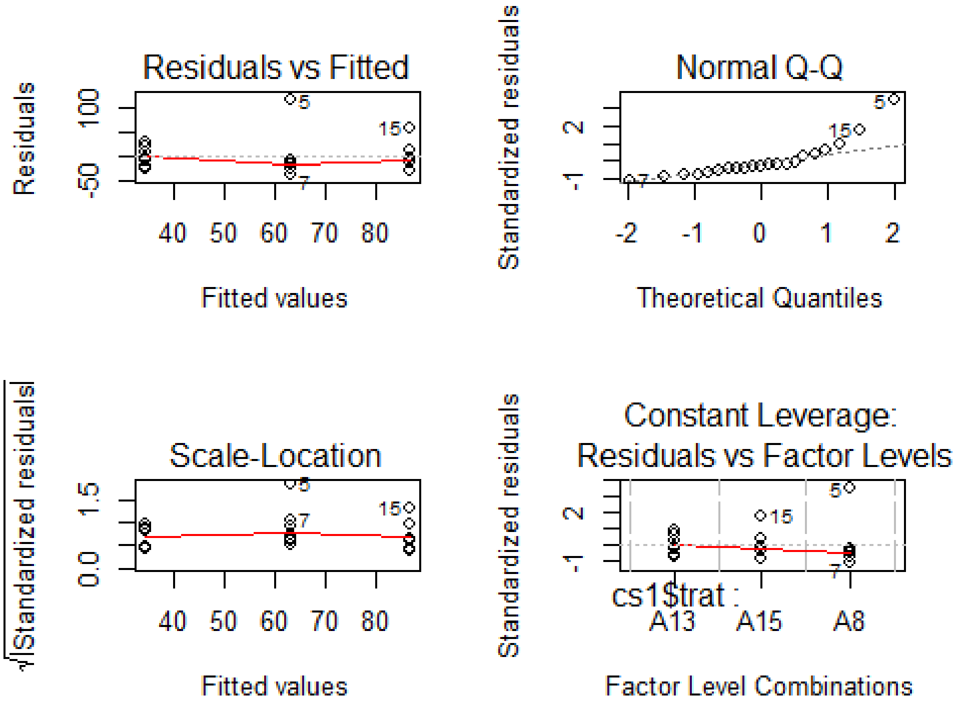


```
shapiro.test(cs1n$res)

##
##  Shapiro-Wilk normality test
##
## data:  cs1n$res
## W = 0.77358, p-value = 0.000266

plot(nema ~ trat, data = cs1)
```

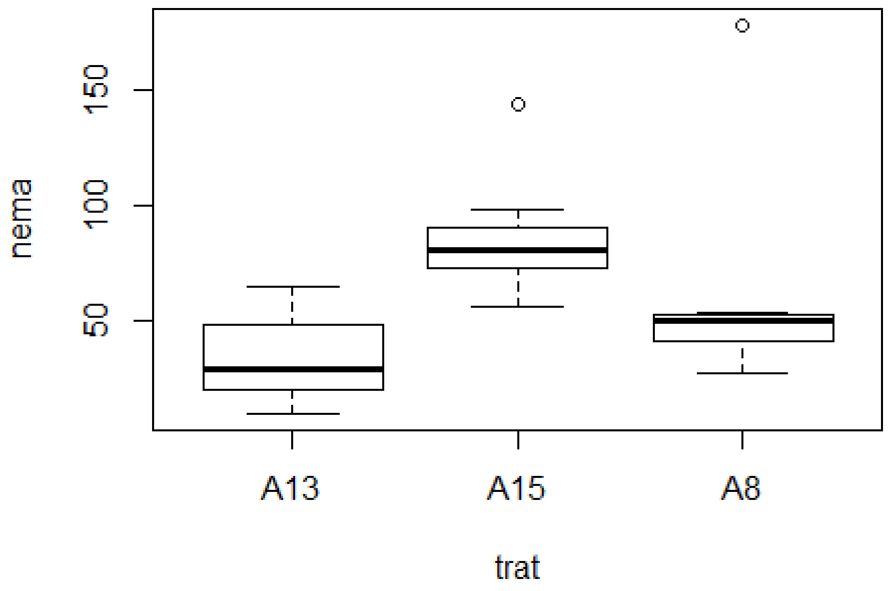


```
bartlett.test(cs1$nema, cs1$trat)

##
##  Bartlett test of homogeneity of variances
##
## data:  cs1$nema and cs1$trat
## Bartlett's K-squared = 4.8409, df = 2, p-value = 0.08888

# Transforma Box-Cox
boxcox(nema+0.01 ~ trat, data=cs1, plotit=T)
```

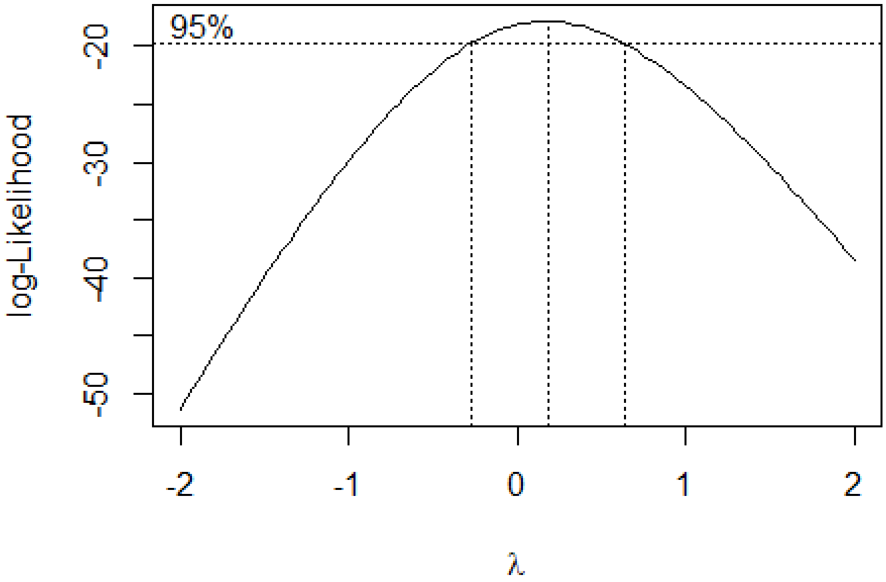


```
bc <- boxcox(nema+0.01 ~ trat, data=cs1, lam=seq(-.5, .5, 1/10))
```

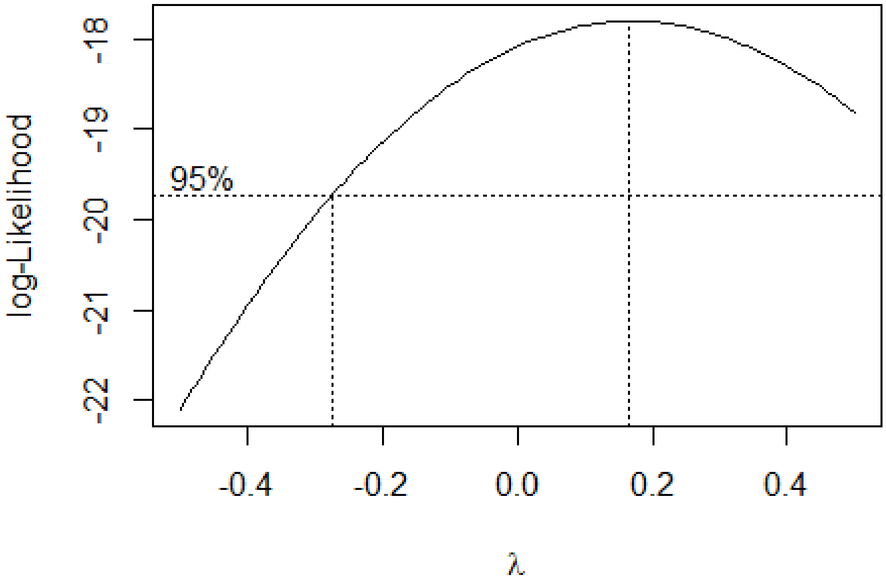


```
(lambda = bc$x[which.max(bc$y)])
```

```
## [1] 0.1666667
```

```r
n1<-log(cs1$nema+0.01)
```

```r
cs1n1<-aov(n1~cs1$trat)
cs1n1
```

```
## Call:
##    aov(formula = n1 ~ cs1$trat)
##
## Terms:
##                 cs1$trat Residuals
## Sum of Squares  4.007879  5.597761
## Deg. of Freedom        2        18
##
## Residual standard error: 0.5576618
## Estimated effects may be unbalanced
```

```r
summary(cs1n1)
```

```
##             Df Sum Sq Mean Sq F value  Pr(>F)
## cs1$trat     2  4.008   2.004   6.444 0.00775 **
## Residuals   18  5.598   0.311
## ---
## Signif. codes:  0 '***' 0.001 '**' 0.01 '*' 0.05 '.' 0.1 ' ' 1
```

```r
par(mfrow=c(2,2)); plot(cs1n1); layout(1)
```

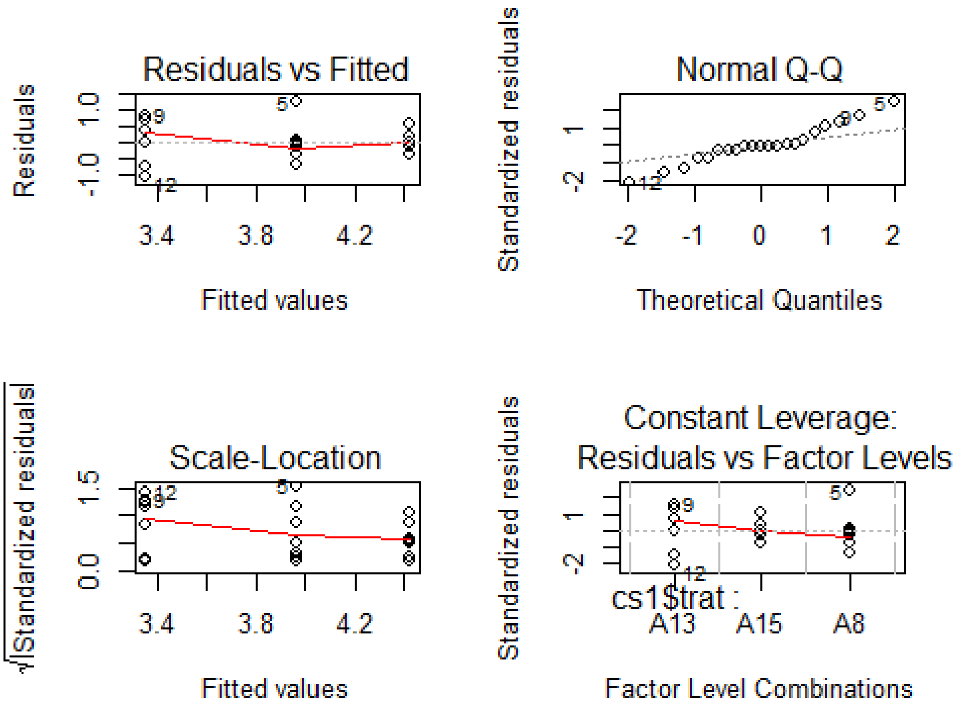


```r
shapiro.test(cs1n1$res)
```

```
##
##  Shapiro-Wilk normality test
##
## data:  cs1n1$res
## W = 0.95744, p-value = 0.4662
```

```r
plot(n1 ~ trat, data = cs1)
```

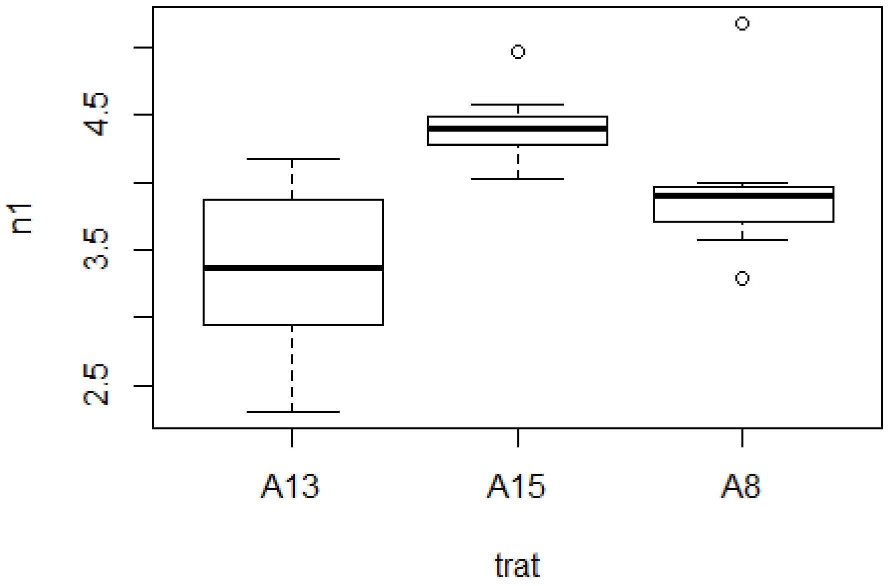


```r
bartlett.test(n1, cs1$trat)
```

```
##
##  Bartlett test of homogeneity of variances
##
## data:  n1 and cs1$trat
## Bartlett's K-squared = 3.8408, df = 2, p-value = 0.1466
```

```r
require(agricolae)
glr <- df.residual(cs1n1)
glr
```

```
## [1] 18
```

```r
sqr <- deviance(cs1n1)
sqr
```

```
## [1] 5.597761
```

```r
qmr <- sqr/glr
qmr
```

```
## [1] 0.3109867
```

```
lsdn <- LSD.test(n1,cs1$trat, glr, qmr, alpha=0.05, p.adj="none")
lsdn
```

```
## $statistics
##     MSerror Df     Mean       CV  t.value       LSD
##   0.3109867 18 3.913153 14.25096 2.100922 0.6262487
##
## $parameters
##         test p.ajusted   name.t ntr alpha
##   Fisher-LSD      none cs1$trat   3  0.05
##
## $means
##           n1       std r      LCL      UCL      Min      Max      Q25
## A13 3.354770 0.7046406 7 2.911946 3.797595 2.303585 4.174541 2.949140
## A15 4.421328 0.2953198 7 3.978504 4.864153 4.025530 4.969883 4.283701
## A8  3.963362 0.5909552 7 3.520537 4.406186 3.296207 5.181840 3.706328
##          Q50      Q75
## A13 3.367641 3.869673
## A15 4.394573 4.495955
## A8  3.912223 3.970303
##
## $comparison
## NULL
##
## $groups
##           n1 groups
## A15 4.421328      a
## A8  3.963362     ab
## A13 3.354770      b
##
## attr(,"class")
## [1] "group"
```
